# Visual and patient-reported outcomes of an enhanced versus monofocal intraocular lenses in cataract surgery: a systematic review and meta-analysis

**DOI:** 10.1038/s41433-025-03625-4

**Published:** 2025-02-01

**Authors:** Joaquín Fernández, Filomena Ribeiro, Noemí Burguera, Marina Rodríguez-Calvo-de-Mora, Manuel Rodríguez-Vallejo

**Affiliations:** 1 Qvision, Department of Ophthalmology, VITHAS Almería Hospital, Almería, Spain; 2 Departamento de Oftalmologia, Hospital da Luz, Lisbon, Portugal; 3 Faculdade de Medicina, Universidade de Lisboa, Lisbon, Portugal; 4 Ophthalmology Department, VITHAS Málaga, Málaga, Spain; 5 Hospital Regional Universitario de Málaga, Plaza del Hospital Civil, Málaga, Spain

**Keywords:** Outcomes research, Health services

## Abstract

Understanding the functional outcomes achieved with new enhanced monofocal intraocular lenses (IOLs) is crucial for adequately managing patient expectations. This study evaluated visual and patient-reported outcomes of an enhanced range of field IOL versus other monofocal IOLs in cataract patients. A systematic review and meta-analysis, pre-registered on PROSPERO (CRD42024561611), included studies from Medline (PubMed), Embase (Ovid), and trial registries (2019-2024) focused on binocular cataract surgeries with various IOL models. Primary outcomes assessed were monocular distance-corrected visual acuities (CDVA, DCIVA, DCNVA), defocus curves, and contrast sensitivity; secondary measures included binocular visual acuities and patient-reported outcomes such as spectacle independence and photic phenomena. Out of 31 studies (8 randomized clinical trials, 23 case series), high-certainty evidence indicated no significant difference in CDVA between enhanced and conventional IOLs. However, enhanced IOLs demonstrated better intermediate (DCIVA: −0.11 logMAR, CI 95%: −0.13 to −0.10) and near (DCNVA: −0.12 logMAR, CI 95%: −0.17 to −0.07) visual acuities, supported by defocus curves, though with lower-certainty evidence. No significant differences were observed in contrast sensitivity or photic phenomena, and evidence for positive dysphotopsia was moderate to low. Enhanced IOLs significantly favored intermediate-distance spectacle independence, with an odds ratio of 7.85 (CI 95%: 4.08–15.09), though no differences were observed for distance spectacle independence. Near-distance spectacle independence also favored enhanced IOLs, though with low-certainty evidence. In summary, enhanced IOLs provide improved intermediate and near visual acuities compared to conventional monofocal IOLs, though further studies are needed to confirm outcomes in contrast sensitivity and patient-reported outcomes across various enhanced monofocal IOLs.

## Introduction

Cataract surgery is one of the most common procedures worldwide, with approximately 30 million surgeries conducted in 2021 [1]. Most involve the implantation of monofocal intraocular lenses (IOLs) to restore far-distance vision, but these lenses are limited by a narrow range of field (RoF), which can impair intermediate vision [2]. In 2019, the Tecnis Eyhance IOL (Johnson & Johnson) received CE marking, claiming to enhance RoF without increasing the risk of adverse events, such as reduced far-distance visual acuity or photic phenomena, which are seen in other technologies that extend the RoF [3].

Since the introduction of Eyhance, new IOLs labeled as “Plus” have emerged, suggesting enhanced performance, but this broad term can be misleading and lead to biases when grouping IOLs in research [4]. These inconsistencies may affect the validity of systematic reviews and meta-analyses, which provide the highest level of evidence by synthesizing data across studies.

Previous systematic reviews have investigated enhanced monofocal IOLs, focusing on outcomes like distance, intermediate, and near vision, spectacle independence, and adverse effects compared to conventional IOLs [5, 6]. However, inconsistencies in study design and reporting have limited the ability to analyze all endpoints comprehensively. Some key outcomes, like the monocular defocus curve with the best distance correction, remain underexplored but are essential for classifying IOLs while minimizing confounding factors such as residual refraction and micro-monovision techniques [2].

Evaluating visual acuity alone is insufficient to capture the functional benefits of an IOL. Assessing defocus curves and contrast sensitivity offers a more comprehensive measure of visual quality across a wider range of the RoF. Similarly, patient-reported outcomes like spectacle independence, photic phenomena, and satisfaction are crucial for determining whether Eyhance meets patient expectations, which is essential for clinical recommendations and patient decision-making.

Given that the most recent systematic reviews of Eyhance included studies only up to 2022 [5, 6], this updated systematic review and meta-analysis will compare the effectiveness of Eyhance and monofocal IOLs in cataract surgery patients, focusing on visual (e.g., visual acuity, defocus curves, contrast sensitivity) and patient-reported outcomes (e.g., spectacle independence, photic phenomena, dysphotopsia, satisfaction, and likelihood to recommend the procedure).

## Methods

This study adhered to the PRISMA 2020 guidelines for reporting systematic reviews and meta-analyses, including the PRISMA-Search extension recommendations [7, 8]. The review protocol was prospectively registered on PROSPERO (CRD42024561611) in July 2024, with no subsequent changes made post-registration.

### Eligibility criteria

#### Framework

The eligibility criteria and search algorithm were structured around a PICO-ST question: In cataract surgery patients (Population), how does the Eyhance IOL (Intervention) compare to other monofocal IOLs (Comparator) in terms of visual and patient-reported outcomes (Outcome), based on randomized and non-randomized studies (Study Design) with at least one month of follow-up, published between 2019 and 2024 (Timeline).

#### Population

The included studies focused on patients who underwent binocular cataract surgery, with no exclusions based on comorbidities, specific IOL models, or variations in surgical techniques, such as corneal incisions or micro-monovision adjustments. This inclusive approach ensured a comprehensive evaluation of the intervention across diverse clinical settings, making the results broadly applicable. Subgroups of studies were analyzed based on variables pre-specified in the protocol (i.e., IOL model, magnitude of astigmatism, programmed target, and comorbidities), along with new variables identified during data extraction, to explore potential sources of bias.

#### Intervention and Comparator

The intervention group consisted of patients receiving the Eyhance IOL, selected due to its significant representation in the literature, allowing a robust analysis of its effectiveness compared to other IOLs. In the comparator group, any other monofocal IOLs were included to broaden the comparison and minimize potential biases from narrow or author-defined classifications like “Plus,” “Enhance,” or “Conventional” monofocals. This wide inclusion of comparators ensured an adequate number of studies for analysis.

#### Outcomes

Primary outcomes measured the efficacy of the IOLs, focusing on monocular distance-corrected visual acuities at far (CDVA), intermediate (DCIVA), and near (DCNVA) distances, defocus curves (DC) to evaluate visual performance across different focal points, and far-distance contrast sensitivity (CSF). Secondary outcomes included procedure efficacy and patient-reported outcomes, evaluated through binocular distance-uncorrected measures such as visual acuities at different distances (UDVA, UIVA, and UNVA), defocus curves (bDC), and contrast sensitivity (bCSF). Patient-reported outcomes captured subjective experiences, including the degree of spectacle independence (SI) achieved at far, intermediate, and near distances, and the frequency (PP) and bothersome to photic phenomena (PD) such as halos, glare, and starbursts. Additionally, overall patient satisfaction was evaluated, along with whether patients would recommend the intervention or undergo the same procedure again. Studies were included for data extraction regardless of the clinical endpoint provided; for example, binocular defocus curves were extracted whether or not authors described them as distance corrected. This differed from the protocol which was planned to extract uncorrected binocular defocus curves, but very few studies accomplished this condition.

#### Other criteria

The included studies were randomized and non-randomized, both prospective and retrospective, which provided a broad methodological base to analyze the intervention’s effects. To ensure relevant and up-to-date data, only studies published between 2019 and 2024 were considered, aligning with the introduction of the Eyhance IOL in 2019. A minimum follow-up period of around 1 month was required, excluding studies with less than 3 weeks of follow-up, which was deemed insufficient to observe reliable outcomes. Studies published in any language were included, while unpublished reports (such as conference abstracts) were not considered.

### Search strategy and information sources

A systematic search was conducted across several electronic databases and clinical trial registries to identify studies meeting the eligibility criteria. The initial search was carried out using the IOLEvidence Database (IOL Evidence App, Indaloftal SL) to find studies related to Eyhance IOL. After retrieving relevant studies, keyword exploration and index term analysis was performed using PubReMiner (Jan Koster, AMC) to identify common terms used in titles and abstracts. These terms were incorporated into the final search algorithm.

The search strategy was developed using a PICO-ST framework (see Supplementary File A) and applied to optimize both the scope and sensitivity of the searches in MEDLINE (PubMed), with no language restrictions. A date range filter was applied from 2019 (the launch date of Eyhance) to 2024 (the search date). To maintain consistency across databases, the search strategy was translated for use in EMBASE (Ovid).

Additionally, clinical trial registries, including ClinicalTrials.gov, the Cochrane Central Register of Controlled Trials (CENTRAL), and the World Health Organization (WHO, https://trialsearch.who.int), were searched to identify ongoing and unpublished studies. This was done to minimize publication bias and ensure the inclusion of relevant data not yet published in peer-reviewed journals. The database searches were completed on June 24th, 2024.

### Study selection and data collection

After the search, all identified citations were imported into Rayyan (Qatar Computing Research Institute, Doha, Qatar) for reference management. No automation features were employed. Duplicates were identified and removed to ensure the uniqueness of each reference. Two independent reviewers initially screened the titles and abstracts of the remaining references against predefined eligibility criteria. Studies that did not meet these criteria were excluded. Full-text articles of the remaining citations were subsequently reviewed in detail by the same two reviewers, who rigorously applied the inclusion criteria. Any disagreements arising during the selection process were resolved through discussion, with a third reviewer acting as an arbitrator when required.

For data collection, two independent reviewers extracted relevant data from each included study using a pre-designed data extraction tool. The extracted data focused on primary and secondary outcomes, as well as other variables and potential sources of bias, including confounding factors (e.g., comorbidities, procedure modifications, among others, dataset available from the authors upon request). In cases where plots such as defocus curves and contrast sensitivity charts were provided, one reviewer used WebPlotDigitizer (Ankit Rohatgi) to digitize the data, which was then validated by a second reviewer. Any discrepancies between reviewers were resolved through consensus, with a third reviewer available for arbitration when necessary. If any data were missing or unavailable, it was labeled as “Not Available” (NA). Additional data was not required to be asked to study investigators.

### Risk of bias

The risk of bias was assessed independently by two reviewers using the RoB 2.0 tool for randomized controlled trials (RCTs) and the ROBINS-I tool for non-randomized studies of interventions [9, 10]. For RCTs, the RoB 2.0 tool evaluated five domains: bias arising from the randomization process, bias due to deviations from intended interventions, bias due to missing outcome data, bias in the measurement of the outcome, and bias in the selection of the reported result. Each study was classified into one of three categories: ‘low’, ‘some concerns’, or ‘high’. Studies identified as having a ‘high’ risk in at least one domain or ‘some concerns’ across multiple domains were deemed to have a higher overall risk of bias. Data entry and visualizations were facilitated using the RoB 2 Excel tool [11]. For non-randomized studies, the ROBINS-I tool assessed the risk of bias across seven domains, including bias due to confounding, participant selection, classification of interventions, deviations from intended interventions, missing data, measurement of outcomes, and selection of reported results. Each study was classified into one of four categories: ‘Low’, ‘Moderate’, ‘Serious’, or ‘Critical’. Discrepancies between reviewers were resolved through discussion, with a third reviewer consulted when necessary. The results were synthesized in risk of bias tables and considered in the interpretation of study findings to inform the strength of the evidence.

### Data synthesis and analyses

Eligible studies were assessed by tabulating key characteristics, such as interventions and outcomes, and comparing these with the predefined eligibility criteria outlined in the protocol. Only studies that met these criteria were included in the final synthesis. A protocol modification was made to account for variations in the reporting of binocular defocus curves. Although the original protocol specified the inclusion of uncorrected binocular defocus curves, many studies reported outcomes using best distance correction. As a result, both uncorrected and distance-corrected outcomes were included in the analysis, and any potential bias arising from this variation was carefully examined.

The characteristics of the included studies were summarized in a table, and statistical meta-analyses were performed using Comprehensive Meta-Analysis (CMA, Version 4.0). Custom tools developed by one of the authors were used to visualize the data, integrating risk of bias assessments into forest plots. For dichotomous outcomes, effect sizes were expressed as odds ratios, while mean differences were used for continuous outcomes, with 95% confidence intervals calculated for each analysis. Visual acuity and contrast sensitivity outcomes were analyzed in standard units, using logMAR and logCS, respectively. In cases where studies did not report standard deviations for these metrics, commonly used values of 0.1 logMAR and 0.2 logCS were imputed, as these are frequently reported in clinical studies and are typically used for sample size calculations [12]. Patient-reported outcomes, often measured using Likert scales, were dichotomized by grouping responses into categories such as “bothered” or “very bothered” for photic phenomena and “satisfied” or “very satisfied” for overall satisfaction. Spectacle independence was analyzed as a dichotomous outcome, with patients either reporting complete independence from spectacles or not.

A random-effects model using the DerSimonian and Laird estimator was employed for the meta-analysis, as variability in the IOLs used in the comparison groups, differences in clinic populations, testing protocols, and age distributions were expected to contribute to heterogeneity beyond sampling error. The inverse-variance method was used to pool effect sizes, and statistical heterogeneity was assessed using 95% prediction intervals, the χ^2^ test, and the *I*^2^ statistic. These heterogeneity measures were presented in the forest plots for each analysis. Subgroup analyses were conducted using a mixed-effects model, which applied a random-effects model for within-group comparisons and a pooled tau-squared value for the overall effect [13].

Sensitivity analyses were performed to assess the robustness of the results by examining sources of bias identified during the review. These included studies that involved patients with several magnitudes of corneal astigmatism, as well as patients with ocular comorbidities (e.g., glaucoma, diabetic retinopathy, macular degeneration). Additional sources of variation included differences in the IOLs used in the comparator group, mean age of participants, outcome measurement methods, and follow-up durations. Furthermore, meta-regression analyses were performed to explore the impact of continuous variables, such as age, on the study outcomes.

### Meta-bias and confidence in cumulative evidence

A funnel plot was created and examined to investigate potential small-study biases if more than ten studies could be pooled in a single meta-analysis. The number of studies missing from the funnel plot was estimated using the trim-and-fill method [14]. Additionally, publication bias was assessed using Begg’s rank correlation and Egger’s weighted regression method test [15, 16]. These methods were selected to detect potential asymmetry in the funnel plot and quantify the likelihood of smart study bias. Any discrepancies in assessments were resolved by consensus between two independent reviewers. In cases where statistical pooling was not feasible, findings are presented in narrative form.

The quality of evidence for each outcome was assessed using the GRADE approach [17]. The criteria for downgrading the quality of evidence included risk of bias, inconsistency, imprecision, indirectness, and others. Where appropriate, evidence was upgraded based on factors such as the magnitude of effect, and the absence of plausible confounding. Two independent reviewers conducted the GRADE assessments, with disagreements resolved through discussion or referral to a third reviewer.

## Results

### Included studies

**Fig. 1:**
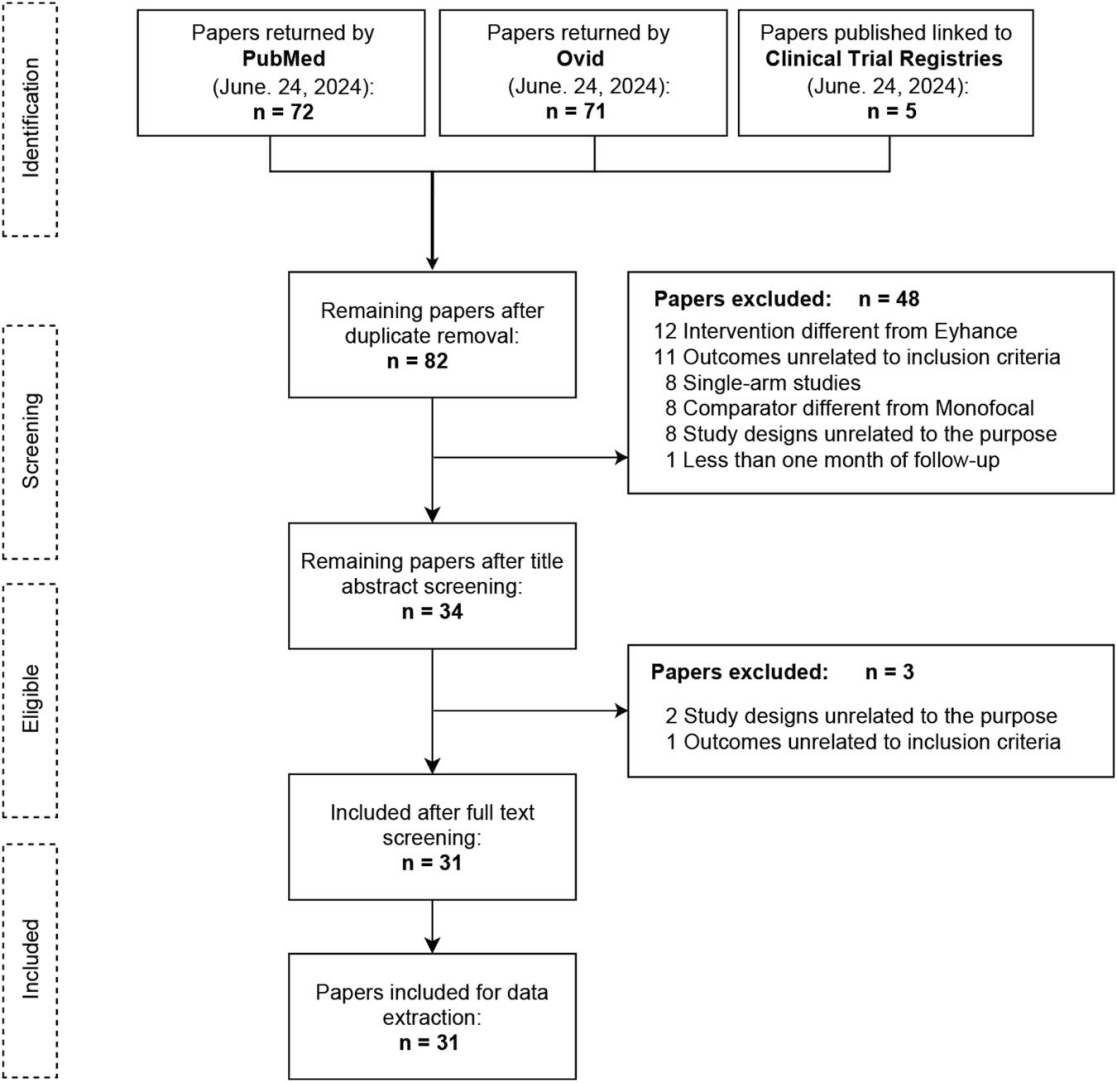
PRISMA flow diagram for systematic reviews, illustrating the process of database and registry searches.

A total of 148 records were retrieved from the initial systematic search and clinical trial databases, which was reduced to 82 after removing duplicates. The first screening of titles and abstracts resulted in the exclusion of 48 references for various reasons, as outlined in Fig. 1. The most common reasons for exclusion were interventions different from the Eyhance IOL and outcomes unrelated to those described in the eligibility criteria. An additional three studies were excluded after full-text screening, leaving a total of 31 studies for data extraction.

### Studies description

Eligible trials were described as randomized clinical trials (*n* = 8) and comparative case series, either prospective (*n* = 10), retrospective (*n* = 12) or cross-sectional (*n* = 1). Two studies compared Eyhance with more than one monofocal IOL, Mencucci et al. [18] and Giglio et al. [19], these were differentiated by adding an additional letter after the year (i.e. Mencucci et al. [18]a or Mencucci et al. [18]b for Tecnis PCB00 or Clareon CNA0T0 comparison, respectively). Characteristics of the study population such as inclusion criteria for corneal astigmatism, astigmatism management with different types of IOLs and corneal incisions, mean age of the intervention group, eye comorbidities, and variations of targeting such with techniques such as micro-monovision are detailed in Table 1. The complete extraction for all the variables is available from authors upon request. From studies for which data were extracted only Hwang et al. [20] did not report any of the primary or secondary endpoints described in the protocol. In addition, Singh [21] was not considered in the DCIVA analysis since the outcome (better than 0 logMAR in both groups) suggests that corrected intermediate visual acuity (CIVA) was measured instead even though it was reported as DCIVA. This also was done for the DCNVA and Donoso [22] study for the same reason but for near distance.

**Table 1:** Characteristics of the included studies.

Study	Study design	Comparator	Corneal astigmatism (D)	Astigmatism management	Mean age (years)	Comorbidities	Targeting refraction	ETDRS used	Luminance (85 cd/m^2^)
Choi et al. [26]	RCT	Tecnis ZCB00	≤1.25	Spherical + NR	70.9	None	Emmetropia	Yes	NR
Eguileor et al. [37]	RCT	Tecnis ZCB00	≤1.5	Spherical + NR	72.2	None	Emmetropia	Yes	Yes
Giglio et al. [19]a	RCT	Tecnis PCB00	≤0.75	Spherical + NR	75.73	None	Emmetropia	Yes	Yes
Giglio et al. [19]b	RCT	Clareon CNA0T0	≤0.75	Spherical + NR	75.73	None	Emmetropia	Yes	Yes
Goslings et al. [38]	RCT	Vivinex iSert	≤1	Spherical + Temporal CCI	72.4	None	NR	Yes	NR
Donoso et al. [22]	RCT	Tecnis ZCB00	≤1.5	Spherical + NR	71	None	Emmetropia	Yes	NR
Garzón et al. [39]	RCT	Tecnis ZCB00	≤1.5	Spherical + Temporal CCI	75.87	None	Emmetropia	Yes	Yes
Nanavaty et al. [40]	RCT	RayOne Monofocal	≤2.50	Spherical + PCRIs if >0.75 D	72.79	None	Emmetropia	Yes	Yes
Auffarth et al. [41]	RCT	Tecnis ZCB00	≤1	Spherical + NR	69.3	None	Emmetropia	Yes	Yes
Salgado-Borges et al. [24]	Pros CS	IsoPure	NR	Spherical + NR	NR	None	NR	NR	NR
Giansanti et al. [42]	Pros CS	Tecnis ZCB00	≤0.75	Spherical + NR	74.54	EPR	Hyperopic	Yes	Yes
Mencucci et al. [3]	Pros CS	Tecnis ZCB00	≤0.75	Spherical + NR	72.31	None	Hyperopic	Yes	Yes
Singh et al. [21]	Pros CS	Tecnis ZCB00	≤0.75	Spherical + NR	61.3	None	NR	NR	NR
Steinmüller et al. [31]	Pros CS	Tecnis ZCB00	≤0.75	Spherical + Steep CCI	65.8	None	Emmetropia	Yes	NR
Nam et al. [25]	Pros CS	Tecnis ZCB00	≤0.75	Spherical + NR	68.76	Early Glaucoma	Emmetropia	Yes	Yes
Gigon et al. [43]	Pros CS	Tecnis PCB00	≤2	Spherical + Steep CCI	76	None	Emmetropia	NR	NR
Micheletti et al. [30]	Pros CS	Clareon CCA0T0 or CNA0T0	NR	Spherical or Toric only in intervention group + NR	69.54	None	Emmetropia, Mini-monovision, or Monovision	NR	NR
Corbelli et al. [32]	Pros CS	Tecnis ZCB00	≤0.75	Spherical + Temporal CCI	72.7	None	NR	Yes	Yes
Mencucci et al. [44]	Retros CS	Tecnis ZCB00	≤0.75	Spherical + NR	65.32	EK	Emmetropia	Yes	Yes
Mencucci et al. [18]a	Retros CS	Vivinex Impress	≤0.75	Spherical + Temporal CCI	79.38	None	Emmetropia	Yes	Yes
Mencucci et al. [18]b	Retros CS	IsoPure	≤0.75	Spherical + Temporal CCI	79.38	None	Emmetropia	Yes	Yes
Hwang et al. [20]	Retros CS	Tecnis AAB00	>1	Toric + Steep CCI	67	Retinal Disorders	Emmetropia	NR	NR
Dell et al. [33]	Retros CS	Tecnis ZCB00	≤1.5	Spherical + NR	60.07	None	Emmetropia or Mini-monovision	Yes	NR
Corbelli et al. [23]	Retros CS	Zoe Primus-HD	≤0.75	Spherical + NR	72.4	None	Emmetropia	Yes	Yes
Lopes et al. [27]	Retros CS	Tecnis PCB00	≤1	Spherical + Steep CCI if >0.75 D	71	None	Emmetropia	NR	NR
Ucar et al. [45]	Retros CS	Tecnis ZCB00	≤1	Spherical + NR	59.83	None	Emmetropia	NR	NR
Huh et al. [46]	Retros CS	Tecnis ZCB00	NR	Spherical + NR	69.6	None	Emmetropia	NR	NR
Hae Kang et al. [47]	Retros CS	Tecnis ZCB00	≤1	Spherical + Steep CCI	65.23	None	Emmetropia	NR	NR
Cinar et al. [48]	Retros CS	AcrySof SN60WF	≤1	Spherical + NR	61.3	None	NR	Yes	NR
Unsal et al. [29]	Retros CS	Tecnis ZCB00	≤1	Spherical + Steep CCI	56.2	None	NR	NR	NR
Beltraminelli et al. [49]	Retros CS	AcrySof SN60WF or ZCB00	NR	Spherical + NR	75.2	None	Mini-monovision	NR	NR
Kozhaya et al. [50]	Cross-Sectional CS	SofPort or enVista or Toric	NR	Spherical or Toric + NR	69.7	None	NR	NR	NR
Elbakry [28]	Retros CS	Tecnis ZCB00	≤0.75	Spherical + Sup -Temp CCI	MR	None	NR	Only Far	NR

ETRS: “Yes” means that the standard chart was used for visual acuity measurements; Luminance: “Yes” means that the authors reported measuring visual acuity with a background luminance chart of 85 cd/m^2^.

*RCT* Randomized Clinical Trial, *Pros CS* Prospective Case Series, *Retros CS* Retrospective Case Series, *EPR* Epiretinal Membrane Treated by Phaco-Vitrectomy, *EK* Triple Descemet Membrane Endothelial Keratoplasty, *NR* Not reported, *CCI* Clear Corneal Incision.

### Risk of bias

The risk of bias was evaluated at the outcome level, recognizing that different confounders can affect specific endpoints, potentially limiting confidence in efficacy estimates. For example, the failure to report postoperative residual refractive error contributed to the risk of bias for UIVA but not for DCIVA. Therefore, the risk of bias was incorporated into the forest plots using both the RoB 2 and ROBINS-I tools, acknowledging differences in the number of domains assessed. Specifically, domains F and G were marked as “not available” (NA) when applying the RoB 2 tool to RCTs. Supplementary File B provides detailed reasons for the judgments in each domain and endpoint, while Supplementary File C displays weighted bar plots showing the distribution of risk-of-bias judgments within each domain.

Critical risk of bias was more frequent in case series than in RCTs, primarily due to confounding, and more common in studies reporting binocular outcomes without distance correction or patient-reported outcomes (~50%) compared to those reporting monocular outcomes with distance correction (~25%) (see Supplementary File C). In non-randomized studies assessed with the ROBINS-I tool, confounding was frequently observed, such as a lack of reporting on demographic variables or comorbidities that might influence outcomes. Selection bias was also common, with many studies failing to detail participant selection or excluding patients with complications. Some studies introduced classification issues when multiple types of IOLs were used for the comparator group. Deviations from intended interventions were often poorly described, particularly regarding how surgeries were performed or re-interventions were handled. Additionally, some studies failed to report the absence of missing data, raising concerns about moderate bias. Standard methods for measuring outcomes were sometimes not properly reported, leading to critical bias. Incomplete reporting of outcomes also emerged as a recurring issue, with some studies omitting key outcomes or using non-standard analysis methods.

In RCTs evaluated with the RoB 2 tool, common sources of bias included insufficient reporting on randomization procedures, with missing details on sequence generation and allocation concealment. Baseline differences between intervention groups, such as age, were also noted. Deviations from intended interventions were observed, including unclear masking of participants, carers, and assessors, and underreporting of adverse events. While missing outcome data were often deemed negligible, they were not always clearly addressed. Measurement bias was also a concern, especially when assessors, aware of the intervention, may have influenced outcome thresholds, despite using standard charts. Lastly, concerns about selective reporting arose, as some studies lacked publicly available protocols. These biases, particularly confounding, suggest caution when interpreting Eyhance efficacy, especially for binocular outcomes without distance correction or patient-reported outcomes.

### Primary outcomes and sensitivity analysis

#### Monocular distance-corrected visual acuities

**Fig. 2:**
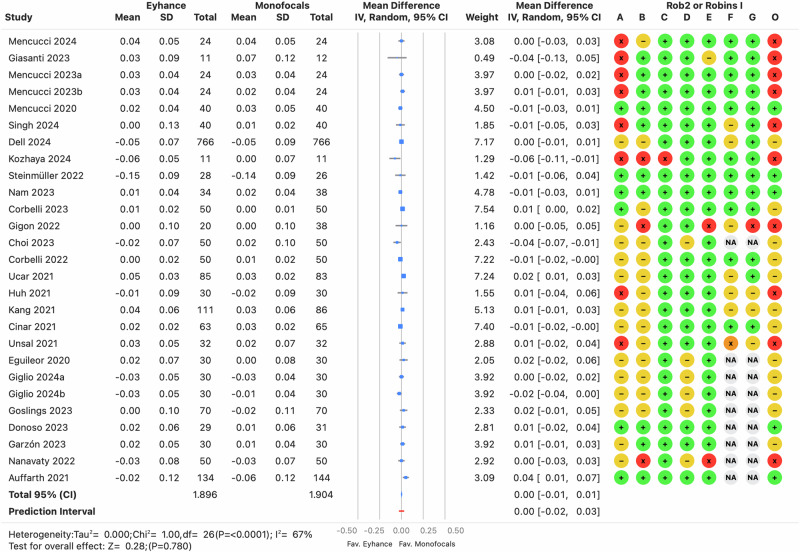
Forest plot of monocular corrected distance visual acuity outcome. Mean differences from individual studies (blue squares) are displayed in rows, with confidence intervals (CI) represented by horizontal lines. The pooled mean difference from all studies is shown as a diamond, extending to the pooled CI. A red line indicates the prediction interval. Colored circles next to each study represent the risk of bias for each domain (A to G, based on RoB 2 or ROBINS-I) and the overall bias (O). Meta-analysis statistics are summarized at the bottom left.

**Fig. 3:**
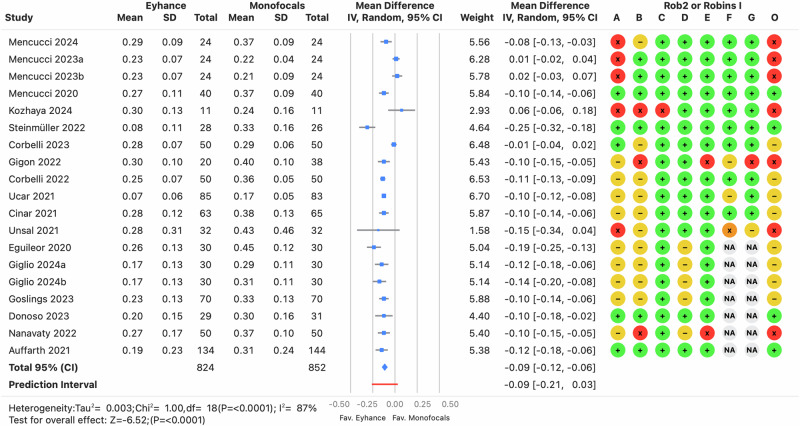
Forest plot of monocular distance-corrected intermediate visual acuity outcome. Mean differences from individual studies (blue squares) are displayed in rows, with confidence intervals (CI) represented by horizontal lines. The pooled mean difference from all studies is shown as a diamond, extending to the pooled CI. A red line indicates the prediction interval. Colored circles next to each study represent the risk of bias for each domain (A to G, based on RoB 2 or ROBINS-I) and the overall bias (O). Meta-analysis statistics are summarized at the bottom left.

**Fig. 4:**
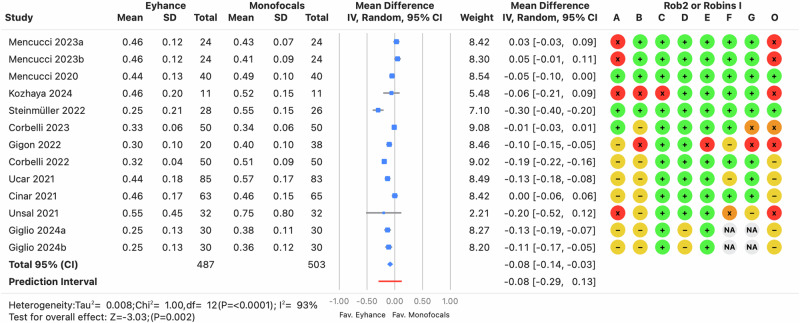
Forest plot of monocular distance-corrected near visual acuity outcome. Mean differences from individual studies (blue squares) are displayed in rows, with confidence intervals (CI) represented by horizontal lines. The pooled mean difference from all studies is shown as a diamond, extending to the pooled CI. A red line indicates the prediction interval. Colored circles next to each study represent the risk of bias for each domain (A to G, based on RoB 2 or ROBINS-I) and the overall bias (O). Meta-analysis statistics are summarized at the bottom left.

Monocular distance-corrected visual acuities are a key indicator of the IOL’s efficacy, as procedural modifications are minimized, reducing confounding factors such as postoperative residual refractive errors or binocular summation. However, the *I*^2^ was high (≥64%, *p* < 0.0001) across the three distances, indicating that the variance in effects was greater than expected from random variability alone. There was no clinically relevant bias for CDVA, with prediction intervals ranging from −0.02 to 0.03 logMAR (see Fig. 2), suggesting differences of one letter or less on a visual acuity chart with five letters per row. In contrast, differences in DCIVA and DCNVA were clinically relevant, with pooled estimates of −0.09 and −0.08 logMAR, respectively, favoring Eyhance for both DCIVA (see Fig. 3) and DCNVA (see Fig. 4) (*p* < 0.0001).

A subgroup analysis demonstrated that classifying Zoe Primus-HD (Corbelli et al. [23]), Vivinex Impress (Mencucci et al. [18]a), and IsoPure (Mencucci et al. [18]b) as Enhanced IOLs, along with the Eyhance IOL, revealed no significant differences in either DCIVA or DCNVA (see Supplementary Figs. A and B, respectively) and led to a decrease in *I*^2^. However, when these Enhanced IOLs were compared with the remaining monofocal IOLs (Tecnis ZCB00, PCB00, or AAB00; Clareon CNA0T0 or CCA0T0; Vivinex iSert; RayOne Monofocal; AcrySof SN60WF; and SofPort, enVista, or Toric), the differences increased to −0.11 logMAR and −0.12 logMAR for both DCIVA and DCNVA (*p* < 0.0001), respectively. Exploration of other variables, including age, corneal astigmatism inclusion criteria, and comorbidities, revealed that only the use of standard measurement methods (ETDRS at 85 cd/m²) significantly reduced *I*² from 54% (*p* = 0.005) to 20% (*p* = 0.27) for DCIVA in the subgroup excluding the three previous described IOLs. However, no clinically relevant differences (<0.02 logMAR) were found between studies using standard versus non-standard testing methods. Overall, the included studies were at low to moderate risk of bias (see Supplementary File C). A subgroup analysis of risk of bias revealed an underestimation of mean differences for DCIVA in studies with serious, high, or critical bias (−0.08, CI: −0.12 to −0.05) compared to other bias levels (−0.12, CI: −0.14 to −0.10) logMAR, and similarly for DCNVA (−0.06, CI: −0.16 to 0.03) versus (−0.12, CI: −0.17 to −0.06).

#### Monocular distance-corrected defocus curve

**Fig. 5:**
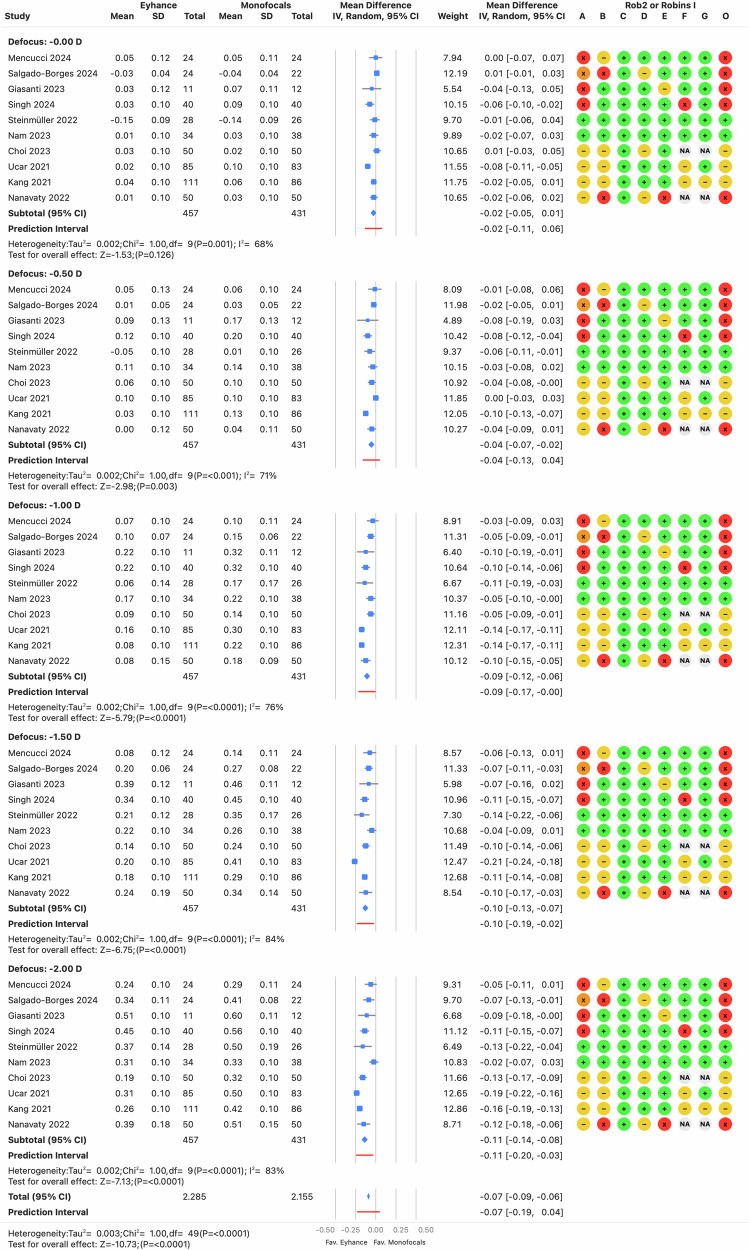
Forest plot of monocular defocus curve. Mean differences from individual studies (blue squares) are displayed in rows, with confidence intervals (CI) represented by horizontal lines. The pooled mean difference from all studies is shown as a diamond, extending to the pooled CI. A red line indicates the prediction interval. Colored circles next to each study represent the risk of bias for each domain (A to G, based on RoB 2 or ROBINS-I) and the overall bias (O). Meta-analysis statistics are summarized at the bottom left.

Figure 5 displays the forest plot for subgroups based on the defocus lens from the monocular DC, using the best distance correction. The defocus range analyzed was 0 to −2 D, as all studies reporting the defocus curve covered this range, whereas some lacked data beyond −2.0 D. Notably, only five studies reported standard deviations. Therefore, a standard deviation of 0.1 logMAR was assumed for studies without this information, as outlined in the “Methods” section. A subgroup analysis, limited to the studies that reported standard deviations, confirmed the same conclusions as the overall analysis. Statistically significant differences between Eyhance and monofocal IOLs were observed from −0.5 D to −2.0 D, with a slight increase in effect as defocus increased. Clinically relevant differences were found for defocus levels beyond −1.0 D, and these differences were also significant when compared to IsoPure (Salgado-Borges [24]). Similar to the measurement of proximal visual acuities, the *I*² value was high (≥68%, *p* < 0.0001), indicating substantial heterogeneity and the risk of bias was balanced between serious, high, or critical bias and low to moderate bias. Some studies showing less pronounced differences between groups involved in either critical bias or a population with ocular comorbidities (Nam [25]).

#### Monocular distance-corrected contrast sensitivity

**Fig. 6:**
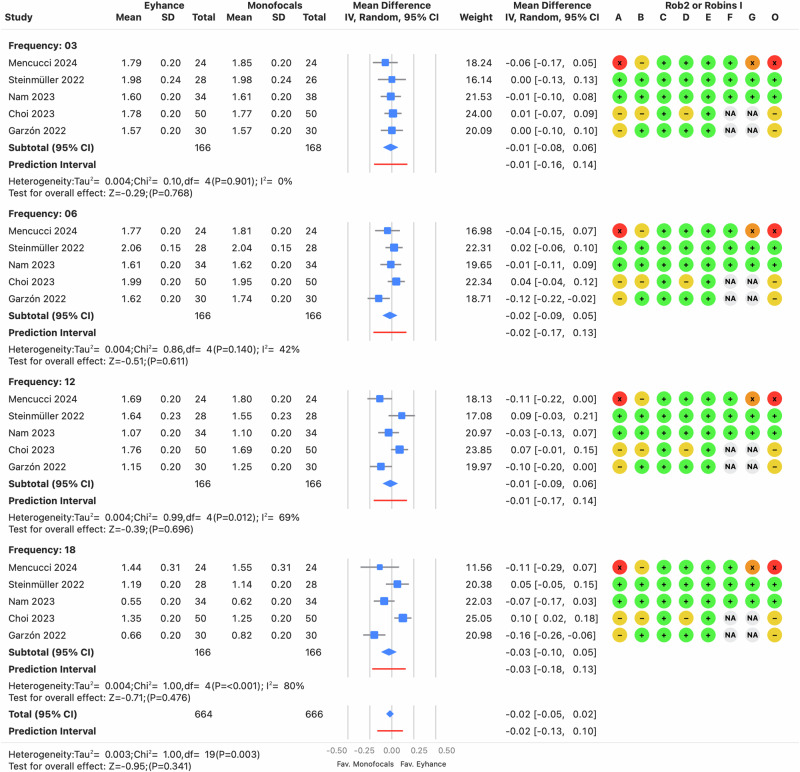
Forest plot of monocular contrast sensitivity function. Mean differences from individual studies (blue squares) are displayed in rows, with confidence intervals (CI) represented by horizontal lines. The pooled mean difference from all studies is shown as a diamond, extending to the pooled CI. A red line indicates the prediction interval. Colored circles next to each study represent the risk of bias for each domain (A to G, based on RoB 2 or ROBINS-I) and the overall bias (O). Meta-analysis statistics are summarized at the bottom left.

Few studies reported monocular distance-corrected CSF, particularly under mesopic or glare conditions, with no more than three studies providing outcomes. The photopic condition without glare had the most data, with five studies, but significant limitations were noted, as only two studies reported standard deviations. As a result, a standard deviation of 0.2 logCS was assumed to pool the data. Figure 6 shows no significant differences between Eyhance and monofocal IOLs, particularly for low and middle spatial frequencies.

### Secondary outcomes

#### Binocular uncorrected visual acuities

Binocular uncorrected visual acuities reflect the efficacy of the procedure, with procedural modifications potentially acting as confounders that should be taken into consideration. Supplementary Fig. C shows no significant differences in UDVA (0.00 logMAR, 95% CI: −0.01 to 0.01 logMAR, *z* = 0.71, *p* = 0.48) between Eyhance and monofocal lenses, which may be attributed to the lack of differences in postoperative spherical equivalent (−0.01 D, 95% CI: −0.08 to 0.07 D, *z* = −0.51, *p* = 0.81), with a prediction interval ranging from −0.34 to 0.33 D. For binocular UIVA and UNVA, differences favoring Eyhance were observed, with −0.14 logMAR for UIVA and −0.15 logMAR for UNVA when subgroup analyses were conducted based on classification type (see Supplementary Figs. D and E, respectively).

The binocular outcomes followed a similar trend to the monocular outcomes reported earlier but with a higher prevalence of serious or critical risk of bias (see Supplementary File C). In contrast to studies reporting monocular visual acuities, the use of non-standard measurement methods led to an underestimation of pooled differences for UIVA (−0.12, CI: −0.16 to −0.07 logMAR) compared to standard methods (−0.16, CI: −0.21 to −0.11 logMAR). However, this underestimation was not observed for UNVA. A slight underestimation due to a higher risk of bias was observed for UIVA (−0.13, CI: −0.18 to −0.08 logMAR) in studies with higher bias, compared to those with a lower risk of bias (−0.16, CI: −0.20 to −0.10 logMAR). Similarly, for UNVA, the underestimation was −0.13 (CI: −0.22 to −0.05 logMAR) in higher bias studies compared to −0.17 (CI: −0.25 to −0.09 logMAR) in studies with lower bias. No other significant confounders were identified, including age, postoperative spherical equivalent, or corneal astigmatism inclusion criteria.

#### Binocular defocus curves

Supplementary Fig. F presents the forest plot of the subgroup analysis based on the defocus lens in the binocular defocus curve. All studies assessed the binocular defocus curve with the best distance correction, except for Choi [26], who reported the uncorrected distance defocus curve, whereas Lopes [27], Elbakry [28], and Unsal [29] did not clearly specify this in their manuscripts. The analysis focused on the defocus range from 0 to −2.00 D, with an assumed standard deviation of 0.1 logMAR in up to 12 comparisons.

Statistically significant differences between Eyhance and monofocal IOLs were observed from −0.5 D to −2.00 D, with the differences increasing slightly as defocus increased. Clinically relevant differences were noted at defocus levels beyond −1.0 D. No statistically significant differences were reported in four studies: Mencucci ([18]a, Vivinex Impress; [18]b, IsoPure), Corbelli ([23], Zoe Primus-HD), Elbakry ([28], ZCB00) and Micheletti [30], (Clareon CCA0T0 or CNA0T0). These outcomes were consistent with the measurements of proximal visual acuity in the first two studies, but not for Micheletti and Elbakry which reported significant differences in favor of Eyhance for binocular DCIVA (−0.05 logMAR, *p* < 0.001) and binocular UIVA (−0.39 logMAR, *p* < 0.001), respectively. Additionally, both studies were rated as having a critical risk of bias (see Supplementary File C). In the selection domain for Micheletti, there was an inherent bias due to the inclusion of patients with astigmatism requiring toric IOLs. However, these lenses were exclusively utilized in the Eyhance group. Additionally, there was a measurement and reporting bias, as discrepancies exist between the manuscript and the protocol regarding the method of measuring visual acuity, specifically with respect to the corrected visual acuity for a targeted refractive error of −0.25 D for Clareon. Whereas critical risk of bias for Elbakry was attributed to confounding, as well as to the methods used for measuring and reporting outcomes. Patients were targeted non-uniformly across groups, which likely led to underestimations of UIVA. A subgroup analysis excluding these studies did not result in a significant change in the overall mean differences, which shifted slightly from −0.05 logMAR (CI: −0.06 to −0.04; PI: −0.15 to 0.04) to −0.06 logMAR (CI: −0.07 to −0.05; PI: −0.15 to 0.03).

#### Binocular contrast sensitivity function

Only four studies reported binocular CSF comparing Eyhance with five monofocal IOLs. However, three of these five comparisons (Mencucci [18]a, Vivinex Impress; [18]b, IsoPure; Corbelli [23], Zoe Primus-HD) showed no differences in intermediate visual acuities, as discussed in previous sections. Therefore, only two studies remained relevant: Steinmüller [31], which reported with best distance correction, and Corbelli [32], which did not specify. The differences between groups were less than 0.1 logCS across all spatial frequencies, lacking clinical relevance.

#### Spectacle independence

**Fig. 7:**
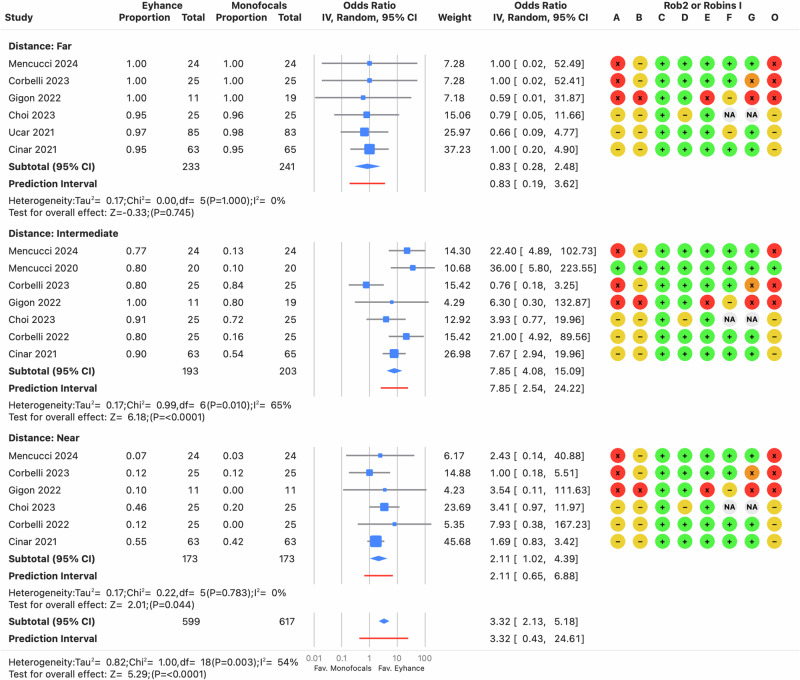
Forest plot of spectacle independence at far, intermediate, and near distances. Odds ratio from individual studies (blue squares) are displayed in rows, with confidence intervals (CI) represented by horizontal lines. The pooled mean difference from all studies is shown as a diamond, extending to the pooled CI. A red line indicates the prediction interval. Colored circles next to each study represent the risk of bias for each domain (A to G, based on RoB 2 or ROBINS-I) and the overall bias (O). Meta-analysis statistics are summarized at the bottom left.

Six or seven comparisons across different distances were pooled to evaluate spectacle independence, with studies ranging from serious to critical risk, as well as low to moderate risk (see Supplementary File C). No differences between Eyhance and other monofocals were found for far distance 0.83 (95% CI: 0.28–2.48) (see Fig. 7). Conversely, Eyhance significantly increased the odds of achieving spectacle independence compared to other monofocals for intermediate distance, with an odds ratio of 7.85 (95% CI: 4.08–15.09) (see Fig. 7). The smallest effect was observed in the study by Corbelli [23], (Zoe Primus-HD). After excluding this study, the odds ratio increased to 11.5 (95% CI: 6.13–20.94), indicating a greater increase in spectacle independence. For near spectacle independence, the odds were also significantly higher in favor of Eyhance after this sub-analysis, though the effect size was considerably lower (OR: 2.16, 95% CI: 1.21–3.85).

#### Photic phenomena and dysphotopsia

**Fig. 8:**
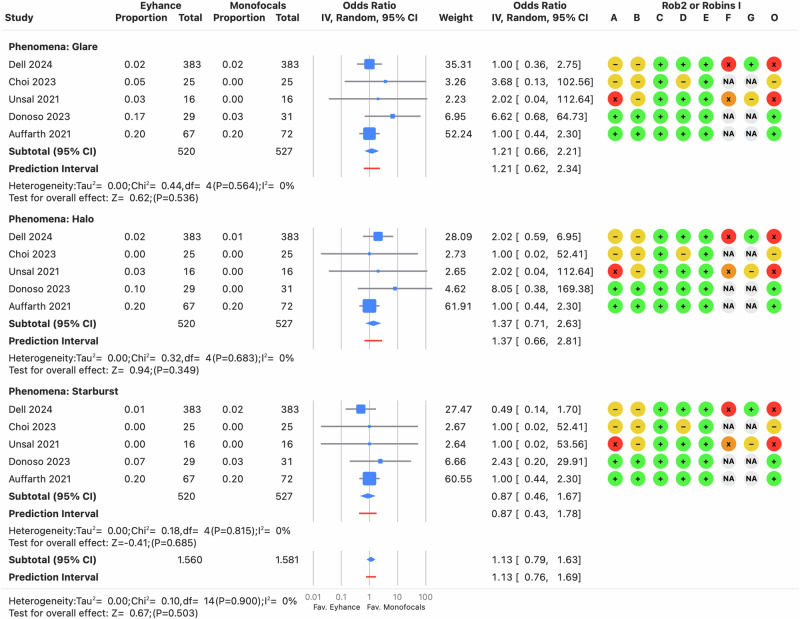
Forest plot of positive dysphotopsia for halo, glare, and starburst. Odds ratio from individual studies (blue squares) are displayed in rows, with confidence intervals (CI) represented by horizontal lines. The pooled mean difference from all studies is shown as a diamond, extending to the pooled CI. A red line indicates the prediction interval. Colored circles next to each study represent the risk of bias for each domain (A to G, based on RoB 2 or ROBINS-I) and the overall bias (O). Meta-analysis statistics are summarized at the bottom left.

The odds ratio for an increased likelihood of experiencing frequent or very frequent photic phenomena (halo, glare, or starburst) was not elevated by Eyhance. The overall odds ratio was 0.62 (CI: 0.32–1.20; PI: 0.30–1.28) (see Supplementary Fig. G). Similarly, the odds ratio for an increased likelihood of being bothered or very bothered by these phenomena was comparable between the groups, with a value of 1.13 (CI: 0.79–1.63; PI: 0.76–1.69) (see Fig. 8). As with other outcomes measured binocularly without distance correction, the risk of bias was higher compared to outcomes assessed monocularly with distance correction (see Supplementary File C).

#### Satisfaction and undergoing the same intervention

Satisfaction after the procedure was only reported by three studies: Dell [33], Lopes [27], and Donoso [22]. In both groups, more than 90% of patients were satisfied or very satisfied, with no clinically relevant differences observed (≤3%). Additionally, only Lopes [27] reported 0% dissatisfied or very dissatisfied patients, while Dell [33] was the only study to report the likelihood of recommending or undergoing the same procedure, again showing no clinically relevant differences (equal to 3%). Due to the poor reporting frequency and inconsistent data across studies, it was not possible to pool the outcomes for this analysis.

### Small-study effect evaluation

Funnel plots were inspected to investigate potential small-study biases for visual acuity and defocus curve measurements, as ten or more studies were pooled for these variables. The number of studies missing to the left or right of the mean was ≤2 for all variables, with minimal impact on the outcomes (<0.01 logMAR). Begg’s rank correlation was tested instead of Egger’s test, as the latter can have low power and assumes a linear relationship between standard error and effect size, which might not always hold. Begg’s rank correlation was significant for intermediate and near visual acuity measurements, either monocular with distance correction or binocular without correction (*p* < 0.05), indicating a larger effect in studies with smaller sample sizes.

In total, fourteen clinical trials were identified, of which five were published and included in the analysis. Four trials are ongoing or recently completed, while four others have remained unpublished for more than a year since their estimated completion date (Supplementary File D). One randomized trial (NCT05025345, comparator ZCB00) reported outcomes on the registration page, showing a difference of −0.11 logMAR in favor of Eyhance for DCIVA. The delay in the publication of the four studies raises concerns about selective outcome reporting and non-reporting. However, the available evidence suggests that these potential biases have had little to no impact on the overall conclusions of the meta-analysis.

### Certainty of evidence

Evidence was graded for the primary and secondary meta-analyzed outcomes, with downgrades in the certainty of evidence due to imprecision, risk of bias, and indirectness where applicable (see Table 2). For distance visual acuity, Eyhance showed no significant differences compared to monofocal IOLs. However, for monocular vision with the best distance correction, Eyhance improved by one line of visual acuity at intermediate and near distances, based on a chart with five letters per row. For binocular vision without distance correction, Eyhance improved by one-and a-half lines at both intermediate and near distances, with high-certainty evidence supporting these findings, except for monocular DCNVA, where moderate-certainty evidence was observed (downgraded due to inconsistency).

**Table 2:** GRADE evidence table for Eyhance versus monofocal intraocular lenses.

People: Patients submitted to cataract surgery Settings: Studies conducted in hospitals across different countries and regions Intervention: Eyhance Comparison: Monofocals				
Outcomes	Mean difference or odds ratio	Number of studies	Certainty of the evidence (GRADE)^a^	Comments
Monocular CDVA (logMAR)	Mean of 0.00 logMAR Confidence interval from −0.01 to 0.01 logMAR Prediction interval from −0.02 to 0.03 logMAR	9 RCT 18 No-RCT	⊕⊕⊕⊕ High	The risk of bias was moderate or low in more than 70% of the studies. Prediction intervals did not show inconsistency, and confidence intervals demonstrated no imprecision. Although three comparisons may involve enhanced intraocular lenses, their relative weight is very low, given the number of pooled studies, including nine randomized controlled trials.
Monocular DCIVA (logMAR)	Mean of −0.11 logMAR Confidence interval from −0.13 to −0.10 logMAR Prediction interval from −0.16 to −0.06 logMAR	7 RCT 12 No-RCT	⊕⊕⊕⊕ High	The risk of bias was moderate or low in more than 70% of the studies. Prediction intervals did not show inconsistency, and confidence intervals demonstrated no imprecision. Sensitivity analysis outcomes, excluding three comparisons that may involve enhanced monofocal intraocular lenses, were also consistent.
Monocular DCNVA (logMAR)	Mean of −0.12 logMAR Confidence interval from −0.17 to −0.07 logMAR Prediction interval from −0.27 to 0.03 logMAR	2 RCT 11 No-RCT	⊕⊕⊕⊖ Moderate	The risk of bias was moderate or low in more than 70% of the studies. The confidence interval did not show imprecision, but the prediction interval indicated some inconsistency, which was partially identified in the sensitivity analysis, leading to a downgrade. Sensitivity analysis outcomes, excluding three comparisons that might involve enhanced monofocal intraocular lenses, were also assessed.
Monocular DC (logMAR) at −1.5 D of Defocus	Mean of −0.1 logMAR Confidence interval from −0.13 to −0.07 logMAR Prediction interval from -0.19 to −0.02 logMAR	2 RCT 8 No-RCT	⊕⊕⊕⊖ Moderate	The risk of bias was serious or critical in more than 50% of the studies, leading to a downgrade. The confidence interval did not show imprecision, but the prediction interval indicated inconsistency, resulting in another downgrade. This inconsistency is partially explained by the inclusion of populations with comorbidities, which may reduce the effect, leading to an upgrade.
Monocular CSF (logCS) at 18 cpd	Mean of −0.03 logCS Confidence interval from −0.10 to 0.05 logCS Prediction interval from −0.18 to 0.13 logCS	2 RCT 3 No-RCT	⊕⊕⊖⊖ Low	The risk of bias was moderate or low in more than 70% of the studies. The confidence interval did not show imprecision, but the prediction interval indicated inconsistency, leading to a downgrade. This inconsistency is partially explained by the inclusion of populations with comorbidities, which may reduce the effect; however, the very low number of pooled studies also contributed to the downgrade.
Binocular UDVA	Mean of 0.00 logMAR Confidence interval from −0.01 to 0.01 logMAR Prediction interval from −0.02 to 0.03 logMAR	7 RCT 10 No-RCT	⊕⊕⊕⊕ High	The risk of bias was serious or critical in more than 50% of the studies, but this was only observed in the case series. Prediction and confidence intervals did not show inconsistency or imprecision, respectively, despite the inclusion of populations with comorbidities.
Binocular UIVA	Mean of −0.14 logMAR Confidence interval from −0.18 to −0.11 logMAR Prediction interval from −0.29 to 0.01 logMAR	7 RCT 14 No-RCT	⊕⊕⊕⊕ High	The risk of bias was serious or critical in more than 50% of the studies, but this was only observed in case series. The confidence interval did not show imprecision, but the prediction interval indicated inconsistency, leading to a downgrade. The inclusion of variation in the procedures may explain the increase in bias, resulting in an upgrade.
Binocular UNVA	Mean of −0.15 logMAR Confidence interval from −0.19 to −0.10 logMAR Prediction interval from −0.29 to −0.01 logMAR	2 RCT 10 N-RCT	⊕⊕⊕⊕ High	The risk of bias was serious or critical in more than 50% of the studies, but this was only observed in the case series. The confidence interval did not show imprecision, but the prediction interval indicated inconsistency, leading to a downgrade. The inclusion of variation in the procedures may explain the increase in bias, resulting in an upgrade.
Binocular DC at −1.5 D of Defocus	Mean of −0.08 logMAR Confidence interval from −0.11 to −0.06 logMAR Prediction interval from −0.16 to −0.01 logMAR	7 RCT 11 No-RCT	⊕⊕⊕⊖ Moderate	The risk of bias was serious or critical in more than 50% of the studies, but this was only observed in the case series. The confidence interval did not show imprecision, but the prediction interval indicated inconsistency, leading to a downgrade. The inclusion of variation in the procedures cannot explain the increase in bias, as the majority of defocus curves were obtained with correction at distance.
Spectacle Independence at Far Distance	Odd Ratio of 0.83 Confidence interval from 0.28 to 2.48 Prediction interval from 0.19 to 3.62	1 RCT 5 No-RCT	⊕⊕⊕⊖ Moderate	The risk of bias was serious or critical in more than 50% of the studies, but this was only observed in the case series. Prediction and confidence intervals did not show inconsistency or imprecision. However, the analysis included only a few studies, with only one randomized controlled trial, leading to a downgrade.
Spectacle Independence at Intermediate Distance	Odd Ratio of 7.85 Confidence interval from 4.08 to 15.09 Prediction interval from 2.54 to 24.22	1 RCT 6 No-RCT	⊕⊕⊖⊖ Low	The risk of bias was serious or critical in more than 50% of the studies, but this was only observed in the case series. Both prediction and confidence intervals showed inconsistency and imprecision, leading to a downgrade. The inclusion of populations with comorbidities may increase bias, but this effect is limited to case series. Additionally, the analysis included only a few studies, with just one randomized controlled trial, resulting in another downgrade.
Spectacle Independence at Intermediate Near	Odd Ratio of 2.11 Confidence interval from 1.02 to 4.39 Prediction interval from 0.65 to 6.88	1 RCT 5 No-RCT	⊕⊕⊕⊖ Moderate	The risk of bias was serious or critical in more than 50% of the studies, but this was only observed in the case series. Prediction and confidence intervals did not show inconsistency or imprecision. However, the analysis included only a few studies, with just one randomized controlled trial, leading to a downgrade.
Photic Phenomena	Odd Ratio of 0.63 Confidence interval from 0.33 to 1.21 Prediction interval from 0.32 to 1.27	1 RCT 7 No-RCT	⊕⊕⊖⊖ Low	The risk of bias was serious or critical in more than 50% of the studies, leading to a downgrade. Prediction and confidence intervals did not show inconsistency or imprecision. Variations in the included procedures may increase bias in non-randomized controlled trials. Additionally, there was only one randomized controlled trial for Halo and Glare, and none for Starburst, resulting in another downgrade.
Positive Dysphotopsia	Odd Ratio of 1.13 Confidence interval from 0.79 to 1.63 Prediction interval from 0.76 to 1.69	3 RCT 2 No-RCT	⊕⊕⊕⊖ Moderate	The risk of bias was serious or critical in more than 50% of the studies, but this was only observed in the case series. Prediction and confidence intervals did not show inconsistency or imprecision. Variations in the included procedures may increase bias in non-randomized controlled trials. Additionally, the analysis included only a few studies, leading to a downgrade.

^a^GRADE Working Group grades of evidence.

**High** = This research provides a very good indication of the likely effect. The likelihood that the effect will be substantially different^b^ is low.

**Moderate** = This research provides a good indication of the likely effect. The likelihood that the effect will be substantially different^b^ is moderate.

**Low** = This research provides some indication of the likely effect. However, the likelihood that it will be substantially different^b^ is high.

**Very low** = This research does not provide a reliable indication of the likely effect. The likelihood that the effect will be substantially different^b^ is very high.

^b^Substantially different = a large enough difference that it might affect a decision.

Monocular and binocular defocus curves showed differences between the comparison groups, though the effect was smaller than that observed for visual acuity at proximal distances. These results were supported by moderate-certainty evidence, downgraded due to the risk of bias and inconsistency.

Certain endpoints, such as monocular contrast sensitivity function and photic phenomena, showed no significant differences between groups, supported by low-certainty evidence. For spectacle independence at far and near distances, moderate-certainty evidence indicated no differences between groups. However, spectacle independence at intermediate distances showed an increased odds ratio, though this result was based on low-certainty evidence. Finally, no differences were noted for positive dysphotopsia, with moderate-certainty evidence supporting this finding.

## Discussion

This meta-analysis included 31 studies, examining the effectiveness of the Eyhance IOL compared to other monofocals across several visual outcomes. The findings consistently showed that Eyhance improved intermediate and near monocular and binocular visual acuities by one to one-and-a-half lines, as measured on a visual acuity chart with five letters per row. Notably, while spectacle independence at an intermediate distance was significantly improved with Eyhance, no difference was observed at a far distance. At a near distance, although the difference reached statistical significance, it was smaller, and the lower range of the confidence interval approached the threshold for no difference, indicating that further studies are necessary to increase certainty regarding near spectacle independence. In contrast, far-distance contrast sensitivity, photic phenomena, and positive dysphotopsia outcomes did not significantly differ between Eyhance and comparator lenses. Subgroup analyses revealed that heterogeneity among studies was partly driven by differences in comparator lenses and testing conditions, with some monofocal IOLs that might offer similar outcomes to Eyhance (Vivinex Impress; IsoPure and Zoe Primus-HD) but with only one non-randomized study per lens and graded by a moderately or high risk of bias according to ROBINS-I tool.

Our findings align with early meta-analyses on Eyhance IOL, which have demonstrated improved intermediate and near vision without sacrificing distance visual acuity [5, 6]. However, our meta-analysis addresses several methodological limitations present in the previous literature. For example, the risk of bias should be assessed at the outcome level, as this can vary depending on the endpoint assessed, but prior meta-analyses evaluated bias at the study level [34]. Additionally, the certainty of the evidence has not been evaluated in previous meta-analyses [5, 6]. Considering these methodological limitations and the inclusion of 31 studies compared to the 12 analyzed previously, our work not only confirms earlier evidence but also introduces a more robust methodological approach that has not been adequately emphasized in previous meta-analyses. Additionally, small-study effects were identified in monocular and binocular intermediate and near visual acuity measurements, indicating higher effects in studies with smaller sample sizes.

In addition, the population pool evaluated in this meta-analysis is broader than what has been addressed in previous literature. This likely explains the high heterogeneity found in several endpoints. Although some researchers believe that heterogeneity diminishes the utility of an analysis, this is a misconception [35]. Heterogeneity simply indicates that the true effect size varies across studies. In our analyses, data were extracted on several potential confounders, including IOLs that might be classified similarly to Eyhance as PARTIAL-RoF-Enhanced IOLs [2], patients with comorbidities, and variations in procedures. This provides a more comprehensive view of the intervention’s effectiveness in these specific situations. Furthermore, the inclusion of comparative case series, which often carry a higher risk of bias, offers evidence that aligns with real-world clinical practice, where both the intervention and comparator are utilized.

Several limitations of the current evidence must be acknowledged. First, the risk of bias was generally higher in non-randomized studies, with issues such as confounding, selection bias, and deviations from intended interventions frequently observed. Failure to use standardized methods for measuring visual outcomes and inadequate reporting of postoperative conditions (e.g., residual refractive errors) in several studies contributed to the heterogeneity in the results and limited our ability to draw firm conclusions for some outcomes, particularly contrast sensitivity and patient-reported outcomes.

Additionally, while the meta-analysis incorporated data from 31 studies, the evidence for certain secondary outcomes, such as binocular contrast sensitivity and patient-reported satisfaction, was sparse. Only a few studies addressed these outcomes, and many were at risk of bias. Another limitation arises from the exclusion of unpublished trials or those with incomplete data, which raises the possibility of publication bias, particularly for ongoing trials that have yet to report their outcomes. Finally, the assumption of standard deviations for defocus curves and contrast sensitivity in studies lacking reported data may have introduced imprecision into the pooled estimates, although sensitivity analyses suggest this had minimal impact on the overall conclusions.

The results of this meta-analysis suggest that Eyhance IOL offers a clinically relevant improvement in intermediate and near vision compared to conventional monofocal IOLs. However, moderate evidence suggests comparable spectacle independence at near, which means that the improvement of visual acuity at near might not be as high enough as to decrease the spectacle independence, and therefore cost-effectiveness for the intervention in near tasks could be questionable.

While no significant differences in far-distance contrast sensitivity, photic phenomena, and positive dysphotopsia were found, there remains a low certainty in some of these variables, and future research should aim to improve the quality and consistency of reporting. This includes standardizing outcome measurements and ensuring the adequate reporting of postoperative conditions and adverse events [36]. Furthermore, additional studies are needed to investigate the performance of Eyhance IOLs in comparison to other PARTIAL-RoF-Enhanced IOLs, as well as to confirm their effectiveness in specific subpopulations, including patients with ocular comorbidities. Future and ongoing trials should prioritize robust design with appropriate randomization, masking, and reporting, especially considering the high risk of bias identified in several non-randomized studies.

## Conclusion

The findings of this meta-analysis provide high-certainty evidence that the Eyhance IOL significantly improves intermediate and near visual acuity, with patients experiencing an increase of one to one-and-a-half lines in visual acuity compared to conventional monofocal IOLs depending on the monocular or binocular testing conditions. However, the evidence for other outcomes, such as contrast sensitivity and patient-reported outcomes, remains moderate or low. This underscores the need for adherence to standardized data collection and reporting methods to strengthen the evidence base. Additionally, newer IOLs, such as Vivinex Impress, IsoPure, and Zoe Primus-HD, may offer similar visual outcomes to Eyhance, but few comparative studies exist, many of which are graded with a high risk of bias. Future research on new PARTIAL-RoF-Enhanced IOLs should aim to confirm these findings and establish their superiority over traditional monofocal IOLs while demonstrating non-inferiority to Eyhance.

## Summary

### What is known

- Enhanced monofocal intraocular lenses improve intermediate visual acuity compared to standard monofocals, while maintaining similar distance vision.
- Further research was needed to clarify the effectiveness of enhanced monofocals at near distances and patient-reported outcomes.

### New Information

- This paper increases reliability by grading evidence certainty and assessing bias at the outcome level, offering more precise and trustworthy conclusions on enhanced IOL outcomes.
- There is high-certainty evidence that enhanced IOLs improve intermediate and near visual acuity over conventional monofocal IOLs, with moderate to low certainty for benefits in defocus curves, contrast sensitivity, and patient-reported outcomes such as spectacle independence and photic phenomena.

## Supplementary information

Supplementary Fig. A: Forest Plot of Subgroup Analysis by Author-Attributed IOL Functional Classification for DCIVA Outcome

Supplementary Fig. B: Forest Plot of Subgroup Analysis by Author-Attributed IOL Functional Classification for DCNVA Outcome

Supplementary Fig. C: Forest Plot of UDVA Outcome

Supplementary Fig. D: Forest Plot of Subgroup Analysis by Author-Attributed IOL Functional Classification for UIVA Outcome

Supplementary Fig. E: Forest Plot of Subgroup Analysis by Author-Attributed IOL Functional Classification for UNVA Outcome

Supplementary Fig. F: Forest Plot of Subgroup Analysis by DC Outcome

Supplementary Fig. G: Forest Plot of Subgroup Analysis by Type for PP Outcome

Supplementary File A: Search strategy

Supplementary File B: Risk of Bias Assessment with Robins-I or Rob2

Supplementary File C: Risk of Bias Assessment Plots

Supplementary File D: Registered studies
