# Supplementary material for: Visual and patient-reported outcomes of an enhanced versus monofocal intraocular lenses in cataract surgery: a systematic review and meta-analysis
Source: Eye (Lond). 2025 Feb 1;39(5):883–98. doi: 10.1038/s41433-025-03625-4 (PMC11933469; doi:10.1038/s41433-025-03625-4)

Supplemental Figure A: Forest Plot of Subgroup Analysis by Author-Attributed IOL Functional Classification for DCIVA Outcome

Study	Eyhance Mean	Eyhance SD	Eyhance Total	Monofocals Mean	Monofocals SD	Monofocals Total	Weight	Mean Difference IV, Random, 95% CI	A	B	C	D	E	F	G	O
**Classification: Enhanced**																
Mencucci 2023a	0.23	0.07	24	0.22	0.04	24	34.60	0.01 [-0.02, 0.04]	x	+	+	+	+	+	+	x
Mencucci 2023b	0.23	0.07	24	0.21	0.09	24	25.37	0.02 [-0.03, 0.07]	x	+	+	+	+	+	+	x
Corbelli 2023	0.28	0.07	50	0.29	0.06	50	40.03	-0.01 [-0.04, 0.02]	+	−	+	+	+	+	+	−
**Subtotal (95% CI)**			98			98		0.00 [-0.03, 0.04]								
**Prediction Interval**								0.00 [-0.05, 0.06]								
Heterogeneity:Tau²= 0.000;Chi²= 0.57,df= 2(P=0.433); I²= 0%																
Test for overall effect: Z= 0.28;(P=0.778)																
**Classification: Narrowed**																
Mencucci 2024	0.29	0.09	24	0.37	0.09	24	6.59	-0.08 [-0.13, -0.03]	x	−	+	+	+	+	+	x
Mencucci 2020	0.27	0.11	40	0.37	0.09	40	7.74	-0.10 [-0.14, -0.06]	+	+	+	+	+	+	+	+
Kozhaya 2024	0.30	0.13	11	0.24	0.16	11	1.75	0.06 [-0.06, 0.18]	x	x	x	+	+	+	+	x
Steinmüller 2022	0.08	0.11	28	0.33	0.16	26	4.09	-0.25 [-0.32, -0.18]	+	+	+	+	+	+	+	+
Gigon 2022	0.30	0.10	20	0.40	0.10	38	6.12	-0.10 [-0.15, -0.05]	−	x	+	+	x	−	x	x
Corbelli 2022	0.25	0.07	50	0.36	0.05	50	12.18	-0.11 [-0.13, -0.09]	−	−	+	+	+	+	+	−
Ucar 2021	0.07	0.06	85	0.17	0.05	83	13.86	-0.10 [-0.12, -0.08]	−	−	+	+	+	−	+	−
Cinar 2021	0.28	0.12	63	0.38	0.13	65	7.86	-0.10 [-0.14, -0.06]	−	−	+	+	+	+	+	−
Unsal 2021	0.28	0.31	32	0.43	0.46	32	0.75	-0.15 [-0.34, 0.04]	x	−	+	+	+	x	−	x
Eguileor 2020	0.26	0.13	30	0.45	0.12	30	4.99	-0.19 [-0.25, -0.13]	−	−	+	−	+	NA	NA	−
Giglio 2024a	0.17	0.13	30	0.29	0.11	30	5.26	-0.12 [-0.18, -0.06]	−	−	+	−	+	NA	NA	−
Giglio 2024b	0.17	0.13	30	0.31	0.11	30	5.26	-0.14 [-0.20, -0.08]	−	−	+	−	+	NA	NA	−
Goslings 2023	0.23	0.13	70	0.33	0.13	70	7.92	-0.10 [-0.14, -0.06]	−	−	+	−	+	NA	NA	−
Donoso 2023	0.20	0.15	29	0.30	0.16	31	3.64	-0.10 [-0.18, -0.02]	+	+	+	+	+	NA	NA	+
Nanavaty 2022	0.27	0.17	50	0.37	0.10	50	6.05	-0.10 [-0.15, -0.05]	−	x	+	−	x	NA	NA	x
Auffarth 2021	0.19	0.23	134	0.31	0.24	144	5.96	-0.12 [-0.18, -0.06]	+	+	+	+	+	NA	NA	+
**Subtotal (95% CI)**			726			754		-0.11 [-0.13, -0.10]								
**Prediction Interval**								-0.11 [-0.16, -0.06]								
Heterogeneity:Tau²= 0.000;Chi²= 0.99,df= 15(P=0.005); I²= 54%																
Test for overall effect: Z=-12.93;(P=<0.0001)																
**Total (95% CI)**			824			852		-0.09 [-0.10, -0.07]								
**Prediction Interval**								-0.09 [-0.21, 0.03]								

Heterogeneity:Tau²= 0.003;Chi²= 1.00,df= 18(P=<0.0001)
Test for overall effect: Z=-11.23;(P=<0.0001)

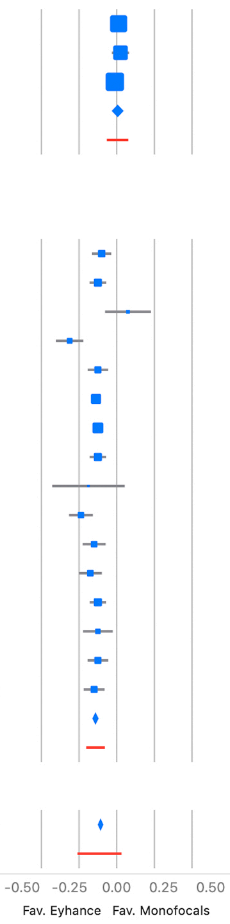

Supplement: Supplementary file 1 — Supplementary Fig. A: Forest Plot of Subgroup Analysis by Author-Attributed IOL Functional Classification for DCIVA Outcome [file 41433_2025_3625_MOESM1_ESM.pdf]
