# Supplementary material for: Visual and patient-reported outcomes of an enhanced versus monofocal intraocular lenses in cataract surgery: a systematic review and meta-analysis
Source: Eye (Lond). 2025 Feb 1;39(5):883–98. doi: 10.1038/s41433-025-03625-4 (PMC11933469; doi:10.1038/s41433-025-03625-4)

Supplemental Figure B: Forest Plot of Subgroup Analysis by Author-Attributed IOL Functional Classification for DCNVA Outcome

Study	Eyhance Mean	Eyhance SD	Eyhance Total	Monofocals Mean	Monofocals SD	Monofocals Total	Weight	Mean Difference IV, Random, 95% CI	A	B	C	D	E	F	G	O
**Classification: Enhanced**																
Mencucci 2023a	0.46	0.12	24	0.43	0.07	24	31.92	0.03 [-0.03, 0.09]	x	+	+	+	+	+	+	x
Mencucci 2023b	0.46	0.12	24	0.41	0.09	24	31.06	0.05 [-0.01, 0.11]	x	+	+	+	+	+	+	x
Corbelli 2023	0.33	0.06	50	0.34	0.06	50	37.01	-0.01 [-0.03, 0.01]	+	−	+	+	+	+	x	x
**Subtotal (95% CI)**			98			98		0.02 [-0.06, 0.10]								
**Prediction Interval**								0.02 [-0.14, 0.19]								
Heterogeneity:Tau²= 0.004;Chi²= 0.89,df= 2(P=0.109); I²= 55%																
Test for overall effect: Z= 0.55;(P=0.584)																
**Classification: Narrowed**																
Mencucci 2020	0.44	0.13	40	0.49	0.10	40	12.08	-0.05 [-0.10, 0.00]	+	+	+	+	+	+	+	+
Kozhaya 2024	0.46	0.20	11	0.52	0.15	11	5.83	-0.06 [-0.21, 0.09]	x	x	x	+	+	+	+	x
Steinmüller 2022	0.25	0.21	28	0.55	0.15	26	8.68	-0.30 [-0.40, -0.20]	+	+	+	+	+	+	+	+
Gigon 2022	0.30	0.10	20	0.40	0.10	38	11.85	-0.10 [-0.15, -0.05]	−	x	+	+	x	−	x	x
Corbelli 2022	0.32	0.04	50	0.51	0.09	50	13.46	-0.19 [-0.22, -0.16]	−	−	+	+	+	+	+	−
Ucar 2021	0.44	0.18	85	0.57	0.17	83	11.93	-0.13 [-0.18, -0.08]	−	−	+	+	+	−	+	−
Cinar 2021	0.46	0.17	63	0.46	0.15	65	11.75	0.00 [-0.06, 0.06]	−	−	+	+	+	+	+	−
Unsal 2021	0.55	0.45	32	0.75	0.80	32	1.86	-0.20 [-0.52, 0.12]	x	−	+	+	+	x	−	x
Giglio 2024a	0.25	0.13	30	0.38	0.11	30	11.36	-0.13 [-0.19, -0.07]	−	−	+	−	+	NA	NA	−
Giglio 2024b	0.25	0.13	30	0.36	0.12	30	11.19	-0.11 [-0.17, -0.05]	−	−	+	−	+	NA	NA	−
**Subtotal (95% CI)**			389			405		-0.12 [-0.17, -0.07]								
**Prediction Interval**								-0.12 [-0.27, 0.03]								
Heterogeneity:Tau²= 0.004;Chi²= 1.00,df= 9(P=<0.0001); I²= 86%																
Test for overall effect: Z=-5.02;(P=<0.0001)																
**Total (95% CI)**			487			503		-0.08 [-0.12, -0.04]								
**Prediction Interval**								-0.08 [-0.29, 0.13]								
Heterogeneity:Tau²= 0.008;Chi²= 1.00,df= 12(P=<0.0001)																
Test for overall effect: Z=-4.01;(P=<0.0001)																

(Rob2 or Robins I: columns A–O)

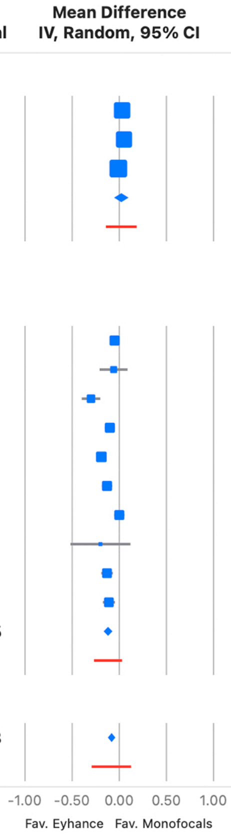

Supplement: Supplementary file 2 — Supplementary Fig. B: Forest Plot of Subgroup Analysis by Author-Attributed IOL Functional Classification for DCNVA Outcome [file 41433_2025_3625_MOESM2_ESM.pdf]
