# Supplementary figures and images for: Visual and patient-reported outcomes of an enhanced versus monofocal intraocular lenses in cataract surgery: a systematic review and meta-analysis
Source: Eye (Lond). 2025 Feb 1;39(5):883–98. doi: 10.1038/s41433-025-03625-4 (PMC11933469; doi:10.1038/s41433-025-03625-4)

Supplemental Figure C: Forest Plot of UDVA Outcome

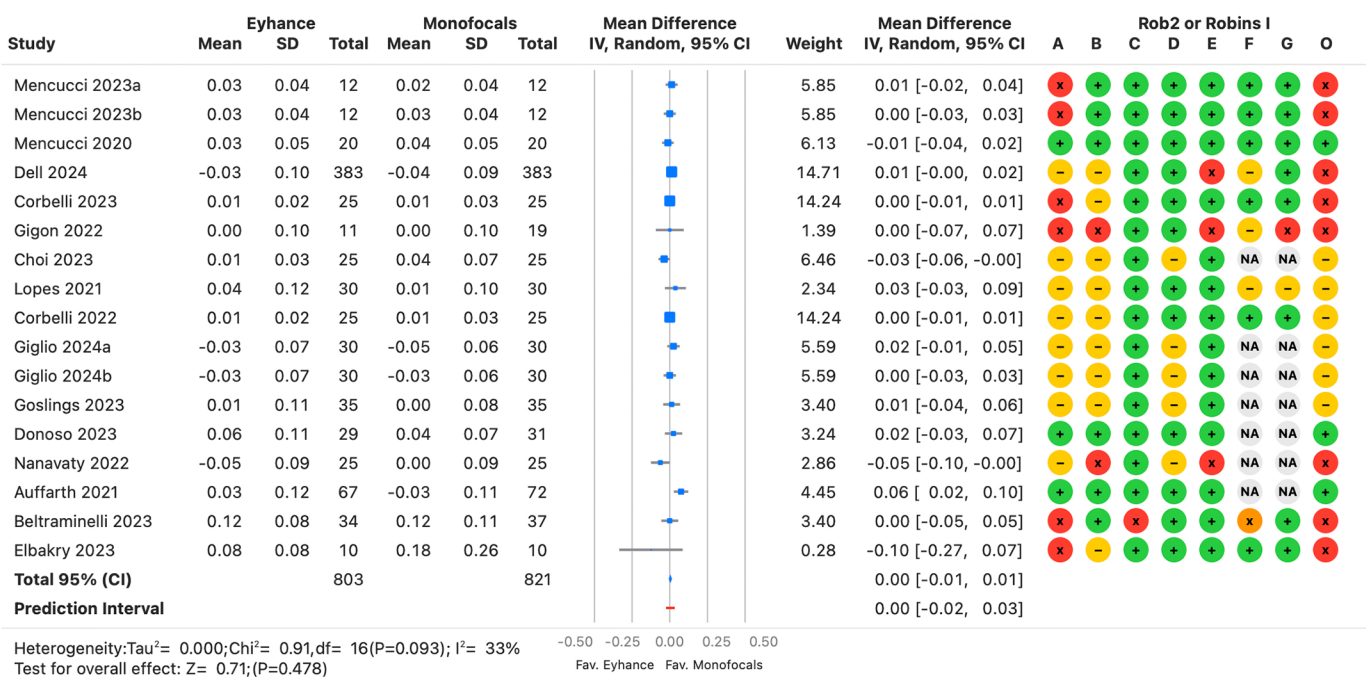

Supplement: Supplementary file 3 — Supplementary Fig. C: Forest Plot of UDVA Outcome [file 41433_2025_3625_MOESM3_ESM.pdf]

Supplemental Figure G: Forest Plot of Subgroup Analysis by Type for PP Outcome

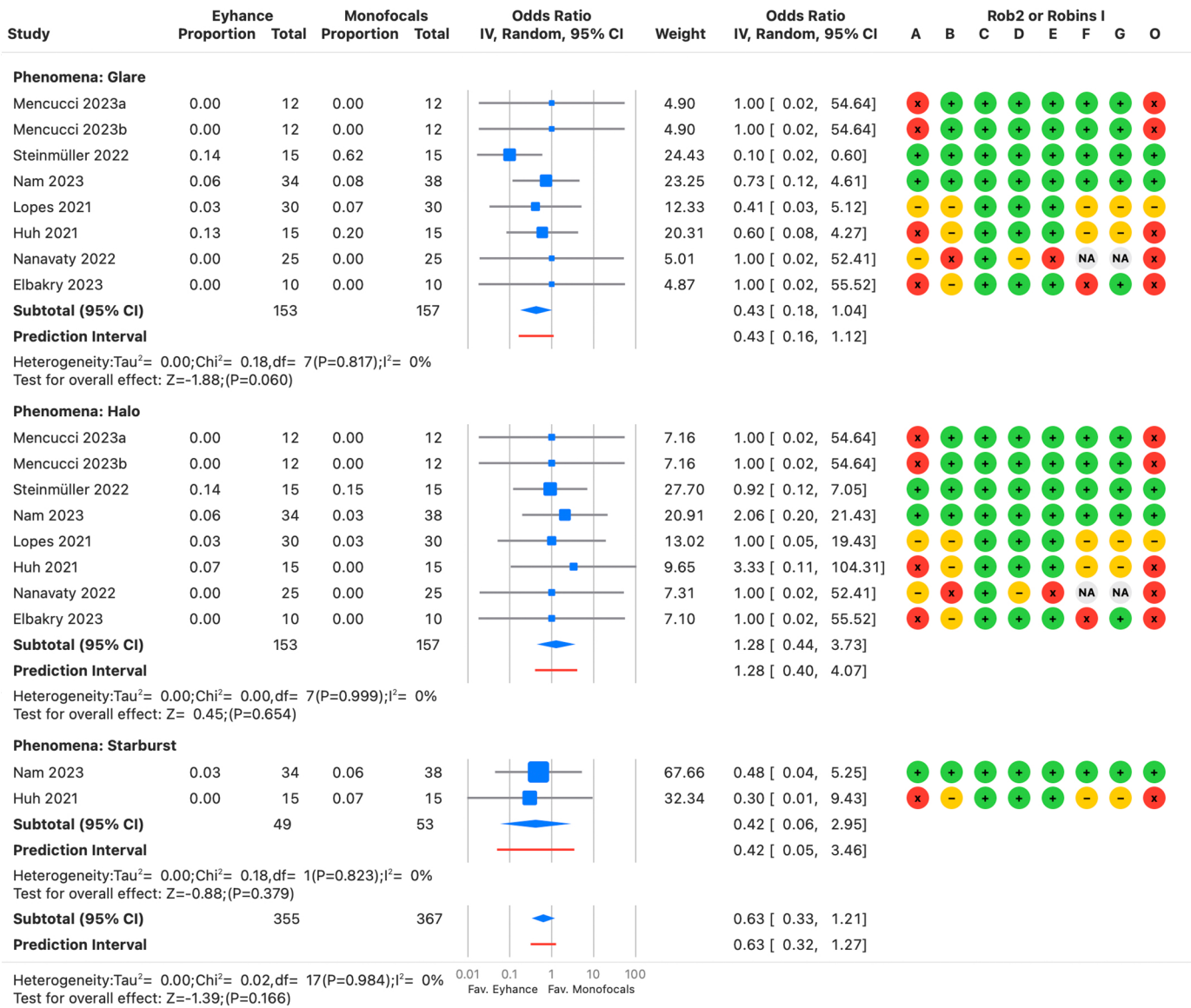

Supplement: Supplementary file 7 — Supplementary Fig. G: Forest Plot of Subgroup Analysis by Type for PP Outcome [file 41433_2025_3625_MOESM7_ESM.pdf]
