# Supplementary material for: Visual and patient-reported outcomes of an enhanced versus monofocal intraocular lenses in cataract surgery: a systematic review and meta-analysis
Source: Eye (Lond). 2025 Feb 1;39(5):883–98. doi: 10.1038/s41433-025-03625-4 (PMC11933469; doi:10.1038/s41433-025-03625-4)

## Supplemental Figure D: Forest Plot of Subgroup Analysis by Author-Attributed IOL Functional Classification for UIVA Outcome

| Study | Eyhance Mean | Eyhance SD | Eyhance Total | Monofocals Mean | Monofocals SD | Monofocals Total | Weight | Mean Difference IV, Random, 95% CI | Rob2 or Robins I A | B | C | D | E | F | G | O |
|---|---|---|---|---|---|---|---|---|---|---|---|---|---|---|---|---|
| **Classification: Enhanced** | | | | | | | | | | | | | | | | |
| Mencucci 2023a | 0.14 | 0.07 | 12 | 0.13 | 0.05 | 12 | 34.61 | 0.01 [-0.04, 0.06] | x | + | + | + | + | + | + | x |
| Mencucci 2023b | 0.14 | 0.07 | 12 | 0.16 | 0.10 | 12 | 30.98 | -0.02 [-0.09, 0.05] | x | + | + | + | + | + | + | x |
| Corbelli 2023 | 0.10 | 0.09 | 25 | 0.09 | 0.09 | 25 | 34.41 | 0.01 [-0.04, 0.06] | x | − | + | + | + | + | + | x |
| **Subtotal (95% CI)** | | | 49 | | | 49 | | 0.00 [-0.08, 0.08] | | | | | | | | |
| **Prediction Interval** | | | | | | | | 0.00 [-0.17, 0.17] | | | | | | | | |
| Heterogeneity:Tau²= 0.005;Chi²= 0.25,df= 2(P=0.749); I²= 0%<br>Test for overall effect: Z= 0.02;(P=0.987) | | | | | | | | | | | | | | | | |
| **Classification: Narrowed** | | | | | | | | | | | | | | | | |
| Giasanti 2023 | 0.02 | 0.06 | 11 | 0.21 | 0.14 | 12 | 4.78 | -0.19 [-0.28, -0.10] | x | + | + | + | − | + | + | x |
| Mencucci 2020 | 0.16 | 0.10 | 20 | 0.27 | 0.06 | 20 | 6.02 | -0.11 [-0.16, -0.06] | + | + | + | + | + | + | + | + |
| Dell 2024 | 0.18 | 0.18 | 383 | 0.26 | 0.20 | 383 | 6.62 | -0.08 [-0.11, -0.05] | − | − | + | + | + | x | + | x |
| Steinmüller 2022 | -0.04 | 0.06 | 15 | 0.07 | 0.17 | 15 | 4.72 | -0.11 [-0.20, -0.02] | + | + | + | + | + | + | + | + |
| Gigon 2022 | 0.20 | 0.10 | 11 | 0.30 | 0.10 | 19 | 5.28 | -0.10 [-0.17, -0.03] | x | x | + | + | x | − | x | x |
| Choi 2023 | 0.04 | 0.05 | 25 | 0.10 | 0.14 | 25 | 5.80 | -0.06 [-0.12, -0.00] | − | − | + | − | + | NA | NA | − |
| Corbelli 2022 | 0.04 | 0.04 | 25 | 0.35 | 0.09 | 25 | 6.36 | -0.31 [-0.35, -0.27] | − | − | + | + | + | + | + | − |
| Lopes 2021 | 0.17 | 0.10 | 30 | 0.30 | 0.13 | 30 | 5.79 | -0.13 [-0.19, -0.07] | − | − | + | + | + | − | − | − |
| Huh 2021 | 0.03 | 0.06 | 15 | 0.25 | 0.18 | 15 | 4.57 | -0.22 [-0.32, -0.12] | x | − | + | + | + | − | − | x |
| Unsal 2021 | 0.18 | 0.10 | 16 | 0.40 | 0.10 | 16 | 5.45 | -0.22 [-0.29, -0.15] | x | − | + | + | + | x | − | x |
| Giglio 2024a | 0.17 | 0.12 | 30 | 0.32 | 0.11 | 30 | 5.80 | -0.15 [-0.21, -0.09] | − | − | + | − | + | NA | NA | − |
| Giglio 2024b | 0.17 | 0.12 | 30 | 0.31 | 0.09 | 30 | 5.94 | -0.14 [-0.19, -0.09] | − | − | + | − | + | NA | NA | − |
| Goslings 2023 | 0.12 | 0.11 | 35 | 0.22 | 0.12 | 35 | 5.94 | -0.10 [-0.15, -0.05] | − | − | + | − | + | NA | NA | − |
| Donoso 2023 | 0.37 | 0.12 | 29 | 0.45 | 0.10 | 31 | 5.88 | -0.08 [-0.14, -0.02] | + | + | + | + | + | NA | NA | + |
| Nanavaty 2022 | 0.13 | 0.10 | 25 | 0.26 | 0.11 | 25 | 5.80 | -0.13 [-0.19, -0.07] | − | x | + | − | x | NA | NA | x |
| Auffarth 2021 | 0.07 | 0.12 | 67 | 0.17 | 0.16 | 72 | 6.13 | -0.10 [-0.15, -0.05] | + | + | + | + | + | NA | NA | + |
| Beltraminelli 2023 | 0.14 | 0.09 | 34 | 0.23 | 0.10 | 37 | 6.21 | -0.09 [-0.13, -0.05] | x | + | x | + | + | x | + | x |
| Elbakry 2023 | 0.36 | 0.18 | 10 | 0.75 | 0.18 | 10 | 2.90 | -0.39 [-0.55, -0.23] | x | − | + | + | + | x | x | x |
| **Subtotal (95% CI)** | | | 811 | | | 830 | | -0.14 [-0.18, -0.11] | | | | | | | | |
| **Prediction Interval** | | | | | | | | -0.14 [-0.29, 0.01] | | | | | | | | |
| Heterogeneity:Tau²= 0.005;Chi²= 1.00,df= 17(P=<0.0001); I²= 87%<br>Test for overall effect: Z=-7.90;(P=<0.0001) | | | | | | | | | | | | | | | | |
| **Total (95% CI)** | | | 860 | | | 879 | | -0.12 [-0.15, -0.09] | | | | | | | | |
| **Prediction Interval** | | | | | | | | -0.12 [-0.30, 0.05] | | | | | | | | |

Heterogeneity:Tau²= 0.007;Chi²= 1.00,df= 20(P=<0.0001)
Test for overall effect: Z=-7.28;(P=<0.0001)

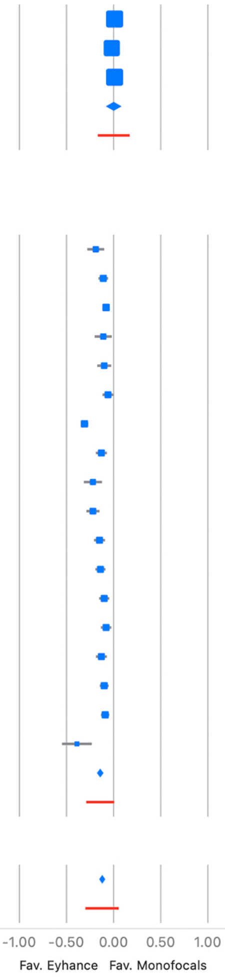

Supplement: Supplementary file 4 — Supplementary Fig. D: Forest Plot of Subgroup Analysis by Author-Attributed IOL Functional Classification for UIVA Outcome [file 41433_2025_3625_MOESM4_ESM.pdf]
