# Supplementary material for: Visual and patient-reported outcomes of an enhanced versus monofocal intraocular lenses in cataract surgery: a systematic review and meta-analysis
Source: Eye (Lond). 2025 Feb 1;39(5):883–98. doi: 10.1038/s41433-025-03625-4 (PMC11933469; doi:10.1038/s41433-025-03625-4)

Supplemental Figure E: Forest Plot of Subgroup Analysis by Author-Attributed IOL Functional Classification for UNVA Outcome

Study	Eyhance Mean	Eyhance SD	Eyhance Total	Monofocals Mean	Monofocals SD	Monofocals Total	Weight	Mean Difference IV, Random, 95% CI	Rob2 or Robins I A	B	C	D	E	F	G	O
**Classification: Enhanced**																
Mencucci 2023a	0.34	0.04	12	0.36	0.07	12	32.08	-0.02 [-0.07, 0.03]	x	+	+	+	+	+	+	x
Mencucci 2023b	0.34	0.04	12	0.35	0.07	12	32.08	-0.01 [-0.06, 0.04]	x	+	+	+	+	+	+	x
Corbelli 2023	0.32	0.04	25	0.32	0.04	25	35.85	0.00 [-0.02, 0.02]	x	−	+	+	+	+	+	x
**Subtotal (95% CI)**			49			49		-0.01 [-0.08, 0.06]								
**Prediction Interval**								-0.01 [-0.16, 0.14]								
Heterogeneity:Tau^2= 0.003;Chi^2= 0.28,df= 2(P=0.720); I^2= 0%																
Test for overall effect: Z=-0.27;(P=0.786)																
**Classification: Narrowed**																
Mencucci 2020	0.33	0.05	20	0.38	0.05	20	13.73	-0.05 [-0.08, -0.02]	+	+	+	+	+	+	+	+
Dell 2024	0.42	0.19	383	0.51	0.22	383	13.84	-0.09 [-0.12, -0.06]	−	−	+	+	+	−	x	x
Steinmüller 2022	0.08	0.10	15	0.28	0.20	15	7.43	-0.20 [-0.31, -0.09]	+	+	+	+	+	+	+	+
Gigon 2022	0.10	0.10	11	0.30	0.10	19	10.35	-0.20 [-0.27, -0.13]	x	x	+	+	x	−	x	x
Choi 2023	0.14	0.13	25	0.29	0.14	25	10.30	-0.15 [-0.22, -0.08]	−	−	+	−	+	NA	NA	−
Corbelli 2022	0.28	0.05	25	0.49	0.06	25	13.75	-0.21 [-0.24, -0.18]	−	−	+	+	+	+	+	−
Huh 2021	0.09	0.14	15	0.35	0.14	15	8.32	-0.26 [-0.36, -0.16]	x	−	+	+	+	−	−	x
Donoso 2023	0.58	0.15	29	0.67	0.09	31	11.37	-0.09 [-0.15, -0.03]	+	+	+	+	+	NA	NA	+
Beltraminelli 2023	0.34	0.16	34	0.50	0.13	37	10.91	-0.16 [-0.23, -0.09]	x	+	x	+	+	x	+	x
**Subtotal (95% CI)**			557			570		-0.15 [-0.19, -0.10]								
**Prediction Interval**								-0.15 [-0.29, -0.01]								
Heterogeneity:Tau^2= 0.003;Chi^2= 1.00,df= 8(P=<0.0001); I^2= 89%																
Test for overall effect: Z=-6.65;(P=<0.0001)																
**Total (95% CI)**			606			619		-0.11 [-0.15, -0.07]								
**Prediction Interval**								-0.11 [-0.31, 0.08]								
Heterogeneity:Tau^2= 0.007;Chi^2= 1.00,df= 11(P=<0.0001)																
Test for overall effect: Z=-5.77;(P=<0.0001)																

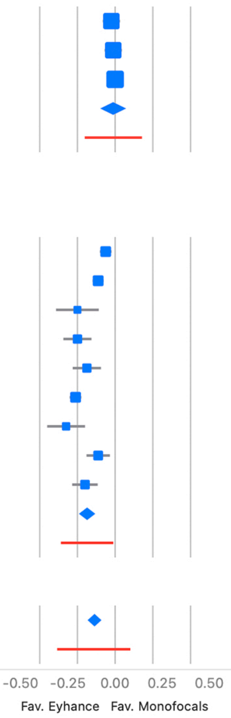

Supplement: Supplementary file 5 — Supplementary Fig. E: Forest Plot of Subgroup Analysis by Author-Attributed IOL Functional Classification for UNVA Outcome [file 41433_2025_3625_MOESM5_ESM.pdf]
