# Supplementary material for: Visual and patient-reported outcomes of an enhanced versus monofocal intraocular lenses in cataract surgery: a systematic review and meta-analysis
Source: Eye (Lond). 2025 Feb 1;39(5):883–98. doi: 10.1038/s41433-025-03625-4 (PMC11933469; doi:10.1038/s41433-025-03625-4)
Supplement: Supplementary file 6 — Supplementary Fig. F: Forest Plot of Subgroup Analysis by DC Outcome [file 41433_2025_3625_MOESM6_ESM.pdf]

Supplemental Figure F: Forest Plot of Subgroup Analysis by Defocus for DC Outcome

Study	Eyhance Mean	Eyhance SD	Eyhance Total	Monofocals Mean	Monofocals SD	Monofocals Total	Weight	Mean Difference IV, Random, 95% CI	A	B	C	D	E	F	G	O
**Defocus: 0.00**																
Mencucci 2023a	0.03	0.10	12	0.02	0.10	12	4.11	0.01 [-0.07, 0.09]	x	+	+	+	+	+	+	x
Mencucci 2023b	0.03	0.10	12	0.03	0.10	12	4.11	0.00 [-0.08, 0.08]	x	+	+	+	+	+	+	x
Mencucci 2020	0.01	0.10	20	0.00	0.10	20	5.30	0.01 [-0.05, 0.07]	+	+	+	+	+	+	+	+
Steinmüller 2022	-0.19	0.09	15	-0.18	0.09	15	5.13	-0.01 [-0.07, 0.05]	+	+	+	+	+	+	+	+
Corbelli 2023	0.02	0.10	25	0.02	0.10	25	5.81	0.00 [-0.06, 0.06]	-	-	+	+	+	+	x	x
Choi 2023	0.00	0.10	25	0.01	0.10	25	5.81	-0.01 [-0.07, 0.05]	-	-	+	-	+	NA	NA	-
Micheletti 2023	0.01	0.02	15	0.01	0.03	15	8.79	0.00 [-0.02, 0.02]	x	x	x	+	-	x	x	x
Lopes 2021	0.01	0.10	30	0.01	0.10	30	6.20	0.00 [-0.05, 0.05]	-	-	+	+	+	-	-	-
Corbelli 2022	0.01	0.03	25	0.02	0.04	25	8.70	-0.01 [-0.03, 0.01]	-	-	+	+	+	+	+	-
Huh 2021	-0.04	0.10	15	-0.05	0.10	15	4.63	0.01 [-0.06, 0.08]	x	-	+	+	+	-	-	x
Unsal 2021	-0.01	0.10	16	0.01	0.10	16	4.78	-0.02 [-0.09, 0.05]	x	-	+	+	+	x	-	x
Eguileor 2020	0.04	0.10	30	0.07	0.10	30	6.20	-0.03 [-0.08, 0.02]	-	-	+	-	+	NA	NA	-
Giglio 2024a	-0.03	0.16	30	-0.05	0.16	30	4.06	0.02 [-0.06, 0.10]	-	-	+	-	+	NA	NA	-
Giglio 2024b	-0.03	0.16	30	-0.01	0.16	30	4.06	-0.02 [-0.10, 0.06]	-	-	+	-	+	NA	NA	-
Donoso 2023	-0.05	0.05	29	0.01	0.00	31	8.83	-0.06 [-0.08, -0.04]	+	+	+	+	+	NA	NA	+
Nanavaty 2022	-0.02	0.10	25	0.00	0.10	25	5.81	-0.02 [-0.08, 0.04]	-	x	+	-	x	NA	NA	x
Auffarth 2021	-0.05	0.10	67	-0.08	0.10	72	7.67	0.03 [-0.00, 0.06]	+	+	+	+	+	NA	NA	+
**Subtotal (95% CI)**			421			428		-0.01 [-0.03, 0.01]								
**Prediction Interval**								-0.01 [-0.08, 0.07]								

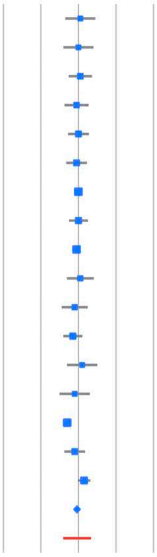

Heterogeneity:Tau²= 0.001;Chi²= 1.00,df= 16(P=0.001); I²= 58%
Test for overall effect: Z=-0.68;(P=0.496)

Study	Eyhance Mean	Eyhance SD	Eyhance Total	Monofocals Mean	Monofocals SD	Monofocals Total	Weight	Mean Difference IV, Random, 95% CI	A	B	C	D	E	F	G	O
**Defocus: -0.50 D**																
Mencucci 2023a	0.11	0.10	12	0.07	0.10	12	4.12	0.04 [-0.04, 0.12]	x	+	+	+	+	+	+	x
Mencucci 2023b	0.11	0.10	12	0.06	0.10	12	4.12	0.05 [-0.03, 0.13]	x	+	+	+	+	+	+	x
Mencucci 2020	0.05	0.10	20	0.10	0.10	20	5.31	-0.05 [-0.11, 0.01]	+	+	+	+	+	+	+	+
Steinmüller 2022	-0.11	0.07	15	-0.07	0.07	15	6.25	-0.04 [-0.09, 0.01]	+	+	+	+	+	+	+	+
Corbelli 2023	0.10	0.10	25	0.10	0.10	25	5.81	0.00 [-0.06, 0.06]	-	-	+	+	+	+	x	x
Choi 2023	0.03	0.10	25	0.06	0.10	25	5.81	-0.03 [-0.09, 0.03]	-	-	+	-	+	NA	NA	-
Micheletti 2023	0.07	0.07	15	0.07	0.07	15	6.25	0.00 [-0.05, 0.05]	x	x	x	+	-	x	x	x
Lopes 2021	0.11	0.10	30	0.16	0.10	30	6.20	-0.05 [-0.10, 0.00]	-	-	+	+	+	-	-	-
Corbelli 2022	0.05	0.06	25	0.08	0.06	25	7.68	-0.03 [-0.06, 0.00]	-	-	+	+	+	+	+	-
Huh 2021	-0.01	0.10	15	-0.01	0.10	15	4.64	0.00 [-0.07, 0.07]	x	-	+	+	+	-	-	x
Unsal 2021	0.06	0.10	16	0.11	0.10	16	4.79	-0.05 [-0.12, 0.02]	x	-	+	+	+	x	-	x
Eguileor 2020	-0.05	0.10	30	-0.05	0.10	30	6.20	0.00 [-0.05, 0.05]	-	-	+	-	+	NA	NA	-
Giglio 2024a	0.00	0.12	30	0.00	0.12	30	5.40	0.00 [-0.06, 0.06]	-	-	+	-	+	NA	NA	-
Giglio 2024b	0.00	0.12	30	0.03	0.12	30	5.40	-0.03 [-0.09, 0.03]	-	-	+	-	+	NA	NA	-
Donoso 2023	0.04	0.05	29	0.05	0.07	31	7.87	-0.01 [-0.04, 0.02]	+	+	+	+	+	NA	NA	+
Nanavaty 2022	-0.04	0.09	25	0.01	0.08	25	6.49	-0.05 [-0.10, -0.00]	-	x	+	-	x	NA	NA	x
Auffarth 2021	0.01	0.10	67	0.02	0.10	72	7.68	-0.01 [-0.04, 0.02]	+	+	+	+	+	NA	NA	+
**Subtotal (95% CI)**			421			428		-0.02 [-0.04, 0.00]								
**Prediction Interval**								-0.02 [-0.09, 0.06]								

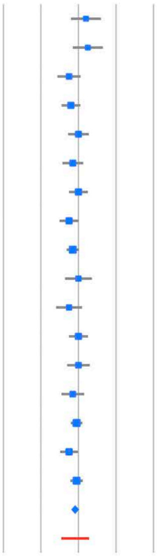

Heterogeneity:Tau²= 0.001;Chi²= 0.40,df= 16(P=0.603); I²= 0%
Test for overall effect: Z=-1.57;(P=0.115)

Study	Eyhance Mean	Eyhance SD	Eyhance Total	Monofocals Mean	Monofocals SD	Monofocals Total	Weight	Mean Difference IV, Random, 95% CI	A	B	C	D	E	F	G	O
**Defocus: -1.00 D**																
Mencucci 2023a	0.13	0.10	12	0.13	0.10	12	4.31	0.00 [-0.08, 0.08]	x	+	+	+	+	+	+	x
Mencucci 2023b	0.13	0.10	12	0.12	0.10	12	4.31	0.01 [-0.07, 0.09]	x	+	+	+	+	+	+	x
Mencucci 2020	0.09	0.10	20	0.20	0.10	20	5.55	-0.11 [-0.17, -0.05]	+	+	+	+	+	+	+	+
Steinmüller 2022	0.00	0.10	15	0.13	0.12	15	4.36	-0.13 [-0.21, -0.05]	+	+	+	+	+	+	+	+
Corbelli 2023	0.13	0.10	25	0.13	0.10	25	6.08	0.00 [-0.06, 0.06]	-	-	+	+	+	+	x	x
Choi 2023	0.07	0.10	25	0.11	0.10	25	6.08	-0.04 [-0.10, 0.02]	-	-	+	-	+	NA	NA	-
Micheletti 2023	0.16	0.11	15	0.19	0.11	15	4.38	-0.03 [-0.11, 0.05]	x	x	x	+	-	x	x	x
Lopes 2021	0.21	0.10	30	0.27	0.10	30	6.49	-0.06 [-0.11, -0.01]	-	-	+	+	+	-	-	-
Corbelli 2022	0.09	0.09	25	0.18	0.08	25	6.79	-0.09 [-0.14, -0.04]	-	-	+	+	+	+	+	-
Huh 2021	-0.01	0.10	15	0.06	0.10	15	4.85	-0.07 [-0.14, 0.00]	x	-	+	+	+	-	-	x
Unsal 2021	0.10	0.10	16	0.21	0.10	16	5.01	-0.11 [-0.18, -0.04]	x	-	+	+	+	x	-	x
Eguileor 2020	0.00	0.10	30	0.07	0.10	30	6.49	-0.07 [-0.12, -0.02]	-	-	+	-	+	NA	NA	-
Giglio 2024a	0.11	0.10	30	0.14	0.10	30	6.49	-0.03 [-0.08, 0.02]	-	-	+	-	+	NA	NA	-
Giglio 2024b	0.11	0.10	30	0.17	0.10	30	6.49	-0.06 [-0.11, -0.01]	-	-	+	-	+	NA	NA	-
Donoso 2023	0.11	0.14	29	0.18	0.17	31	4.36	-0.07 [-0.15, 0.01]	+	+	+	+	+	NA	NA	+
Nanavaty 2022	0.02	0.11	25	0.13	0.09	25	6.06	-0.11 [-0.17, -0.05]	-	x	+	-	x	NA	NA	x
Auffarth 2021	0.10	0.10	67	0.15	0.10	72	8.03	-0.05 [-0.08, -0.02]	+	+	+	+	+	NA	NA	+
Elbakry 2023	0.22	0.10	10	0.21	0.10	10	3.87	0.01 [-0.08, 0.10]	x	-	+	+	+	x	x	x
**Subtotal (95% CI)**			431			438		-0.06 [-0.08, -0.04]								
**Prediction Interval**								-0.06 [-0.13, 0.02]								

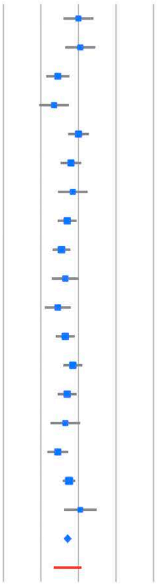

Heterogeneity:Tau²= 0.001;Chi²= 0.94,df= 17(P=0.055); I²= 37%
Test for overall effect: Z=-5.11;(P=<0.0001)

Defocus: -1.50 D

Mencucci 2023a	0.24	0.10	12	0.21	0.10	12	4.38	0.03 [-0.05, 0.11]	x	+	+	+	+	+	+	x
Mencucci 2023b	0.24	0.10	12	0.19	0.10	12	4.38	0.05 [-0.03, 0.13]	x	+	+	+	+	+	+	x
Mencucci 2020	0.20	0.10	20	0.32	0.10	20	5.65	-0.12 [-0.18, -0.06]	+	+	+	+	+	+	+	+
Steinmüller 2022	0.12	0.10	15	0.26	0.14	15	3.97	-0.14 [-0.23, -0.05]	+	+	+	+	+	+	+	+
Corbelli 2023	0.24	0.10	25	0.24	0.10	25	6.19	0.00 [-0.06, 0.06]	-	-	+	+	+	+	x	x
Choi 2023	0.10	0.10	25	0.18	0.10	25	6.19	-0.08 [-0.14, -0.02]	-	-	+	-	+	NA	NA	-
Micheletti 2023	0.28	0.12	15	0.31	0.13	15	3.84	-0.03 [-0.12, 0.06]	x	x	x	+	-	x	x	x
Lopes 2021	0.32	0.10	30	0.42	0.10	30	6.61	-0.10 [-0.15, -0.05]	-	-	+	+	+	-	-	-
Corbelli 2022	0.19	0.10	25	0.29	0.10	25	6.19	-0.10 [-0.16, -0.04]	-	-	+	+	+	+	+	-
Huh 2021	0.03	0.10	15	0.19	0.10	15	4.94	-0.16 [-0.23, -0.09]	x	-	+	+	+	-	-	x
Unsal 2021	0.25	0.10	16	0.30	0.10	16	5.10	-0.05 [-0.12, 0.02]	x	-	+	+	+	x	-	x
Eguileor 2020	0.04	0.10	30	0.21	0.10	30	6.61	-0.17 [-0.22, -0.12]	-	-	+	-	+	NA	NA	-
Giglio 2024a	0.23	0.10	30	0.29	0.10	30	6.61	-0.06 [-0.11, -0.01]	-	-	+	-	+	NA	NA	-
Giglio 2024b	0.23	0.10	30	0.30	0.10	30	6.61	-0.07 [-0.12, -0.02]	-	-	+	-	+	NA	NA	-
Donoso 2023	0.26	0.15	29	0.48	0.03	31	6.32	-0.22 [-0.27, -0.17]	+	+	+	+	+	NA	NA	+
Nanavaty 2022	0.13	0.16	25	0.28	0.13	25	4.33	-0.15 [-0.23, -0.07]	-	x	+	-	x	NA	NA	x
Auffarth 2021	0.22	0.10	67	0.30	0.10	72	8.18	-0.08 [-0.11, -0.05]	+	+	+	+	+	NA	NA	+
Elbakry 2023	0.33	0.10	10	0.33	0.10	10	3.94	0.00 [-0.09, 0.09]	x	-	+	+	+	x	x	x
**Subtotal (95% CI)**			431			438		-0.08 [-0.11, -0.06]								
**Prediction Interval**								-0.08 [-0.16, -0.01]								

Heterogeneity:Tau^2= 0.001;Chi^2= 1.00,df= 17(P=<0.0001); I^2= 79%
Test for overall effect: Z=-7.44;(P=<0.0001)

Defocus: -2.00 D

Mencucci 2023a	0.34	0.10	12	0.30	0.10	12	4.15	0.04 [-0.04, 0.12]	x	+	+	+	+	+	+	x
Mencucci 2023b	0.34	0.10	12	0.28	0.10	12	4.15	0.06 [-0.02, 0.14]	x	+	+	+	+	+	+	x
Mencucci 2020	0.29	0.10	20	0.40	0.10	20	5.35	-0.11 [-0.17, -0.05]	+	+	+	+	+	+	+	+
Steinmüller 2022	0.29	0.12	15	0.43	0.16	15	3.10	-0.14 [-0.24, -0.04]	+	+	+	+	+	+	+	+
Corbelli 2023	0.47	0.10	25	0.47	0.10	25	5.86	0.00 [-0.06, 0.06]	-	-	+	+	+	+	x	x
Choi 2023	0.16	0.10	25	0.26	0.10	25	5.86	-0.10 [-0.16, -0.04]	-	-	+	-	+	NA	NA	-
Micheletti 2023	0.44	0.16	15	0.46	0.16	15	2.61	-0.02 [-0.13, 0.09]	x	x	x	+	-	x	x	x
Lopes 2021	0.52	0.10	30	0.62	0.10	30	6.26	-0.10 [-0.15, -0.05]	-	-	+	+	+	-	-	-
Corbelli 2022	0.31	0.06	25	0.37	0.06	25	7.74	-0.06 [-0.09, -0.03]	-	-	+	+	+	+	+	-
Huh 2021	0.09	0.10	15	0.29	0.10	15	4.67	-0.20 [-0.27, -0.13]	x	-	+	+	+	-	-	x
Unsal 2021	0.32	0.10	16	0.41	0.10	16	4.83	-0.09 [-0.16, -0.02]	x	-	+	+	+	x	-	x
Eguileor 2020	0.16	0.10	30	0.36	0.10	30	6.26	-0.20 [-0.25, -0.15]	-	-	+	-	+	NA	NA	-
Giglio 2024a	0.37	0.07	30	0.44	0.07	30	7.56	-0.07 [-0.11, -0.03]	-	-	+	-	+	NA	NA	-
Giglio 2024b	0.37	0.07	30	0.46	0.07	30	7.56	-0.09 [-0.13, -0.05]	-	-	+	-	+	NA	NA	-
Donoso 2023	0.47	0.06	29	0.49	0.02	31	8.60	-0.02 [-0.04, 0.00]	+	+	+	+	+	NA	NA	+
Nanavaty 2022	0.25	0.16	25	0.40	0.14	25	3.96	-0.15 [-0.23, -0.07]	-	x	+	-	x	NA	NA	x
Auffarth 2021	0.33	0.10	67	0.41	0.10	72	7.74	-0.08 [-0.11, -0.05]	+	+	+	+	+	NA	NA	+
Elbakry 2023	0.48	0.10	10	0.46	0.10	10	3.73	0.02 [-0.07, 0.11]	x	-	+	+	+	x	x	x
**Subtotal (95% CI)**			431			438		-0.07 [-0.10, -0.05]								
**Prediction Interval**								-0.07 [-0.15, -0.00]								

Heterogeneity:Tau^2= 0.001;Chi^2= 1.00,df= 17(P=<0.0001); I^2= 82%
Test for overall effect: Z=-6.74;(P=<0.0001)

**Total (95% CI)**	2.135	2.170		-0.05 [-0.06, -0.04]
**Prediction Interval**				-0.05 [-0.14, 0.04]

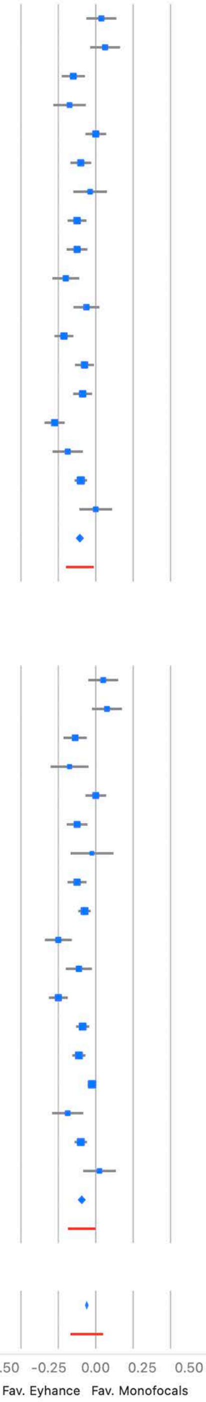

Heterogeneity:Tau^2= 0.002;Chi^2= 1.00,df= 87(P=<0.0001)
Test for overall effect: Z=-9.58;(P=<0.0001)
