# Supplementary material for: Visual and patient-reported outcomes of an enhanced versus monofocal intraocular lenses in cataract surgery: a systematic review and meta-analysis
Source: Eye (Lond). 2025 Feb 1;39(5):883–98. doi: 10.1038/s41433-025-03625-4 (PMC11933469; doi:10.1038/s41433-025-03625-4)
Supplement: Supplementary file 8 — Supplementary File A: Search strategy [file 41433_2025_3625_MOESM8_ESM.pdf]

Supplemental Material A I: Search strategy

Table A.1. MEDLINE (Pubmed)
Date searched: June 24, 2024

Search	Query	Entries
#1 Population	(cataract*[tiab] OR cataract[mh] OR implantation[tiab] OR "Lens Implantation, Intraocular"[mh] NOT (Infant[mh] OR "Young Adult"[mh] OR adolescent[mh] OR child[mh] OR "combined implantation"[ti] OR Mix-and-Match[ti] OR Mix-Match[ti] OR sulcus[ti] OR blended[ti])) AND (humans[mh] OR eyes[tiab] NOT (phakic intraocular lens*[tiab] OR "phakic intraocular lenses"[mh] OR phakic lens*[ti] OR icl[ti] OR AddOn[tiab]) OR subject*[tiab] OR patient*[tiab] NOT ("In Vitro Techniques"[mh] OR "Models, Theoretical"[mh] OR "Models, Economic"[mh] OR Economics[sh]))	172,217
#2 Intervention	(eyhance[tiab] OR enhanced[tiab] OR mono-EDOF[tiab] OR icb00[tiab])	1,056,146
#3 Comparator	(PCB00[tiab] OR monofocal[tiab])	1,472
#4 Outcome	("contrast sensitivity"[mh:NoExp] OR "visual acuity"[mh:NoExp] OR "contrast sensitivity"[tiab] OR visual acuit*[tiab] OR "patient satisfaction"[mh] OR "Patient Reported Outcome Measures"[mh:NoExp] OR satisfaction[tiab] OR "spectacle dependence"[tiab] OR "spectacle independence"[tiab] OR defocus curve*[tiab] OR Glare[tiab] OR Halo[tiab] OR Starbust[tiab] OR dysphotopsia[tiab] NOT (simulator[ti] OR prediction[ti] OR predicted[ti] OR formula[ti] OR Kappa[ti] OR Alpha[ti] OR Biometric[ti] OR "IOL exchange"[ti] OR explantation[ti] OR Preoperative[ti] OR Centration[ti] OR autorefraction[ti] OR Tilt[ti] OR Decentration[ti] OR astigmatism[ti]))	405,891
#5	2019:2024[pdat] AND #1 AND #2 AND #3 AND #4 NOT (Comment[pt] OR Review[pt])	72

Table A.2. EMBASE (Ovid)
Date searched: June 24, 2024

Search	Query	Entries
#1 Population	('cataract*':ti,ab,kw OR 'cataract'/exp OR 'implantation':ti,ab,kw OR 'lens implantation'/exp NOT ('infant'/exp OR 'young adult'/exp OR 'adolescent'/exp OR 'child'/exp OR 'combined implantation':ti OR 'mix-and-match':ti OR 'mix-match':ti OR 'sulcus':ti OR 'blended':ti)) AND ('human'/exp OR 'eyes':ti,ab,kw NOT ('phakic intraocular lens*':ti,ab,kw OR 'phakic intraocular lens'/exp OR 'phakic lens*':ti OR 'icl':ti OR 'addon':ti,ab,kw) OR 'subject*':ti,ab,kw OR 'patient*':ti,ab,kw NOT ('in vitro study'/exp OR 'theoretical model'/exp OR 'economic model'/exp))	254,938
#2 Intervention	('eyhance':ti,ab,kw OR 'enhanced':ti,ab,kw OR 'mono-edof':ti,ab,kw OR 'icb00':ti,ab,kw)	1,241,842
#3 Comparator	('pcb00':ti,ab,kw OR 'monofocal':ti,ab,kw)	2,078
#4 Outcome	('contrast sensitivity'/de OR 'visual acuity'/de OR 'contrast sensitivity':ti,ab,kw OR 'visual acuit*':ti,ab,kw OR 'patient satisfaction'/exp OR 'patient-reported outcome'/de OR 'satisfaction':ti,ab,kw OR 'spectacle dependence':ti,ab,kw OR 'spectacle independence':ti,ab,kw OR 'defocus curve*':ti,ab,kw OR 'glare':ti,ab,kw OR 'halo':ti,ab,kw OR 'starbust':ti,ab,kw OR 'dysphotopsia':ti,ab,kw NOT ('simulator':ti OR 'prediction':ti OR 'predicted':ti OR 'formula':ti OR 'kappa':ti OR 'alpha':ti OR 'biometric':ti	590,206

	OR 'iol exchange':ti OR 'explantation':ti OR 'preoperative':ti OR 'centration':ti OR 'autorefraction':ti OR 'tilt':ti OR 'decentration':ti OR 'astigmatism':ti))	
#5	[2019-2024]/py AND #1 AND #2 AND #3 AND #4 NOT (comment.pt. OR review.pt.)	71
