# Supplementary material for: Visual and patient-reported outcomes of an enhanced versus monofocal intraocular lenses in cataract surgery: a systematic review and meta-analysis
Source: Eye (Lond). 2025 Feb 1;39(5):883–98. doi: 10.1038/s41433-025-03625-4 (PMC11933469; doi:10.1038/s41433-025-03625-4)
Supplement: Supplementary file 9 — Supplementary File B: Risk of Bias Assessment with Robins-I or Rob2 [file 41433_2025_3625_MOESM9_ESM.pdf]

Risk of Bias Assessment with Robins-I or Rob2

Study: Mencucci R 2024 (Tecnis ZCB00) 2
Study: Salgado-Borges J 2024 (IsoPure) 4
Study: Giansanti F 2023 (Tecnis ZCB00) 6
Study: Mencucci R 2023a (Vivinex Impress), 2023b (IsoPure) 8
Study: Mencucci R 2020 (Tecnis ZCB00) 10
Study: Hwang, 2024 (Tecnis ZCB00) 12
Study: Singh, 2024 (Tecnis ZCB00) 14
Study: Dell 2024 (Tecnis ZCB00) 16
Study: Kozhaya 2024 (SofPort or enVista or Toric) 18
Study: Steinmüller R 2022 (Tecnis ZCB00) 20
Study: Nam 2024 (Tecnis ZCB00) 22
Study: Corbelli 2023 (Zoe Primus-HD) 24
Study: Gigon 2023 (Tecnis PCB00) 26
Study: Beltraminelli, 2023 (AcrySof SN60WF or Tecnis ZCB00) 28
Study: Micheletti, 2023 (Clareon CCA0T0 or CNA0T0) 30
Study: Lopes, 2021 (Tecnis PCB00) 32
Study: Corbelli 2022 (Tecnis ZCB00) 34
Study: Ucar 2021 (Tecnis ZCB00) 36
Study: Huh 2021 (Tecnis ZCB00) 38
Study: Kang 2021 (Tecnis ZCB00) 40
Study: Cinar 2021 (AcrySof SN60WF) 42
Study: Unsal 2021 (Tecnis ZCB00) 44
Study: Elbakry 2023 (Tecnis ZCB00) 46
Study: Eguileor 2020 (Tecnis ZCB00) 48
Study: Giglio, 2024a (Tecnis PCB00) 2024b (Clareon CNA0T0) 50
Study: Goslings 2023 (Vivinex iSert) 53
Study: Donoso 2024 (Tecnis ZCB00) 55
Study: Garzón 2022 (Tecnis ZCB00) 57
Study: Nanavaty 2022 (Tecnis ZCB00) 58
Study: Auffarth 2021 (Tecnis ZCB00) 60
Study: Choi 2023 (Tecnis ZCB00) 61

Study: Mencucci R 2024 (Tecnis ZCB00)

Comparator: Tecnis ZCB00

Risk of Bias Tool: ROBINS-I

Correspondence: Low (L) Moderate (M) Serious (S) Critical (C) Not Informed (N)

Grading by End-Point:

**Mono CDVA:**	**A:** C **B:** M **C:** L **D:** L **E:** L **F:** L **G:** L **O:** C
**Mono DCIVA:**	**A:** C **B:** M **C:** L **D:** L **E:** L **F:** L **G:** L **O:** C
**Mono DCNVA:**	**A:** N **B:** N **C:** N **D:** N **E:** N **F:** N **G:** N **O:** N
**Mono DC:**	**A:** C **B:** M **C:** L **D:** L **E:** L **F:** L **G:** L **O:** C
**Mono CSF:**	**A:** C **B:** M **C:** L **D:** L **E:** L **F:** L **G:** S **O:** C
**Bino UDVA:**	**A:** N **B:** N **C:** N **D:** N **E:** N **F:** N **G:** N **O:** N
**Bino UIVA:**	**A:** N **B:** N **C:** N **D:** N **E:** N **F:** N **G:** N **O:** N
**Bino UNVA:**	**A:** N **B:** N **C:** N **D:** N **E:** N **F:** N **G:** N **O:** N
**PROs:**	**A:** C **B:** M **C:** L **D:** L **E:** L **F:** L **G:** L **O:** C
**Bino DC:**	**A:** N **B:** N **C:** N **D:** N **E:** N **F:** N **G:** N **O:** N
**Bino CSF:**	**A:** N **B:** N **C:** N **D:** N **E:** N **F:** N **G:** N **O:** N

Comments for Decision by Domain:

A. Confounding:

- Methods for assigning interventions to participants not described.
- Potential confounding due to selecting IOLs from perspectives of DMEK surgery.
- Non-significant differences between confounders of (i.e. HOAs) shown in baseline characteristics.
- Lack of postoperative residual data could lead to confounding on binocular uncorrected measurement visual performance and PROs.

B. Selection Bias:

- Recruitment method not described (consecutive or not).
- The inclusion criteria for DMEK patients based on FECD grade and cataract severity was clear.
- Exclusion of patients with postoperative residual can result in bias for variables measured without distance correction.
- Balanced groups, but possible forced balance by patient exclusion.

C. Classification:

- IOLs and interventions clearly defined.

- Same surgeon and uniform procedures for all patients.

D. Deviations:

- No deviations reported; all patients underwent implantation without secondary interventions.

E. Missing Data:

- No missing data for any end-points; all patients included in the analysis.

F. Measuring Outcomes:

- VA: Standard tests used.
- DC: Defocus curves likely measured with distance correction, assuming low bias. Although not clearly specified in this study. Studies from the same author reported this end-point with best distance correction.
- CSF: Measured with Optec 6500 under standard conditions but refractive correction not explicitly stated.

G. Reporting Outcomes:

- CSF: Outcomes described in methods not fully reported in results, introducing bias for CSF measurement.

Study: Salgado-Borges J 2024 (IsoPure)

Comparator: IsoPure

Risk of Bias Tool: ROBINS-I

Correspondence: Low (L) Moderate (M) Serious (S) Critical (C) Not Informed (N)

Grading by End-Point:

**Mono CDVA:**	**A:** N **B:** N **C:** N **D:** N **E:** N **F:** N **G:** N **O:** N
**Mono DCIVA:**	**A:** N **B:** N **C:** N **D:** N **E:** N **F:** N **G:** N **O:** N
**Mono DCNVA:**	**A:** N **B:** N **C:** N **D:** N **E:** N **F:** N **G:** N **O:** N
**Mono DC:**	**A: S B: C C: L D: L E: L F: L G: L O: C**
**Mono CSF:**	**A:** N **B:** N **C:** N **D:** N **E:** N **F:** N **G:** N **O:** N
**Bino UDVA:**	**A:** N **B:** N **C:** N **D:** N **E:** N **F:** N **G:** N **O:** N
**Bino UIVA:**	**A:** N **B:** N **C:** N **D:** N **E:** N **F:** N **G:** N **O:** N
**Bino UNVA:**	**A:** N **B:** N **C:** N **D:** N **E:** N **F:** N **G:** N **O:** N
**PROs:**	**A:** N **B:** N **C:** N **D:** N **E:** N **F:** N **G:** N **O:** N
**Bino DC:**	**A:** N **B:** N **C:** N **D:** N **E:** N **F:** N **G:** N **O:** N
**Bino CSF:**	**A:** N **B:** N **C:** N **D:** N **E:** N **F:** N **G:** N **O:** N

Comments for Decision by Domain:

A. Confounding:

- Methods for assigning interventions to participants not described.
- No demographic comparison table between groups, increasing the potential for confounding variables.
- Potential confounding due to variables related to the outcome that were not controlled.

B. Selection Bias:

- Inclusion criteria of CDVA better than 0.1 logMAR may introduce bias by favoring patients with already good visual acuity.
- If CDVA is affected by the intervention, this bias would favor the intervention.

C. Classification:

- IOLs of intervention and comparator clearly defined. •
- Details on how surgeries were conducted were not explained, increasing the classification risk.

D. Deviations:

- No deviations reported; all patients underwent implantation.
- Moderate risk due to missing details about surgery application and procedural consistency.

E. Missing Data:

- No missing data for the reported end-point; all patients were included in the analysis.

F. Measuring Outcomes:

- DC: Measurement of defocus curves not clearly reported (with or without distance correction), increasing risk of confounding bias due to postoperative residual refraction.
- Low risk of bias overall, as the study design focused on optical bench comparison, assuming use of distance correction.

G. Reporting Outcomes:

- Authors chose the outcome to correlate with optical bench data, consistent with the purpose of the study.

Study: Giansanti F 2023 (Tecnis ZCB00)

Comparator: Tecnis ZCB00

Risk of Bias Tool: ROBINS-I

Correspondence: Low (L) Moderate (M) Serious (S) Critical (C) Not Informed (N)

Grading by End-Point:

**Mono CDVA:**	**A:** C **B:** L **C:** L **D:** L **E:** M **F:** L **G:** L **O:** C
**Mono DCIVA:**	**A:** N **B:** N **C:** N **D:** N **E:** N **F:** N **G:** N **O:** N
**Mono DCNVA:**	**A:** N **B:** N **C:** N **D:** N **E:** N **F:** N **G:** N **O:** N
**Mono DC:**	**A:** C **B:** L **C:** L **D:** L **E:** M **F:** L **G:** L **O:** C
**Mono CSF:**	**A:** N **B:** N **C:** N **D:** N **E:** N **F:** N **G:** N **O:** N
**Bino UDVA:**	**A:** N **B:** N **C:** N **D:** N **E:** N **F:** N **G:** N **O:** N
**Bino UIVA:**	**A:** C **B:** L **C:** L **D:** L **E:** M **F:** L **G:** L **O:** C
**Bino UNVA:**	**A:** N **B:** N **C:** N **D:** N **E:** N **F:** N **G:** N **O:** N
**PROs:**	**A:** N **B:** N **C:** N **D:** N **E:** N **F:** N **G:** N **O:** N
**Bino DC:**	**A:** N **B:** N **C:** N **D:** N **E:** N **F:** N **G:** N **O:** N
**Bino CSF:**	**A:** N **B:** N **C:** N **D:** N **E:** N **F:** N **G:** N **O:** N

Comments for Decision by Domain:

A. Confounding:

- No comorbidities described beyond Epiretinal Membrane.
- Comparator group included patients rejecting intervention, with poorer preoperative DCVA suggesting more severe cataract or disease progression, leading to critical confounding.

B. Selection Bias:

- Patients who rejected participation were excluded from both intervention and comparator groups, which could introduce selection bias.

C. Classification:

- IOLs for both intervention and comparator clearly defined at the intervention stage.

D. Deviations:

- No deviations discussed.
- Patients with postoperative complications requiring reintervention were excluded, minimizing bias.

E. Missing Data:

- One patient excluded due to postoperative complications, unlikely to significantly impact overall outcomes.

F. Measuring Outcomes:

- VA: Standard tests used in both groups.
- DC: Defocus curves measured with distance correction.

G. Reporting Outcomes:

- Main end-points of interest were reported.

Study: Mencucci R 2023a (Vivinex Impress), 2023b (IsoPure)

Comparator: Vivinex Impress (a) y IsoPure (b)

Risk of Bias Tool: ROBINS-I

Correspondence: Low (L) Moderate (M) Serious (S) Critical (C) Not Informed (N)

Grading by End-Point:

**Mono CDVA:**	**A:** C **B:** L **C:** L **D:** L **E:** L **F:** L **G:** L **O:** C
**Mono DCIVA:**	**A:** C **B:** L **C:** L **D:** L **E:** L **F:** L **G:** L **O:** C
**Mono DCNVA:**	**A:** C **B:** L **C:** L **D:** L **E:** L **F:** L **G:** L **O:** C
**Mono DC:**	**A:** N **B:** N **C:** N **D:** N **E:** N **F:** N **G:** N **O:** N
**Mono CSF:**	**A:** N **B:** N **C:** N **D:** N **E:** N **F:** N **G:** N **O:** N
**Bino UDVA:**	**A:** C **B:** L **C:** L **D:** L **E:** L **F:** L **G:** L **O:** C
**Bino UIVA:**	**A:** C **B:** L **C:** L **D:** L **E:** L **F:** L **G:** L **O:** C
**Bino UNVA:**	**A:** C **B:** L **C:** L **D:** L **E:** L **F:** L **G:** L **O:** C
**PROs:**	**A:** C **B:** L **C:** L **D:** L **E:** L **F:** L **G:** L **O:** C
**Bino DC:**	**A:** C **B:** L **C:** L **D:** L **E:** L **F:** L **G:** L **O:** C
**Bino CSF:**	**A:** N **B:** N **C:** N **D:** N **E:** N **F:** N **G:** N **O:** N

Comments for Decision by Domain:

A. Confounding:

- Methods for assigning interventions not described.
- Confounding factors seem controlled, but subjective refraction was not reported, potentially leading to bias.
- Possible bias from objective refraction used to obtain best-corrected visual acuity (hyperopic shift suggested in Eyhance defocus curve).

B. Selection Bias:

- Inclusion and exclusion criteria do not suggest any selection bias.

C. Classification:

- IOLs for both intervention and comparator clearly defined at the intervention stage.

D. Deviations:

- No deviations reported; all patients included in the analysis.

E. Missing Data:

- No missing data for the reported end-point; all patients included in the analysis.

F. Measuring Outcomes:

- VA: Standard tests used in both groups.
- DC and CSF measured binocularly as secondary outcomes.

G. Reporting Outcomes:

- Binocular outcomes reported for DC & CSF.
- Primary visual acuity outcomes reported with and without correction, meeting all required metrics.

Study: Mencucci R 2020 (Tecnis ZCB00)

Comparator: Tecnis ZCB00

Risk of Bias Tool: ROBINS-I

Correspondence: Low (L) Moderate (M) Serious (S) Critical (C) Not Informed (N)

Grading by End-Point:

**Mono CDVA:**	**A:** L **B:** L **C:** L **D:** L **E:** L **F:** L **G:** L **O:** L
**Mono DCIVA:**	**A:** L **B:** L **C:** L **D:** L **E:** L **F:** L **G:** L **O:** L
**Mono DCNVA:**	**A:** L **B:** L **C:** L **D:** L **E:** L **F:** L **G:** L **O:** L
**Mono DC:**	**A:** N **B:** N **C:** N **D:** N **E:** N **F:** N **G:** N **O:** N
**Mono CSF:**	**A:** N **B:** N **C:** N **D:** N **E:** N **F:** N **G:** N **O:** N
**Bino UDVA:**	**A:** L **B:** L **C:** L **D:** L **E:** L **F:** L **G:** L **O:** L
**Bino UIVA:**	**A:** L **B:** L **C:** L **D:** L **E:** L **F:** L **G:** L **O:** L
**Bino UNVA:**	**A:** L **B:** L **C:** L **D:** L **E:** L **F:** L **G:** L **O:** L
**PROs:**	**A:** N **B:** N **C:** N **D:** N **E:** N **F:** N **G:** N **O:** N
**Bino DC:**	**A:** L **B:** L **C:** L **D:** L **E:** L **F:** L **G:** L **O:** L
**Bino CSF:**	**A:** N **B:** N **C:** N **D:** N **E:** N **F:** N **G:** N **O:** N

Comments for Decision by Domain:

A. Confounding:

- Methods for assigning interventions not described, but restrictive inclusion criteria similar to a randomized clinical trial.
- Confounding factors checked, with subjective refraction reported (unlike other studies by the same author).
- Manifest differences between objective and subjective spherical equivalents highlight the potential for objective refraction as a confounding factor in other studies. However, in this study authors reported the subjective spherical equivalent and the objective was only reported to highlight differences.

B. Selection Bias:

- Inclusion and exclusion criteria do not suggest any selection bias.

C. Classification:

- IOLs for both intervention and comparator clearly defined at the intervention stage.

D. Deviations:

- No deviations reported; all patients included in the analysis.

E. Missing Data:

- No missing data; all patients completed all follow-up visits and were analyzed.

F. Measuring Outcomes:

- VA: Standard tests used in both groups.
- DC and CSF measured binocularly as secondary outcomes.

G. Reporting Outcomes:

- Binocular outcomes reported for DC & CSF.
- Primary visual acuity outcomes reported with and without correction, meeting all required metrics.

Study: Hwang, 2024 (Tecnis ZCB00)

Comparator: Tecnis ZCB00

Risk of Bias Tool: ROBINS-I

Correspondence: Low (L) Moderate (M) Serious (S) Critical (C) Not Informed (N)

Grading by End-Point:

**Mono UVA:**	**A:** S **B:** C **C:** L **D:** C **E:** M **F:** S **G:** M **O:** C
**Mono CDVA:**	**A:** N **B:** N **C:** N **D:** N **E:** N **F:** N **G:** N **O:** N
**Mono DCIVA:**	**A:** N **B:** N **C:** N **D:** N **E:** N **F:** N **G:** N **O:** N
**Mono DCNVA:**	**A:** N **B:** N **C:** N **D:** N **E:** N **F:** N **G:** N **O:** N
**Mono DC:**	**A:** N **B:** N **C:** N **D:** N **E:** N **F:** N **G:** N **O:** N
**Mono CSF:**	**A:** N **B:** N **C:** N **D:** N **E:** N **F:** N **G:** N **O:** N
**Bino UDVA:**	**A:** N **B:** N **C:** N **D:** N **E:** N **F:** N **G:** N **O:** N
**Bino UIVA:**	**A:** N **B:** N **C:** N **D:** N **E:** N **F:** N **G:** N **O:** N
**Bino UNVA:**	**A:** N **B:** N **C:** N **D:** N **E:** N **F:** N **G:** N **O:** N
**PROs:**	**A:** N **B:** N **C:** N **D:** N **E:** N **F:** N **G:** N **O:** N
**Bino DC:**	**A:** N **B:** N **C:** N **D:** N **E:** N **F:** N **G:** N **O:** N
**Bino CSF:**	**A:** N **B:** N **C:** N **D:** N **E:** N **F:** N **G:** N **O:** N

Comments for Decision by Domain:

A. Confounding:

- Methods for assigning interventions not described.
- Confounding factors, such as disease stage and ratio differences, not statistically tested, potentially affecting outcomes.
- Only uncorrected endpoints described, though postoperative residual spherical equivalent was similar between groups.

B. Selection Bias:

- No description of intervention assignment methods, introducing potential bias based on surgeon expectations related to disease stage.

C. Classification:

- IOLs for both intervention and comparator clearly defined at the intervention stage.

D. Deviations:

- Some patients received cointerventions due to their diseases, but the timing of these interventions is not described.

E. Missing Data:

- No clear statement confirming the absence of missing data, and the study's characteristics suggest this could be an issue.

F. Measuring Outcomes:

- Non-standard charts used for measuring visual acuity under favorable illumination conditions (500 lux), potentially enhancing outcomes compared to standard randomized clinical trials.
- Measurements taken after >1 month, but follow-up may not have been uniform.

G. Reporting Outcomes:

- Only uncorrected visual acuity outcomes reported, as described in the methods.
- Some outcomes, such as photic phenomena, briefly reported.

Study: Singh, 2024 (Tecnis ZCB00)

Comparator: Tecnis ZCB00

Risk of Bias Tool: ROBINS-I

Correspondence: Low (L) Moderate (M) Serious (S) Critical (C) Not Informed (N)

Grading by End-Point:

Mono CDVA: **A:** C **B:** L **C:** L **D:** L **E:** L **F:** M **G:** L **O:** C

Mono DCIVA: **A:** N **B:** N **C:** N **D:** N **E:** N **F:** N **G:** N **O:** N

Mono DCNVA: **A:** N **B:** N **C:** N **D:** N **E:** N **F:** N **G:** N **O:** N

Mono DC: **A:** C **B:** L **C:** L **D:** L **E:** L **F:** C **G:** L **O:** C

Mono CSF: **A:** N **B:** N **C:** N **D:** N **E:** N **F:** N **G:** N **O:** N

Bino UDVA: **A:** N **B:** N **C:** N **D:** N **E:** N **F:** N **G:** N **O:** N

Bino UIVA: **A:** N **B:** N **C:** N **D:** N **E:** N **F:** N **G:** N **O:** N

Bino UNVA: **A:** N **B:** N **C:** N **D:** N **E:** N **F:** N **G:** N **O:** N

PROs: **A:** N **B:** N **C:** N **D:** N **E:** N **F:** N **G:** N **O:** N

Bino DC: **A:** N **B:** N **C:** N **D:** N **E:** N **F:** N **G:** N **O:** N

Bino CSF: **A:** N **B:** N **C:** N **D:** N **E:** N **F:** N **G:** N **O:** N

Comments for Decision by Domain:

A. Confounding:

- Only age described as a confounding factor.
- Important variables like CDVA (from inclusion criteria) and other biometric variables not reported.

B. Selection Bias:

- No methods described for assigning interventions, but inclusion criteria did not appear to cause selection bias.

C. Classification:

- IOLs for both intervention and comparator clearly defined at the intervention stage.

D. Deviations:

- No deviations reported.

E. Missing Data:

- No missing data; all patients included in the analysis for the reported end-point.

F. Measuring Outcomes:

- VA: DCIVA reported as a primary end-point, but CNVA was documented instead, likely an error. The outcomes did not match expectations for DCIVA, suggesting confusion with CIVA.
- Non-standard charts used for visual acuity measurements, and the distance for measuring distance-corrected visual acuity was too close.
- DC: Unclear if defocus curves were measured with distance correction. A bias at 0 D of defocus, inconsistent with CDVA, suggests favoring the intervention.

G. Reporting Outcomes:

- Authors likely failed in correctly describing intermediate visual acuity measurements

Study: Dell 2024 (Tecnis ZCB00)

Comparator: Tecnis ZCB00

Risk of Bias Tool: ROBINS-I

Correspondence: Low (L) Moderate (M) Serious (S) Critical (C) Not Informed (N)

Grading by End-Point:

**Mono CDVA:**	**A:** M **B:** M **C:** L **D:** L **E:** L **F:** M **G:** L **O:** M
**Mono DCIVA:**	**A:** N **B:** N **C:** N **D:** N **E:** N **F:** N **G:** N **O:** N
**Mono DCNVA:**	**A:** N **B:** N **C:** N **D:** N **E:** N **F:** N **G:** N **O:** N
**Mono DC:**	**A:** N **B:** N **C:** N **D:** N **E:** N **F:** N **G:** N **O:** N
**Mono CSF:**	**A:** N **B:** N **C:** N **D:** N **E:** N **F:** N **G:** N **O:** N
**Bino UDVA:**	**A:** M **B:** M **C:** L **D:** L **E:** L **F:** C **G:** L **O:** C
**Bino UIVA:**	**A:** M **B:** M **C:** L **D:** L **E:** L **F:** C **G:** L **O:** C
**Bino UNVA:**	**A:** M **B:** M **C:** L **D:** L **E:** L **F:** C **G:** L **O:** C
**PROs:**	**A:** M **B:** M **C:** L **D:** L **E:** L **F:** C **G:** L **O:** C
**Bino DC:**	**A:** N **B:** N **C:** N **D:** N **E:** N **F:** N **G:** N **O:** N
**Bino CSF:**	**A:** N **B:** N **C:** N **D:** N **E:** N **F:** N **G:** N **O:** N

Comments for Decision by Domain:

A. Confounding:

- Patients underwent RLE or Cataract surgery, but the ratio is unspecified.
- Controller group selected to match demographic characteristics, but the mean preoperative CDVA (-0.01) suggests RLE, which is inconsistent with cataract-only implantation for the controller.

B. Selection Bias:

- Controller group consciously selected to match demographic characteristics (age, IOL power, etc.).
- Only patients attending the 1-month visit included, suggesting potential exclusion of patients, leading to possible bias.

C. Classification:

- IOLs for both intervention and comparator clearly defined at the intervention stage.

D. Deviations:

- No deviations expected; all patients underwent intraocular implantation.

E. Missing Data:

- No missing data reported for the sample.

F. Measuring Outcomes:

- VA: No distance-corrected visual acuity measurements reported beyond CDVA.
- Uncorrected visual acuity reported, but micro-monovision targeting introduces bias.
- Low bias for CDVA, but critical bias for uncorrected visual acuity and PROs.

G. Reporting Outcomes:

- Only uncorrected visual acuity outcomes reported, as described in the methods.
- Photic phenomena outcomes briefly reported.

Study: Kozhaya 2024 (SofPort or enVista or Toric)

Comparator: SofPort or enVista or Toric

Risk of Bias Tool: ROBINS-I

Correspondence: Low (L) Moderate (M) Serious (S) Critical (C) Not Informed (N)

Grading by End-Point:

Mono CDVA: **A:** C **B:** C **C:** C **D:** L **E:** L **F:** L **G:** L **O:** C

Mono DCIVA: **A:** C **B:** C **C:** C **D:** L **E:** L **F:** L **G:** L **O:** C

Mono DCNVA: **A:** C **B:** C **C:** C **D:** L **E:** L **F:** L **G:** L **O:** C

Mono DC: **A:** N **B:** N **C:** N **D:** N **E:** N **F:** N **G:** N **O:** N

Mono CSF: **A:** N **B:** N **C:** N **D:** N **E:** N **F:** N **G:** N **O:** N

Bino UDVA: **A:** N **B:** N **C:** N **D:** N **E:** N **F:** N **G:** N **O:** N

Bino UIVA: **A:** N **B:** N **C:** N **D:** N **E:** N **F:** N **G:** N **O:** N

Bino UNVA: **A:** N **B:** N **C:** N **D:** N **E:** N **F:** N **G:** N **O:** N

PROs: **A:** N **B:** N **C:** N **D:** N **E:** N **F:** N **G:** N **O:** N

Bino DC: **A:** N **B:** N **C:** N **D:** N **E:** N **F:** N **G:** N **O:** N

Bino CSF: **A:** N **B:** N **C:** N **D:** N **E:** N **F:** N **G:** N **O:** N

Comments for Decision by Domain:

A. Confounding:

- No demographic table presented to assess potential confounders.
- Including toric and spherical IOLs in different groups suggests possible confounding factors.

B. Selection Bias:

- Inclusion criteria of CDVA better than 0.1 logMAR could introduce bias by favoring patients with already good visual acuity.
- Only the eye with the best distance visual acuity included per patient, further increasing bias.

C. Classification:

- IOLs in the control group varied, with different intervention IOLs mixed.

D. Deviations:

- No deviations expected; all patients underwent intraocular implantation.

E. Missing Data:

- No missing data reported for the recruited patients.

F. Measuring Outcomes:

- Follow-up reported as more than 3 weeks, suggesting potential non-uniformity in follow-up timing.

G. Reporting Outcomes:

- Only distance-corrected visual acuity outcomes reported, as the study's purpose was to evaluate the effect of spherical aberration, not to compare intervention and control groups.

Study: Steinmüller R 2022 (Tecnis ZCB00)

Comparator: Tecnis ZCB00

Risk of Bias Tool: ROBINS-I

Correspondence: Low (L) Moderate (M) Serious (S) Critical (C) Not Informed (N)

Grading by End-Point:

**Mono CDVA:**	**A:** L **B:** L **C:** L **D:** L **E:** L **F:** L **G:** L **O:** L
**Mono DCIVA:**	**A:** L **B:** L **C:** L **D:** L **E:** L **F:** L **G:** L **O:** L
**Mono DCNVA:**	**A:** L **B:** L **C:** L **D:** L **E:** L **F:** L **G:** L **O:** L
**Mono DC:**	**A:** L **B:** L **C:** L **D:** L **E:** L **F:** L **G:** L **O:** L
**Mono CSF:**	**A:** L **B:** L **C:** L **D:** L **E:** L **F:** L **G:** L **O:** L
**Bino UDVA:**	**A:** N **B:** N **C:** N **D:** N **E:** N **F:** N **G:** N **O:** N
**Bino UIVA:**	**A:** L **B:** L **C:** L **D:** L **E:** L **F:** L **G:** L **O:** L
**Bino UNVA:**	**A:** L **B:** L **C:** L **D:** L **E:** L **F:** L **G:** L **O:** L
**PROs:**	**A:** L **B:** L **C:** L **D:** L **E:** L **F:** L **G:** L **O:** L
**Bino DC:**	**A:** L **B:** L **C:** L **D:** L **E:** L **F:** L **G:** L **O:** L
**Bino CSF:**	**A:** N **B:** N **C:** N **D:** N **E:** N **F:** N **G:** N **O:** N

Comments for Decision by Domain:

A. Confounding:

- No decision criteria specified for assigning patients to groups for IOL implantation.
- Complete demographic table provided, suggesting no significant confounding factors.

B. Selection Bias:

- Prospective non-randomized study with follow-up loss of only one patient per group, unlikely to affect outcomes.

C. Classification:

- IOLs for both intervention and comparator clearly defined at the intervention stage.

D. Deviations:

- One case of cystoid macular edema reported in each group at the one-month follow-up, treated successfully without complications or visual impairment.

E. Missing Data:

- Missing data limited to one case per group, unlikely to impact outcomes.

F. Measuring Outcomes:

- Standard tests and methods of analysis used in both groups.

G. Reporting Outcomes:

- Authors reported all standard measurements.

Study: Nam 2024 (Tecnis ZCB00)

Comparator: Tecnis ZCB00

Risk of Bias Tool: ROBINS-I

Correspondence: Low (L) Moderate (M) Serious (S) Critical (C) Not Informed (N)

Grading by End-Point:

**Mono CDVA:**	**A:** N **B:** N **C:** N **D:** N **E:** N **F:** N **G:** N **O:** N
**Mono DCIVA:**	**A:** N **B:** N **C:** N **D:** N **E:** N **F:** N **G:** N **O:** N
**Mono DCNVA:**	**A:** N **B:** N **C:** N **D:** N **E:** N **F:** N **G:** N **O:** N
**Mono DC:**	**A:** L **B:** L **C:** L **D:** L **E:** L **F:** L **G:** L **O:** L
**Mono CSF:**	**A:** L **B:** L **C:** L **D:** L **E:** L **F:** L **G:** L **O:** L
**Bino UDVA:**	**A:** N **B:** N **C:** N **D:** N **E:** N **F:** N **G:** N **O:** N
**Bino UIVA:**	**A:** N **B:** N **C:** N **D:** N **E:** N **F:** N **G:** N **O:** N
**Bino UNVA:**	**A:** N **B:** N **C:** N **D:** N **E:** N **F:** N **G:** N **O:** N
**PROs:**	**A:** L **B:** L **C:** L **D:** L **E:** L **F:** L **G:** L **O:** L
**Bino DC:**	**A:** N **B:** N **C:** N **D:** N **E:** N **F:** N **G:** N **O:** N
**Bino CSF:**	**A:** N **B:** N **C:** N **D:** N **E:** N **F:** N **G:** N **O:** N

Comments for Decision by Domain:

A. Confounding:

- A demographic table was included, and no relevant differences between groups were found for potential confounders.

B. Selection Bias:

- Patients selected IOLs based on lifestyle, personality, occupation, hobbies, and eye characteristics, which may introduce selection bias.

C. Classification:

- IOLs for both intervention and comparator clearly defined at the intervention stage.

D. Deviations:

- No complications or adverse events reported that required cointervention.

E. Missing Data:

- No missing data or follow-ups reported.

F. Measuring Outcomes:

- Standard tests and methods of analysis used in both groups.

G. Reporting Outcomes:

- Authors reported all standard measurements.

Study: Corbelli 2023 (Zoe Primus-HD)

Comparator: Zoe Primus-HD

Risk of Bias Tool: ROBINS-I

Correspondence: Low (L) Moderate (M) Serious (S) Critical (C) Not Informed (N)

Grading by End-Point:

Mono CDVA: **A: L B: M C: L D: L E: L F: L G: L O: M**

Mono DCIVA: **A: L B: M C: L D: L E: L F: L G: L O: M**

Mono DCNVA: **A: L B: M C: L D: L E: L F: L G: S O: S**

Mono DC: **A: N B: N C: N D: N E: N F: N G: N O: N**

Mono CSF: **A: N B: N C: N D: N E: N F: N G: N O: N**

Bino UDVA: **A: C B: M C: L D: L E: L F: L G: L O: C**

Bino UIVA: **A: C B: M C: L D: L E: L F: L G: L O: C**

Bino UNVA: **A: C B: M C: L D: L E: L F: L G: L O: C**

PROs: **A: C B: M C: L D: L E: L F: L G: S O: C**

Bino DC: **A: M B: M C: L D: L E: L F: L G: S O: S**

Bino CSF: **A: N B: N C: N D: N E: N F: N G: N O: N**

Comments for Decision by Domain:

A. Confounding:

- Decision criteria for assigning patients to groups not specified.
- Complete demographic table included, suggesting no important confounding factors for primary outcomes.
- Risk of confounding in secondary outcomes (without distance correction) due to lack of postoperative residual error reporting.

B. Selection Bias:

- Patients with intraoperative or postoperative complications excluded, but the number of excluded eyes per group not reported, leading to possible selection bias.

C. Classification:

- IOLs for both intervention and comparator clearly defined at the intervention stage.

D. Deviations:

- No deviations, as complications were excluded as part of selection bias at the preoperative stage.

E. Missing Data:

- All subjects completed the follow-up.

F. Measuring Outcomes:

- All measurements conducted similarly in both groups following standard procedures.

G. Reporting Outcomes:

- Lack of agreement between % of eyes achieving UNVA ≤ 0.2 logMAR (0%) and the mean UNVA, suggesting reporting bias.
- Errors in reporting the defocus curve, with the -2.5 D point shown but not the -2.0 D point, making it difficult to verify the reliability of the UNVA. Binocular defocus curves were measured with best distance correction.

Study: Gigon 2023 (Tecnis PCB00)

Comparator: Tecnis PCB00

Risk of Bias Tool: ROBINS-I

Correspondence: Low (L) Moderate (M) Serious (S) Critical (C) Not Informed (N)

Grading by End-Point:

Mono CDVA: **A:** M **B:** C **C:** L **D:** L **E:** C **F:** M **G:** C **O:** C

Mono DCIVA: **A:** M **B:** C **C:** L **D:** L **E:** C **F:** M **G:** C **O:** C

Mono DCNVA: **A:** M **B:** C **C:** L **D:** L **E:** C **F:** M **G:** C **O:** C

Mono DC: **A:** N **B:** N **C:** N **D:** N **E:** N **F:** N **G:** N **O:** N

Mono CSF: **A:** N **B:** N **C:** N **D:** N **E:** N **F:** N **G:** N **O:** N

Bino UDVA: **A:** C **B:** C **C:** L **D:** L **E:** C **F:** M **G:** C **O:** C

Bino UIVA: **A:** C **B:** C **C:** L **D:** L **E:** C **F:** M **G:** C **O:** C

Bino UNVA: **A:** C **B:** C **C:** L **D:** L **E:** C **F:** M **G:** C **O:** C

PROs: **A:** C **B:** C **C:** L **D:** L **E:** C **F:** M **G:** C **O:** C

Bino DC: **A:** N **B:** N **C:** N **D:** N **E:** N **F:** N **G:** N **O:** N

Bino CSF: **A:** N **B:** N **C:** N **D:** N **E:** N **F:** N **G:** N **O:** N

Comments for Decision by Domain:

A. Confounding:

- Methods for assigning interventions not described.
- Demographic table shows differing targets between groups, making measurements without correction unreliable due to SE as a confounding factor.
- This issue should not affect distance-corrected visual acuities.

B. Selection Bias:

- Inclusion criteria of CDVA better than 0.1 logMAR introduces potential selection bias by favoring patients with good pre-existing visual acuity, which could benefit the intervention.

C. Classification:

- IOLs for both intervention and comparator clearly defined.
- No explanation provided on how surgeries were performed.

D. Deviations:

- Complications excluded as part of preoperative selection bias, with no reported deviations.

E. Missing Data:

- A significant number of patients with missing data were excluded.

F. Measuring Outcomes:

- Non-standard methods for measuring visual acuity were used, introducing the potential for bias.

G. Reporting Outcomes:

- VA: Authors did not properly report the means and standard deviations, preventing the outcomes from being used in meta-analysis.

Study: Beltraminelli, 2023 (AcrySof SN60WF or Tecnis ZCB00)

Comparator: AcrySof SN60WF or ZCB00

Risk of Bias Tool: ROBINS-I

Correspondence: Low (L) Moderate (M) Serious (S) Critical (C) Not Informed (N)

Grading by End-Point:

**Mono CDVA:**	**A:** N **B:** N **C:** N **D:** N **E:** N **F:** N **G:** N **O:** N
**Mono DCIVA:**	**A:** N **B:** N **C:** N **D:** N **E:** N **F:** N **G:** N **O:** N
**Mono DCNVA:**	**A:** N **B:** N **C:** N **D:** N **E:** N **F:** N **G:** N **O:** N
**Mono DC:**	**A:** N **B:** N **C:** N **D:** N **E:** N **F:** N **G:** N **O:** N
**Mono CSF:**	**A:** N **B:** N **C:** N **D:** N **E:** N **F:** N **G:** N **O:** N
**Bino UDVA:**	**A:** C **B:** L **C:** C **D:** L **E:** L **F:** S **G:** L **O:** C
**Bino UIVA:**	**A:** C **B:** L **C:** C **D:** L **E:** L **F:** S **G:** L **O:** C
**Bino UNVA:**	**A:** C **B:** L **C:** C **D:** L **E:** L **F:** S **G:** L **O:** C
**PROs:**	**A:** N **B:** N **C:** N **D:** N **E:** N **F:** N **G:** N **O:** N
**Bino DC:**	**A:** N **B:** N **C:** N **D:** N **E:** N **F:** N **G:** N **O:** N
**Bino CSF:**	**A:** N **B:** N **C:** N **D:** N **E:** N **F:** N **G:** N **O:** N

Comments for Decision by Domain:

A. Confounding:

- Objective refraction used instead of subjective refraction; although no differences between groups were found, this is not considered a valid correction for confounding.

B. Selection Bias:

- Patients chose between Standard and Enhanced IOLs during the pre-operative visit, based on preferences after discussion with the surgeon. This choice is unlikely to influence the primary endpoints.

C. Classification:

- Two different monofocal IOLs were used in the control group.

D. Deviations:

- No deviations reported from the included patients.

E. Missing Data:

- All subjects completed the follow-up visit.

F. Measuring Outcomes:

- Non-standard Measurements taken after >3 months, but follow-up may not have been uniform.
- Evaluators were blinded to the implanted IOLs, reducing measurement bias.

G. Reporting Outcomes:

- Outcomes aligned with the study's purpose of evaluating the micro-monovision approach, with an emphasis on binocular efficacy.

Study: Micheletti, 2023 (Clareon CCA0T0 or CNA0T0)

Comparator: Clareon CCA0T0 or CNA0T0

Risk of Bias Tool: ROBINS-I

Correspondence: Low (L) Moderate (M) Serious (S) Critical (C) Not Informed (N)

Grading by End-Point:

Mono CDVA: **A:** N **B:** N **C:** N **D:** N **E:** N **F:** N **G:** N **O:** N

Mono DCIVA: **A:** N **B:** N **C:** N **D:** N **E:** N **F:** N **G:** N **O:** N

Mono DCNVA: **A:** N **B:** N **C:** N **D:** N **E:** N **F:** N **G:** N **O:** N

Mono DC: **A:** N **B:** N **C:** N **D:** N **E:** N **F:** N **G:** N **O:** N

Mono CSF: **A:** N **B:** N **C:** N **D:** N **E:** N **F:** N **G:** N **O:** N

Bino UDVA: **A:** N **B:** N **C:** N **D:** N **E:** N **F:** N **G:** N **O:** N

Bino UIVA: **A:** N **B:** N **C:** N **D:** N **E:** N **F:** N **G:** N **O:** N

Bino UNVA: **A:** N **B:** N **C:** N **D:** N **E:** N **F:** N **G:** N **O:** N

PROs: **A:** N **B:** N **C:** N **D:** N **E:** N **F:** N **G:** N **O:** N

Bino DC: **A:** C **B:** C **C:** S **D:** L **E:** M **F:** M **G:** M **O:** C

Bino CSF: **A:** N **B:** N **C:** N **D:** N **E:** N **F:** N **G:** N **O:** N

Comments for Decision by Domain:

A. Confounding:

- A table of demographic variables was included, but confounding factors were not tested with inferential statistics. Furthermore, although the control group required of treating corneal astigmatism, this is not explained how it was managed. However, this was reduced in the postoperative period. For instance, with patients reducing more than 1 D their astigmatism after surgery without describing the application of any correction procedure in the comparator group.

B. Selection Bias:

- Sample biased by including patients with higher astigmatism in the intervention group, which required toric IOLs, while the control group did not include toric IOLs due to unavailability even though those were also required.
- This represents a critical bias overall, especially as a less biased study could have been conducted with half of the sample including only spherical IOLs in both groups.
- Patients with intraoperative or postoperative complications were excluded, but the number of excluded eyes per group was not reported, leading to possible selection bias.

C. Classification:

- Two different monofocal IOLs used in the intervention group, but clearly defined.

D. Deviations:

- Complications were excluded as part of selection bias at the preoperative stage, so no deviations were reported.

E. Missing Data:

- Text does not specify missing data handling, but all analyses included data from all subjects without data imputation, implying complete data inclusion.

F. Measuring Outcomes:

- Measurements taken after >3 months, but follow-up may not have been uniform.
- Evaluators were blinded to the implanted IOLs, reducing measurement bias.
- Defocus curves were measured with best distance correction.

G. Reporting Outcomes:

- Only binocular visual acuities were reported, making it impossible to evaluate IOL efficacy

Study: Lopes, 2021 (Tecnis PCB00)

Comparator: Tecnis PCB00

Risk of Bias Tool: ROBINS-I

Correspondence: Low (L) Moderate (M) Serious (S) Critical (C) Not Informed (N)

Grading by End-Point:

**Mono CDVA:**	**A:** N **B:** N **C:** N **D:** N **E:** N **F:** N **G:** N **O:** N
**Mono DCIVA:**	**A:** N **B:** N **C:** N **D:** N **E:** N **F:** N **G:** N **O:** N
**Mono DCNVA:**	**A:** N **B:** N **C:** N **D:** N **E:** N **F:** N **G:** N **O:** N
**Mono DC:**	**A:** N **B:** N **C:** N **D:** N **E:** N **F:** N **G:** N **O:** N
**Mono CSF:**	**A:** N **B:** N **C:** N **D:** N **E:** N **F:** N **G:** N **O:** N
**Bino UDVA:**	**A:** M **B:** M **C:** L **D:** L **E:** L **F:** M **G:** M **O:** M
**Bino UIVA:**	**A:** M **B:** M **C:** L **D:** L **E:** L **F:** M **G:** M **O:** M
**Bino UNVA:**	**A:** N **B:** N **C:** N **D:** N **E:** N **F:** N **G:** N **O:** N
**PROs:**	**A:** M **B:** M **C:** L **D:** L **E:** L **F:** M **G:** M **O:** M
**Bino DC:**	**A:** M **B:** M **C:** L **D:** L **E:** L **F:** M **G:** M **O:** M
**Bino CSF:**	**A:** N **B:** N **C:** N **D:** N **E:** N **F:** N **G:** N **O:** N

Comments for Decision by Domain:

A. Confounding:

- A demographic table was included, and no differences were found for the selected confounders.

B. Selection Bias:

- Patients with intraoperative or postoperative complications were excluded, but the number of excluded eyes per group was not reported, leading to possible selection bias.

C. Classification:

- IOLs for both intervention and comparator groups were clearly defined at the intervention stage.

D. Deviations:

- Complications excluded as part of selection bias at the preoperative stage, so no deviations were reported.

E. Missing Data:

- The text does not specify whether patients with missing data were recruited or excluded.

F. Measuring Outcomes:

- All measurements were conducted similarly in both groups, but it is not specified if standard procedures were followed.

G. Reporting Outcomes:

- Only binocular visual acuities were reported, making not possible to assess IOL efficacy.

Study: Corbelli 2022 (Tecnis ZCB00)

Comparator: Tecnis ZCB00

Risk of Bias Tool: ROBINS-I

Correspondence: Low (L) Moderate (M) Serious (S) Critical (C) Not Informed (N)

Grading by End-Point:

**Mono CDVA:**	**A:** M **B:** M **C:** L **D:** L **E:** L **F:** L **G:** L **O:** M
**Mono DCIVA:**	**A:** M **B:** M **C:** L **D:** L **E:** L **F:** L **G:** L **O:** M
**Mono DCNVA:**	**A:** M **B:** M **C:** L **D:** L **E:** L **F:** L **G:** L **O:** M
**Mono DC:**	**A:** N **B:** N **C:** N **D:** N **E:** N **F:** N **G:** N **O:** N
**Mono CSF:**	**A:** N **B:** N **C:** N **D:** N **E:** N **F:** N **G:** N **O:** N
**Bino UDVA:**	**A:** M **B:** M **C:** L **D:** L **E:** L **F:** L **G:** L **O:** M
**Bino UIVA:**	**A:** M **B:** M **C:** L **D:** L **E:** L **F:** L **G:** L **O:** M
**Bino UNVA:**	**A:** M **B:** M **C:** L **D:** L **E:** L **F:** L **G:** L **O:** M
**PROs:**	**A:** N **B:** N **C:** N **D:** N **E:** N **F:** N **G:** N **O:** N
**Bino DC:**	**A:** M **B:** M **C:** L **D:** L **E:** L **F:** L **G:** L **O:** M
**Bino CSF:**	**A:** M **B:** M **C:** L **D:** L **E:** L **F:** L **G:** L **O:** M

Comments for Decision by Domain:

A. Confounding:

- Lens choice based on patient requests and clinician suggestions.
- Demographic table included, suggesting no significant confounding factors

B. Selection Bias:

- Patients with intraoperative or postoperative complications excluded, but the number of excluded eyes per group not reported, leading to possible selection bias.

C. Classification:

- IOLs for both intervention and comparator clearly defined at the intervention stage.

D. Deviations:

- Complications excluded as part of selection bias at the preoperative stage, so no deviations were reported.

E. Missing Data:

- All subjects completed the follow-up.

F. Measuring Outcomes:

- All measurements conducted similarly in both groups following standard procedures.

G. Reporting Outcomes:

- Authors reported only secondary outcomes for defocus curves and contrast sensitivity (binocular).

Study: Ucar 2021 (Tecnis ZCB00)

Comparator: Tecnis ZCB00

Risk of Bias Tool: ROBINS-I

Correspondence: Low (L) Moderate (M) Serious (S) Critical (C) Not Informed (N)

Grading by End-Point:

**Mono CDVA:**	**A:** M **B:** M **C:** L **D:** L **E:** L **F:** M **G:** L **O:** M
**Mono DCIVA:**	**A:** M **B:** M **C:** L **D:** L **E:** L **F:** M **G:** L **O:** M
**Mono DCNVA:**	**A:** M **B:** M **C:** L **D:** L **E:** L **F:** M **G:** L **O:** M
**Mono DC:**	**A:** M **B:** M **C:** L **D:** L **E:** L **F:** M **G:** L **O:** M
**Mono CSF:**	**A:** N **B:** N **C:** N **D:** N **E:** N **F:** N **G:** N **O:** N
**Bino UDVA:**	**A:** N **B:** N **C:** N **D:** N **E:** N **F:** N **G:** N **O:** N
**Bino UIVA:**	**A:** N **B:** N **C:** N **D:** N **E:** N **F:** N **G:** N **O:** N
**Bino UNVA:**	**A:** N **B:** N **C:** N **D:** N **E:** N **F:** N **G:** N **O:** N
**PROs:**	**A:** M **B:** M **C:** L **D:** L **E:** L **F:** L **G:** M **O:** M
**Bino DC:**	**A:** N **B:** N **C:** N **D:** N **E:** N **F:** N **G:** N **O:** N
**Bino CSF:**	**A:** N **B:** N **C:** N **D:** N **E:** N **F:** N **G:** N **O:** N

Comments for Decision by Domain:

A. Confounding:

- A demographic table was included, showing no significant differences for selected confounders.
- However, discrepancies in the reported age between the table and the main text suggest an error.

B. Selection Bias:

- Patients who preferred to see intermediate distances selected the Intervention IOL, but this preference should not affect distance-corrected visual performance.

C. Classification:

- IOLs for both intervention and comparator clearly defined at the intervention stage.

D. Deviations:

- Described complications did not result in any deviations from the study.

E. Missing Data:

- Authors did not mention any missing data.

F. Measuring Outcomes:

- Tests used to measure outcomes, such as visual acuity, were not specified.

G. Reporting Outcomes:

- Authors reported levels of spectacle independence, but the methods did not describe using any specific questionnaire for this.

Study: Huh 2021 (Tecnis ZCB00)

Comparator: Tecnis ZCB00

Risk of Bias Tool: ROBINS-I

Correspondence: Low (L) Moderate (M) Serious (S) Critical (C) Not Informed (N)

Grading by End-Point:

**Mono CDVA:**	**A:** C **B:** M **C:** L **D:** L **E:** L **F:** M **G:** M **O:** C
**Mono DCIVA:**	**A:** N **B:** N **C:** N **D:** N **E:** N **F:** N **G:** N **O:** N
**Mono DCNVA:**	**A:** N **B:** N **C:** N **D:** N **E:** N **F:** N **G:** N **O:** N
**Mono DC:**	**A:** N **B:** N **C:** N **D:** N **E:** N **F:** N **G:** N **O:** N
**Mono CSF:**	**A:** N **B:** N **C:** N **D:** N **E:** N **F:** N **G:** N **O:** N
**Bino UDVA:**	**A:** N **B:** N **C:** N **D:** N **E:** N **F:** N **G:** N **O:** N
**Bino UIVA:**	**A:** C **B:** M **C:** L **D:** L **E:** L **F:** M **G:** M **O:** C
**Bino UNVA:**	**A:** C **B:** M **C:** L **D:** L **E:** L **F:** M **G:** M **O:** C
**PROs:**	**A:** C **B:** M **C:** L **D:** L **E:** L **F:** M **G:** M **O:** C
**Bino DC:**	**A:** C **B:** M **C:** L **D:** L **E:** L **F:** M **G:** M **O:** C
**Bino CSF:**	**A:** N **B:** N **C:** N **D:** N **E:** N **F:** N **G:** N **O:** N

Comments for Decision by Domain:

A. Confounding:

- A demographic table was presented, showing that the control group consisted of shorter eyes.
- It has been demonstrated that the intervention lens performs better in shorter eyes, leading to a critical risk of bias in favor of the control group.

B. Selection Bias:

- Patients with intraoperative or postoperative complications were excluded, but the number of excluded eyes per group was not reported, leading to potential selection bias.

C. Classification:

- IOLs for both intervention and comparator groups clearly defined at the intervention stage.

D. Deviations:

- Patients with complications were excluded at the preoperative stage.
- No other reasons for loss of follow-up were mentioned.

E. Missing Data:

- Authors did not mention any missing data.

F. Measuring Outcomes:

- All measurements were conducted similarly in both groups, but it was not specified if standard procedures were followed.

G. Reporting Outcomes:

- Only binocular visual acuities were reported, making not possible to test IOL efficacy.

Study: Kang 2021 (Tecnis ZCB00)

Comparator: Tecnis ZCB00

Risk of Bias Tool: ROBINS-I

Correspondence: Low (L) Moderate (M) Serious (S) Critical (C) Not Informed (N)

Grading by End-Point:

Mono CDVA: **A:** N **B:** N **C:** N **D:** N **E:** N **F:** N **G:** N **O:** N

Mono DCIVA: **A:** N **B:** N **C:** N **D:** N **E:** N **F:** N **G:** N **O:** N

Mono DCNVA: **A:** N **B:** N **C:** N **D:** N **E:** N **F:** N **G:** N **O:** N

Mono DC: **A:** M **B:** M **C:** L **D:** L **E:** L **F:** M **G:** M **O:** M

Mono CSF: **A:** N **B:** N **C:** N **D:** N **E:** N **F:** N **G:** N **O:** N

Bino UDVA: **A:** N **B:** N **C:** N **D:** N **E:** N **F:** N **G:** N **O:** N

Bino UIVA: **A:** N **B:** N **C:** N **D:** N **E:** N **F:** N **G:** N **O:** N

Bino UNVA: **A:** N **B:** N **C:** N **D:** N **E:** N **F:** N **G:** N **O:** N

PROs: **A:** N **B:** N **C:** N **D:** N **E:** N **F:** N **G:** N **O:** N

Bino DC: **A:** N **B:** N **C:** N **D:** N **E:** N **F:** N **G:** N **O:** N

Bino CSF: **A:** N **B:** N **C:** N **D:** N **E:** N **F:** N **G:** N **O:** N

Comments for Decision by Domain:

A. Confounding:

- A demographic table was presented, but few potential confounding factors were tested.
- Important factors such as biometric eye characteristics were excluded.

B. Selection Bias:

- No description provided for how participants were assigned to each group.

C. Classification:

- IOLs for both intervention and comparator groups clearly defined at the intervention stage.

D. Deviations:

- No deviations were mentioned after the intervention, and no complications or adverse events were reported.

E. Missing Data:

- Authors did not mention any missing data.

F. Measuring Outcomes:

- Authors did not specify if the defocus curve was measured with best distance correction, but postoperative spherical equivalent was reported with no differences between groups.

G. Reporting Outcomes:

- Authors reported all monocular end-points without distance correction.

Study: Cinar 2021 (AcrySof SN60WF)

Comparator: AcrySof SN60WF

Risk of Bias Tool: ROBINS-I

Correspondence: Low (L) Moderate (M) Serious (S) Critical (C) Not Informed (N)

Grading by End-Point:

Mono CDVA: **A:** M **B:** M **C:** L **D:** L **E:** L **F:** M **G:** M **O:** M

Mono DCIVA: **A:** M **B:** M **C:** L **D:** L **E:** L **F:** M **G:** M **O:** M

Mono DCNVA: **A:** M **B:** M **C:** L **D:** L **E:** L **F:** M **G:** M **O:** M

Mono DC: **A:** N **B:** N **C:** N **D:** N **E:** N **F:** N **G:** N **O:** N

Mono CSF: **A:** N **B:** N **C:** N **D:** N **E:** N **F:** N **G:** N **O:** N

Bino UDVA: **A:** N **B:** N **C:** N **D:** N **E:** N **F:** N **G:** N **O:** N

Bino UIVA: **A:** N **B:** N **C:** N **D:** N **E:** N **F:** N **G:** N **O:** N

Bino UNVA: **A:** N **B:** N **C:** N **D:** N **E:** N **F:** N **G:** N **O:** N

PROs: **A:** M **B:** M **C:** L **D:** L **E:** L **F:** M **G:** M **O:** M

Bino DC: **A:** N **B:** N **C:** N **D:** N **E:** N **F:** N **G:** N **O:** N

Bino CSF: **A:** N **B:** N **C:** N **D:** N **E:** N **F:** N **G:** N **O:** N

Comments for Decision by Domain:

A. Confounding:

- A demographic table was presented, but few potential confounding factors were tested.
- Important factors such as biometric eye characteristics were excluded.

B. Selection Bias:

- No description was provided for assigning participants to each group.
- Patients with intraoperative or postoperative complications were excluded, but the number of excluded eyes per group was not reported, leading to possible selection bias.

C. Classification:

- IOLs for both intervention and comparator groups clearly defined at the intervention stage.

D. Deviations:

- No deviations were mentioned after the intervention.
- Complications were excluded at the preoperative stage.

E. Missing Data:

- Authors did not mention any missing data.

F. Measuring Outcomes:

- Standard procedures of measurement were followed in both groups.

G. Reporting Outcomes:

- All outcomes that were measured were reported by the authors.

Study: Unsal 2021 (Tecnis ZCB00)

Comparator: Tecnis ZCB00

Risk of Bias Tool: ROBINS-I

Correspondence: Low (L) Moderate (M) Serious (S) Critical (C) Not Informed (N)

Grading by End-Point:

**Mono CDVA:**	**A:** C **B:** M **C:** L **D:** L **E:** L **F:** S **G:** M **O:** C
**Mono DCIVA:**	**A:** C **B:** M **C:** L **D:** L **E:** L **F:** S **G:** M **O:** C
**Mono DCNVA:**	**A:** C **B:** M **C:** L **D:** L **E:** L **F:** S **G:** M **O:** C
**Mono DC:**	**A:** N **B:** N **C:** N **D:** N **E:** N **F:** N **G:** N **O:** N
**Mono CSF:**	**A:** N **B:** N **C:** N **D:** N **E:** N **F:** N **G:** N **O:** N
**Bino UDVA:**	**A:** N **B:** N **C:** N **D:** N **E:** N **F:** N **G:** N **O:** N
**Bino UIVA:**	**A:** C **B:** M **C:** L **D:** L **E:** L **F:** S **G:** M **O:** C
**Bino UNVA:**	**A:** N **B:** N **C:** N **D:** N **E:** N **F:** N **G:** N **O:** N
**PROs:**	**A:** C **B:** M **C:** L **D:** L **E:** L **F:** S **G:** M **O:** C
**Bino DC:**	**A:** C **B:** M **C:** L **D:** L **E:** L **F:** S **G:** M **O:** C
**Bino CSF:**	**A:** C **B:** M **C:** L **D:** L **E:** L **F:** S **G:** M **O:** C

Comments for Decision by Domain:

A. Confounding:

- Methods for assigning interventions to participants were not described.
- No biometric factors were controlled.
- Possible bias introduced if objective refraction was used for best distance correction due to differences in postoperative refractive error distribution between groups.

B. Selection Bias:

- No description was provided for assigning participants to each group.
- Inclusion and exclusion criteria do not suggest any selection bias.

C. Classification:

- IOLs for both intervention and comparator groups clearly defined at the intervention stage.

D. Deviations:

- No deviations were mentioned after the intervention.

E. Missing Data:

- All patients included were analyzed, with no missing data for the reported endpoint.

F. Measuring Outcomes:

- VA: Non-standard methods were used in both groups, particularly for near vision, where a reading chart was used.
- DC and CSF were measured only binocularly as secondary outcomes.

G. Reporting Outcomes:

- Binocular outcomes for DC and CSF were reported.
- CSF was incorrectly analyzed by averaging patches instead of using logCS units, leading to critical bias in CSF results.
- Although the study described a 5-month follow-up, reported outcomes were for the 1-month mark.

Study: Elbakry 2023 (Tecnis ZCB00)

Comparator: Tecnis ZCB00

Risk of Bias Tool: ROBINS-I

Correspondence: Low (L) Moderate (M) Serious (S) Critical (C) Not Informed (N)

Grading by End-Point:

Mono CDVA: **A:** N **B:** N **C:** N **D:** N **E:** N **F:** N **G:** N **O:** N

Mono DCIVA: **A:** N **B:** N **C:** N **D:** N **E:** N **F:** N **G:** N **O:** N

Mono DCNVA: **A:** N **B:** N **C:** N **D:** N **E:** N **F:** N **G:** N **O:** N

Mono DC: **A:** N **B:** N **C:** N **D:** N **E:** N **F:** N **G:** N **O:** N

Mono CSF: **A:** N **B:** N **C:** N **D:** N **E:** N **F:** N **G:** N **O:** N

Bino UDVA: **A:** C **B:** M **C:** L **D:** L **E:** L **F:** L **G:** L **O:** C

Bino UIVA: **A:** C **B:** M **C:** L **D:** L **E:** L **F:** C **G:** C **O:** C

Bino UNVA: **A:** N **B:** N **C:** N **D:** N **E:** N **F:** N **G:** N **O:** N

PROs: **A:** C **B:** M **C:** L **D:** L **E:** L **F:** C **G:** L **O:** C

Bino DC: **A:** C **B:** M **C:** L **D:** L **E:** L **F:** C **G:** C **O:** C

Bino CSF: **A:** N **B:** N **C:** N **D:** N **E:** N **F:** N **G:** N **O:** N

Comments for Decision by Domain:

A. Confounding:

- Methods for assigning interventions to participants were not described.
- No biometric factors were controlled, including basic ones such as the age. Postoperative refractions resulted in higher postoperative myopic refraction in monofocal group which might underestimate the differences in binocular UIVA.

B. Selection Bias:

- No description was provided for assigning participants to each group.
- Inclusion and exclusion criteria do not suggest any selection bias.

C. Classification:

- IOLs for both intervention and comparator groups clearly defined at the intervention stage.

D. Deviations:

- No deviations were mentioned after the intervention.

E. Missing Data:

- All patients included were analyzed, with no missing data for the reported endpoint.

F. Measuring Outcomes:

- VA: Non-standard methods were used in both groups, particularly for intermediate and near vision, where jaeger charts were used according to the registered protocol.
- DC was measured only binocularly as secondary outcomes.
- It is not clearly specified how questions were conducted for assessment of photic ophenomena.

G. Reporting Outcomes:

- Near outcomes were not reported even though this measurement was included in the protocol.
- Binocular outcomes for DC were reported, but it was not specified if measurements were conducted with distance correction and the 0 D and -0.50 D defocus points were omitted.
- Contrast sensitivity was measured with Pelli Robson that measures low spatial frequencies.

Study: Eguileor 2020 (Tecnis ZCB00)

Comparator: Tecnis ZCB00

Risk of Bias Tool: RoB-2

Correspondence: Low (L) Some Concerns (S) High (H) Not Applicable (N)

Grading by End-Point:

**Mono CDVA:**	**A:** S **B:** S **C:** L **D:** S **E:** L **F:** N **G:** N **O:** S
**Mono DCIVA:**	**A:** S **B:** S **C:** L **D:** S **E:** L **F:** N **G:** N **O:** S
**Mono DCNVA:**	**A:** N **B:** N **C:** N **D:** N **E:** N **F:** N **G:** N **O:** N
**Mono DC:**	**A:** N **B:** N **C:** N **D:** N **E:** N **F:** N **G:** N **O:** N
**Mono CSF:**	**A:** N **B:** N **C:** N **D:** N **E:** N **F:** N **G:** N **O:** N
**Bino UDVA:**	**A:** N **B:** N **C:** N **D:** N **E:** N **F:** N **G:** N **O:** N
**Bino UIVA:**	**A:** N **B:** N **C:** N **D:** N **E:** N **F:** N **G:** N **O:** N
**Bino UNVA:**	**A:** N **B:** N **C:** N **D:** N **E:** N **F:** N **G:** N **O:** N
**PROs:**	**A:** N **B:** N **C:** N **D:** N **E:** N **F:** N **G:** N **O:** N
**Bino DC:**	**A:** S **B:** S **C:** L **D:** S **E:** L **F:** N **G:** N **O:** S
**Bino CSF:**	**A:** N **B:** N **C:** N **D:** N **E:** N **F:** N **G:** N **O:** N

Comments for Decision by Domain:

A. Randomisation:

- Randomized process mentioned, but details not specified
- No information on allocation concealment
- Spherical aberration at 6 mm mentioned, could influence depth of focus, but no reliable data for 4 mm SA

B. Deviations:

- Not reported if participants or caregivers were aware of the assigned intervention
- No mention of adverse events requiring non-protocol intervention
- No complications during surgery reported

C. Missing Data:

- No exclusions for analysis were reported

D. Measuring Outcomes:

- Standard ETDRS chart used for both groups. Unlikely the measurement outcome differed between groups
- Masking of assessors not reported

- As the ETDRS is a chart, the assessors decide the threshold. It can by a tendency by the assessor to select a more favorable threshold. But it is unlikely that this happens for differences of a line of visual acuity.

E. Reporting Outcomes:

- Primary endpoint is intermediate visual acuity, though plan not provided. Best distance correction selected for these cases

Study: Giglio, 2024a (Tecnis PCB00) 2024b (Clareon CNA0T0)

Comparator: Tecnis PCB00 and Clareon CNA0T0

Risk of Bias Tool: RoB-2

Correspondence: Low (L) Some Concerns (S) High (H) Not Applicable (N)

Grading by End-Point:

**Mono CDVA:**	**A:** S **B:** S **C:** L **D:** S **E:** L **F:** N **G:** N **O:** S
**Mono DCIVA:**	**A:** S **B:** S **C:** L **D:** S **E:** L **F:** N **G:** N **O:** S
**Mono DCNVA:**	**A:** S **B:** S **C:** L **D:** S **E:** L **F:** N **G:** N **O:** S
**Mono DC:**	**A:** N **B:** N **C:** N **D:** N **E:** N **F:** N **G:** N **O:** N
**Mono CSF:**	**A:** N **B:** N **C:** N **D:** N **E:** N **F:** N **G:** N **O:** N
**Bino UDVA:**	**A:** S **B:** S **C:** L **D:** S **E:** L **F:** N **G:** N **O:** S
**Bino UIVA:**	**A:** S **B:** S **C:** L **D:** S **E:** L **F:** N **G:** N **O:** S
**Bino UNVA:**	**A:** N **B:** N **C:** N **D:** N **E:** N **F:** N **G:** N **O:** N
**PROs:**	**A:** N **B:** N **C:** N **D:** N **E:** N **F:** N **G:** N **O:** N
**Bino DC:**	**A:** S **B:** S **C:** L **D:** S **E:** L **F:** N **G:** N **O:** S
**Bino CSF:**	**A:** N **B:** N **C:** N **D:** N **E:** N **F:** N **G:** N **O:** N

Comments for Decision by Domain:

A. Randomisation:

- Patients randomized by block randomization with a 1:1:1 allocation ratio
- Sealed Envelope Ltd. used for randomization with unique codes for each patient
- CNA0T0 group was significantly older than others, though not compared to PCB00 group

B. Deviations:

- Study was single-masked (Investigator was masked as per protocol NCT06118944)
- No information on adverse events requiring non-protocol interventions
- Study conducted as planned
- One-way ANOVA with partial eta squared used to compare clinical and demographic variables

C. Missing Data:

- 90 patients enrolled, 30 in each group, all completed follow-up

D. Measuring Outcomes:

- Standard ETDRS charts used for pre- and postoperative visual acuity
- Single-masked study
- Evaluator decides when to stop and how to record acuity with the static chart

E. Reporting Outcomes:

- Primary endpoint is intermediate visual acuity, though plan not provided
- Best distance correction selected for reporting outcomes.

Study: Goslings 2023 (Vivinex iSert)

Comparator: Vivinex iSert

Risk of Bias Tool: RoB-2

Correspondence: Low (L) Some Concerns (S) High (H) Not Applicable (N)

Grading by End-Point:

Mono CDVA: **A:** S **B:** S **C:** L **D:** S **E:** L **F:** N **G:** N **O:** S

Mono DCIVA: **A:** S **B:** S **C:** L **D:** S **E:** L **F:** N **G:** N **O:** S

Mono DCNVA: **A:** N **B:** N **C:** N **D:** N **E:** N **F:** N **G:** N **O:** N

Mono DC: **A:** N **B:** N **C:** N **D:** N **E:** N **F:** N **G:** N **O:** N

Mono CSF: **A:** N **B:** N **C:** N **D:** N **E:** N **F:** N **G:** N **O:** N

Bino UDVA: **A:** S **B:** S **C:** L **D:** S **E:** L **F:** N **G:** N **O:** S

Bino UIVA: **A:** S **B:** S **C:** L **D:** S **E:** L **F:** N **G:** N **O:** S

Bino UNVA: **A:** N **B:** N **C:** N **D:** N **E:** N **F:** N **G:** N **O:** N

PROs: **A:** N **B:** N **C:** N **D:** N **E:** N **F:** N **G:** N **O:** N

Bino DC: **A:** N **B:** N **C:** N **D:** N **E:** N **F:** N **G:** N **O:** N

Bino CSF: **A:** N **B:** N **C:** N **D:** N **E:** N **F:** N **G:** N **O:** N

Comments for Decision by Domain:

A. Randomisation:

- Patients received randomization numbers from a computerized random number generator
- No information on whether the allocation sequence was concealed
- Demographic variables included age and CDVA, no differences among groups

B. Deviations:

- No complications or adverse events reported that may suggest non-protocol interventions

C. Missing Data:

- It is not explicitly stated whether outcome data were available for all participants

D. Measuring Outcomes:

- ETDRS used for both distance and intermediate measurements
- No mention of masking
- Evaluator determines when to stop and how to record acuity using the static chart

E. Reporting Outcomes:

- Protocol sent to the ethics committee (EC) but not published

- Selected outcomes are appropriate for the study type, reducing likelihood of bias

Study: Donoso 2024 (Tecnis ZCB00)

Comparator: Vivinex iSert

Risk of Bias Tool: RoB-2

Correspondence: Low (L) Some Concerns (S) High (H) Not Applicable (N)

Grading by End-Point:

Mono CDVA: **A:** L **B:** L **C:** L **D:** L **E:** L **F:** N **G:** N **O:** L

Mono DCIVA: **A:** L **B:** L **C:** L **D:** L **E:** L **F:** N **G:** N **O:** L

Mono DCNVA: **A:** N **B:** N **C:** N **D:** N **E:** N **F:** N **G:** N **O:** N

Mono DC: **A:** N **B:** N **C:** N **D:** N **E:** N **F:** N **G:** N **O:** N

Mono CSF: **A:** N **B:** N **C:** N **D:** N **E:** N **F:** N **G:** N **O:** N

Bino UDVA: **A:** L **B:** L **C:** L **D:** L **E:** L **F:** N **G:** N **O:** L

Bino UIVA: **A:** L **B:** L **C:** L **D:** L **E:** L **F:** N **G:** N **O:** L

Bino UNVA: **A:** L **B:** L **C:** L **D:** L **E:** L **F:** N **G:** N **O:** L

PROs: **A:** L **B:** L **C:** L **D:** L **E:** L **F:** N **G:** N **O:** L

Bino DC: **A:** L **B:** L **C:** L **D:** L **E:** L **F:** N **G:** N **O:** L

Bino CSF: **A:** N **B:** N **C:** N **D:** N **E:** N **F:** N **G:** N **O:** N

Comments for Decision by Domain:

A. Randomisation:

- Patients were randomly assigned using a published algorithm in Stata MP 14
- Randomization was masked during surgery and postoperative evaluations
- Non-clinical and statistically significant differences observed among groups

B. Deviations:

- Patient and vision assessor-masked randomized clinical trial

C. Missing Data:

- A 10% loss to follow-up was expected, with 30 patients per group achieving the required sample size
- Losses were balanced across groups

D. Measuring Outcomes:

- ETDRS near vision chart used for intermediate and near vision measurement

E. Reporting Outcomes:

- Protocol passed to the ethics committee (EC) but not publicly available. Authors reported CNVA and DCNVA in different parts of the manuscript but the outcomes are

more aligned with CNVA since this value is close to the DCIVA when it should be considerably lower.

Study: Garzón 2022 (Tecnis ZCB00)

Comparator: Tecnis ZCB00

Risk of Bias Tool: RoB-2

Correspondence: Low (L) Some Concerns (S) High (H) Not Applicable (N)

Grading by End-Point:

**Mono CDVA:**	**A:** S **B:** L **C:** L **D:** L **E:** L **F:** N **G:** N **O:** S
**Mono DCIVA:**	**A:** N **B:** N **C:** N **D:** N **E:** N **F:** N **G:** N **O:** N
**Mono DCNVA:**	**A:** N **B:** N **C:** N **D:** N **E:** N **F:** N **G:** N **O:** N
**Mono DC:**	**A:** N **B:** N **C:** N **D:** N **E:** N **F:** N **G:** N **O:** N
**Mono CSF:**	**A:** S **B:** L **C:** L **D:** L **E:** L **F:** N **G:** N **O:** S
**Bino UDVA:**	**A:** N **B:** N **C:** N **D:** N **E:** N **F:** N **G:** N **O:** N
**Bino UIVA:**	**A:** N **B:** N **C:** N **D:** N **E:** N **F:** N **G:** N **O:** N
**Bino UNVA:**	**A:** N **B:** N **C:** N **D:** N **E:** N **F:** N **G:** N **O:** N
**PROs:**	**A:** N **B:** N **C:** N **D:** N **E:** N **F:** N **G:** N **O:** N
**Bino DC:**	**A:** N **B:** N **C:** N **D:** N **E:** N **F:** N **G:** N **O:** N
**Bino CSF:**	**A:** N **B:** N **C:** N **D:** N **E:** N **F:** N **G:** N **O:** N

Comments for Decision by Domain:

A. Randomisation:

- Randomization details not provided, only stated that patients were randomized and blinded
- Differences in age, with older patients in the control group, potentially favoring the control group

B. Deviations:

- No specific details provided on deviations from intended interventions

C. Missing Data:

- No complications reported, and no mention of follow-up loss

D. Measuring Outcomes:

- Standard methods of measurement were used

E. Reporting Outcomes:

- Measurements and reports aligned with the study's focus on far distance vision

Study: Nanavaty 2022 (Tecnis ZCB00)

Comparator: Tecnis ZCB00

Risk of Bias Tool: RoB-2

Correspondence: Low (L) Some Concerns (S) High (H) Not Applicable (N)

Grading by End-Point:

**Mono CDVA:**	**A:** S **B:** H **C:** L **D:** S **E:** H **F:** N **G:** N **O:** H
**Mono DCIVA:**	**A:** S **B:** H **C:** L **D:** S **E:** H **F:** N **G:** N **O:** H
**Mono DCNVA:**	**A:** N **B:** N **C:** N **D:** N **E:** N **F:** N **G:** N **O:** N
**Mono DC:**	**A:** S **B:** H **C:** L **D:** S **E:** H **F:** N **G:** N **O:** H
**Mono CSF:**	**A:** N **B:** N **C:** N **D:** N **E:** N **F:** N **G:** N **O:** N
**Bino UDVA:**	**A:** S **B:** H **C:** L **D:** S **E:** H **F:** N **G:** N **O:** H
**Bino UIVA:**	**A:** S **B:** H **C:** L **D:** S **E:** H **F:** N **G:** N **O:** H
**Bino UNVA:**	**A:** N **B:** N **C:** N **D:** N **E:** N **F:** N **G:** N **O:** N
**PROs:**	**A:** S **B:** H **C:** L **D:** S **E:** H **F:** N **G:** N **O:** H
**Bino DC:**	**A:** S **B:** H **C:** L **D:** S **E:** H **F:** N **G:** N **O:** H
**Bino CSF:**	**A:** N **B:** N **C:** N **D:** N **E:** N **F:** N **G:** N **O:** N

Comments for Decision by Domain:

A. Randomisation:

- Patients randomly allocated using an online random number generator by a non-involved team member
- Research team informed of the intervention just before surgery
- Two sets of envelopes used, with participants choosing surgery timing (same day or within 2 weeks)
- Inconsistencies between mean corneal astigmatism and mean keratometry in the intervention group

B. Deviations:

- Evaluators were not reported as masked
- Additional corneal incisions may have been unbalanced between groups
- No sub-analysis conducted to assess the influence of corneal incisions on the outcomes

C. Missing Data:

- Few follow-up losses, unlikely to affect the outcome

D. Measuring Outcomes:

- Standard methods of measurement used

- Evaluator decides the threshold of visual acuity, representing a potential (but unlikely) risk of bias

E. Reporting Outcomes:

- Protocol registered (NCT04175951) but breached by recruiting patients with astigmatism above 1.5 D
- Protocol described 60 cm for DCIVA, modified to 66 cm in publication

Study: Auffarth 2021 (Tecnis ZCB00)

Comparator: Tecnis ZCB00

Risk of Bias Tool: RoB-2

Correspondence: Low (L) Some Concerns (S) High (H) Not Applicable (N)

Grading by End-Point:

Mono CDVA: **A:** L **B:** L **C:** L **D:** L **E:** L **F:** N **G:** N **O:** L

Mono DCIVA: **A:** L **B:** L **C:** L **D:** L **E:** L **F:** N **G:** N **O:** L

Mono DCNVA: **A:** N **B:** N **C:** N **D:** N **E:** N **F:** N **G:** N **O:** N

Mono DC: **A:** N **B:** N **C:** N **D:** N **E:** N **F:** N **G:** N **O:** N

Mono CSF: **A:** N **B:** N **C:** N **D:** N **E:** N **F:** N **G:** N **O:** N

Bino UDVA: **A:** L **B:** L **C:** L **D:** L **E:** L **F:** N **G:** N **O:** L

Bino UIVA: **A:** L **B:** L **C:** L **D:** L **E:** L **F:** N **G:** N **O:** L

Bino UNVA: **A:** N **B:** N **C:** N **D:** N **E:** N **F:** N **G:** N **O:** N

PROs: **A:** L **B:** L **C:** L **D:** L **E:** L **F:** N **G:** N **O:** L

Bino DC: **A:** L **B:** L **C:** L **D:** L **E:** L **F:** N **G:** N **O:** L

Bino CSF: **A:** N **B:** N **C:** N **D:** N **E:** N **F:** N **G:** N **O:** N

Comments for Decision by Domain:

A. Randomisation:

- Patients were randomly assigned using a centralized electronic randomization system (Merge eClinical OS)
- Patients and study technicians performing postoperative tests remained masked
- Groups were described as balanced, but no statistical tests were used, and the intervention group was slightly younger

B. Deviations:

- Patients and study technicians remained masked throughout the study

C. Missing Data:

- There was follow-up loss, but it was negligible considering the sample size

D. Measuring Outcomes:

- Standard testing methods were used

E. Reporting Outcomes:

- The protocol was registered and followed

Study: Choi 2023 (Tecnis ZCB00)

Comparator: Tecnis ZCB00

Risk of Bias Tool: RoB-2

Correspondence: Low (L) Some Concerns (S) High (H) Not Applicable (N)

Grading by End-Point:

**Mono CDVA:**	**A:** S **B:** S **C:** L **D:** S **E:** L **F:** N **G:** N **O:** S
**Mono DCIVA:**	**A:** N **B:** N **C:** N **D:** N **E:** N **F:** N **G:** N **O:** N
**Mono DCNVA:**	**A:** N **B:** N **C:** N **D:** N **E:** N **F:** N **G:** N **O:** N
**Mono DC:**	**A:** N **B:** N **C:** N **D:** N **E:** N **F:** N **G:** N **O:** N
**Mono CSF:**	**A:** N **B:** N **C:** N **D:** N **E:** N **F:** N **G:** N **O:** N
**Bino UDVA:**	**A:** S **B:** S **C:** L **D:** S **E:** L **F:** N **G:** N **O:** S
**Bino UIVA:**	**A:** S **B:** S **C:** L **D:** S **E:** L **F:** N **G:** N **O:** S
**Bino UNVA:**	**A:** S **B:** S **C:** L **D:** S **E:** L **F:** N **G:** N **O:** S
**PROs:**	**A:** S **B:** S **C:** L **D:** S **E:** L **F:** N **G:** N **O:** S
**Bino DC:**	**A:** S **B:** S **C:** L **D:** S **E:** L **F:** N **G:** N **O:** S
**Bino CSF:**	**A:** N **B:** N **C:** N **D:** N **E:** N **F:** N **G:** N **O:** N

Comments for Decision by Domain:

A. Randomisation:

- Study described as randomized, but no details on the randomization process provided
- Demographic characteristics showed no differences between groups

B. Deviations:

- No information about masking procedures
- No adverse events reported, all recruited patients included in the analysis, implying no deviations

C. Missing Data:

- Number of patients and eyes included in the analysis is reported

D. Measuring Outcomes:

- Standard methods used for obtaining measurements
- ETDRS threshold depends on evaluator's decision

E. Reporting Outcomes:

- Measurements obtained without correction, likely aiming to assess the procedure's efficacy rather than the IOL efficacy
