# Supplementary material for: Visual and patient-reported outcomes of an enhanced versus monofocal intraocular lenses in cataract surgery: a systematic review and meta-analysis
Source: Eye (Lond). 2025 Feb 1;39(5):883–98. doi: 10.1038/s41433-025-03625-4 (PMC11933469; doi:10.1038/s41433-025-03625-4)
Supplement: Supplementary file 10 — Supplementary File C: Risk of Bias Assessment Plots [file 41433_2025_3625_MOESM10_ESM.pdf]

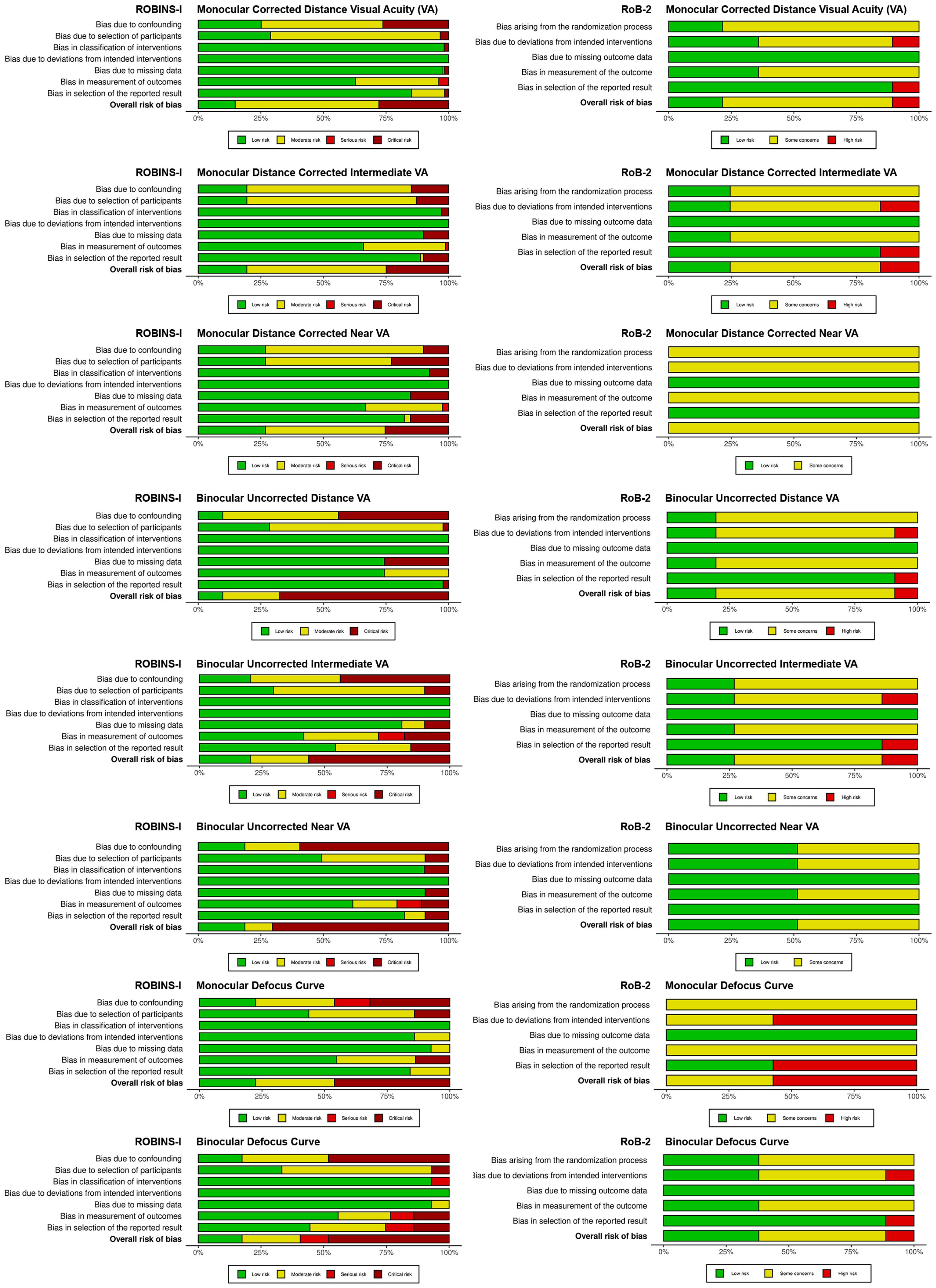
ROBINS-I
Monocular Corrected Distance Visual Acuity (VA)
Bias due to confounding
Bias due to selection of participants
Bias in classification of interventions
Bias due to deviations from intended interventions
Bias due to missing data
Bias in measurement of outcomes
Bias in selection of the reported result
Overall risk of bias
0% 25% 50% 75% 100%
Low risk Moderate risk Serious risk Critical risk
RoB-2
Monocular Corrected Distance Visual Acuity (VA)
Bias arising from the randomization process
Bias due to deviations from intended interventions
Bias due to missing outcome data
Bias in measurement of the outcome
Bias in selection of the reported result
Overall risk of bias
0% 25% 50% 75% 100%
Low risk Some concerns High risk
ROBINS-I
Monocular Distance Corrected Intermediate VA
RoB-2
Monocular Distance Corrected Intermediate VA
ROBINS-I
Monocular Distance Corrected Near VA
RoB-2
Monocular Distance Corrected Near VA
Low risk Some concerns
ROBINS-I
Binocular Uncorrected Distance VA
Low risk Moderate risk Critical risk
RoB-2
Binocular Uncorrected Distance VA
ROBINS-I
Binocular Uncorrected Intermediate VA
RoB-2
Binocular Uncorrected Intermediate VA
ROBINS-I
Binocular Uncorrected Near VA
RoB-2
Binocular Uncorrected Near VA
ROBINS-I
Monocular Defocus Curve
RoB-2
Monocular Defocus Curve
ROBINS-I
Binocular Defocus Curve
RoB-2
Binocular Defocus Curve

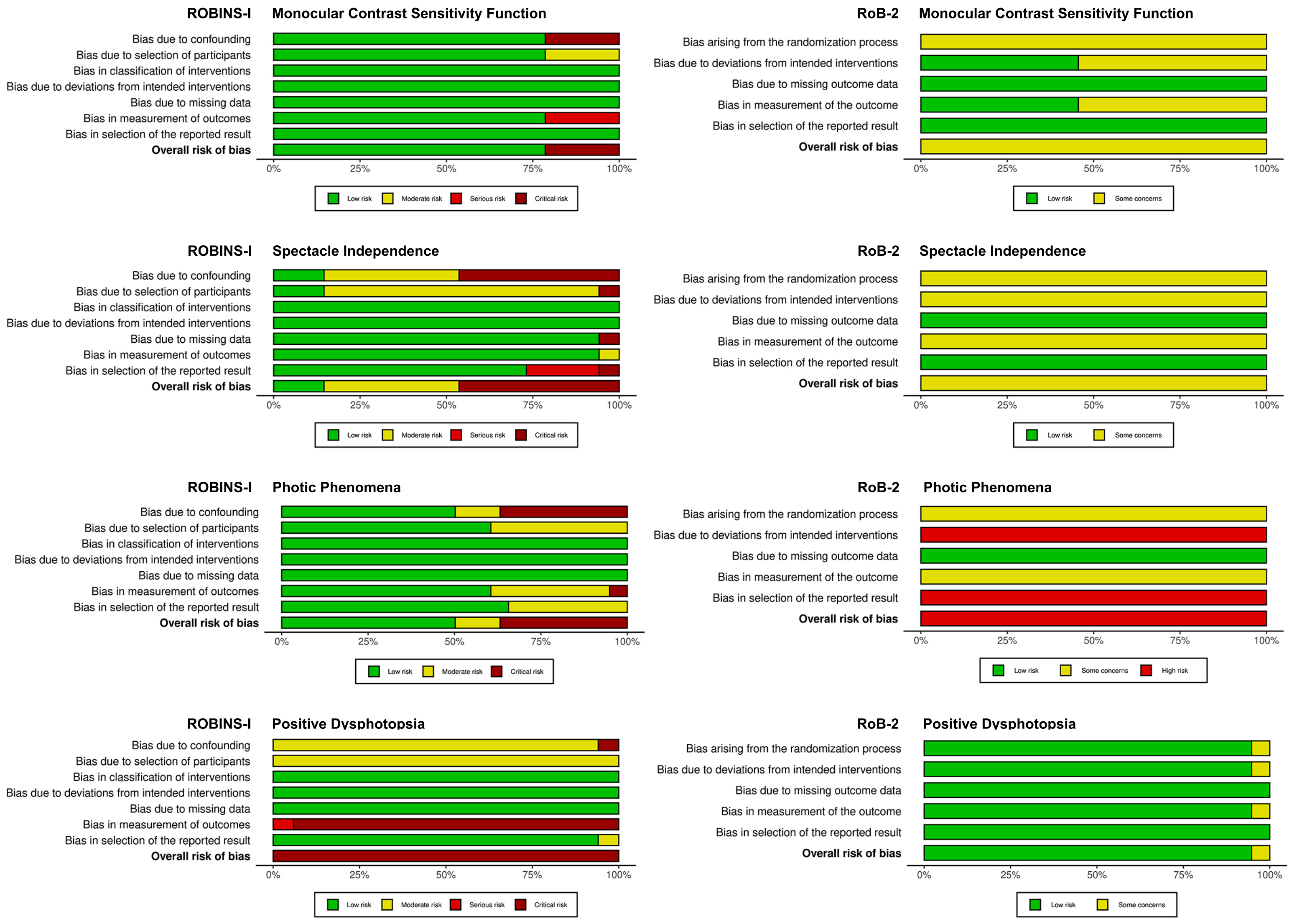
ROBINS-I
Monocular Contrast Sensitivity Function
Bias due to confounding
Bias due to selection of participants
Bias in classification of interventions
Bias due to deviations from intended interventions
Bias due to missing data
Bias in measurement of outcomes
Bias in selection of the reported result
Overall risk of bias
0%
25%
50%
75%
100%
Low risk
Moderate risk
Serious risk
Critical risk
RoB-2
Monocular Contrast Sensitivity Function
Bias arising from the randomization process
Bias due to deviations from intended interventions
Bias due to missing outcome data
Bias in measurement of the outcome
Bias in selection of the reported result
Overall risk of bias
0%
25%
50%
75%
100%
Low risk
Some concerns
ROBINS-I
Spectacle Independence
Bias due to confounding
Bias due to selection of participants
Bias in classification of interventions
Bias due to deviations from intended interventions
Bias due to missing data
Bias in measurement of outcomes
Bias in selection of the reported result
Overall risk of bias
0%
25%
50%
75%
100%
Low risk
Moderate risk
Serious risk
Critical risk
RoB-2
Spectacle Independence
Bias arising from the randomization process
Bias due to deviations from intended interventions
Bias due to missing outcome data
Bias in measurement of the outcome
Bias in selection of the reported result
Overall risk of bias
0%
25%
50%
75%
100%
Low risk
Some concerns
ROBINS-I
Photic Phenomena
Bias due to confounding
Bias due to selection of participants
Bias in classification of interventions
Bias due to deviations from intended interventions
Bias due to missing data
Bias in measurement of outcomes
Bias in selection of the reported result
Overall risk of bias
0%
25%
50%
75%
100%
Low risk
Moderate risk
Critical risk
RoB-2
Photic Phenomena
Bias arising from the randomization process
Bias due to deviations from intended interventions
Bias due to missing outcome data
Bias in measurement of the outcome
Bias in selection of the reported result
Overall risk of bias
0%
25%
50%
75%
100%
Low risk
Some concerns
High risk
ROBINS-I
Positive Dysphotopsia
Bias due to confounding
Bias due to selection of participants
Bias in classification of interventions
Bias due to deviations from intended interventions
Bias due to missing data
Bias in measurement of outcomes
Bias in selection of the reported result
Overall risk of bias
0%
25%
50%
75%
100%
Low risk
Moderate risk
Serious risk
Critical risk
RoB-2
Positive Dysphotopsia
Bias arising from the randomization process
Bias due to deviations from intended interventions
Bias due to missing outcome data
Bias in measurement of the outcome
Bias in selection of the reported result
Overall risk of bias
0%
25%
50%
75%
100%
Low risk
Some concerns
