# Supplementary material for: Visual and patient-reported outcomes of an enhanced versus monofocal intraocular lenses in cataract surgery: a systematic review and meta-analysis
Source: Eye (Lond). 2025 Feb 1;39(5):883–98. doi: 10.1038/s41433-025-03625-4 (PMC11933469; doi:10.1038/s41433-025-03625-4)
Supplement: Supplementary file 11 — Supplementary File D: Registered studies [file 41433_2025_3625_MOESM11_ESM.pdf]

NUMBER	TITLE	Comparator	URL	SPONSOR	CURRENT STATE	OUTCOMES IN
NCT06345820	Comparison of Two Non-diffractive Enhanced Monofocal Intraocular Lenses	Evolux	https://clinicaltrials.gov/study/NCT06345820?cond=Cataract&intr=enhanced%20&rank=4	Vienna Institute for Research in Ocular Surgery	Estimated completion 2025-06	Pending
NCT06118944	Outcomes of Bilateral Implantation of an Enhanced Monofocal Intraocular Lens (RCT_ICB00)	TecnisPCB00 & Clareon CNA0T0	https://clinicaltrials.gov/study/NCT06118944?cond=Cataract&intr=enhanced%20&rank=5	University of Trieste	Published Completion 2023-01-31	Giglio 2024
NCT04175951	Tecnis Eyhance Versus Rayner RayOne Study	RayOne Monofocal	https://clinicaltrials.gov/study/NCT04175951	Brighton and Sussex University Hospitals NHS Trust	Published Completion 2020-10-24	Nanavaty 2022
DRKS00010603	Clinical Investigation of the TECNIS® Extended Depth of Focus Intraocular Lens, Model ICB00	Tecnis ZCB00	https://trialsearch.who.int/Trial2.aspx?TrialID=DRKS00010603	Johnson & Johnson Surgical Vision, Inc.	Published Completion 29/06/2018	Auffarth 2021
NCT05481125	Clareon Toric vs Eyhance Toric	Clareon	https://clinicaltrials.gov/study/NCT05481125?intr=eyhance&rank=3	Alcon Research	Estimated completion 2023-10-16	Pending Submitted to registration page on 2024-10-07
NCT05025345	Vision Performance Evaluation of TECNIS EYHANCE™ With TECNIS SIMPLICITY™ Compared to TECNIS® 1-piece Intraocular Lenses	Tecnis ZCB00	https://clinicaltrials.gov/study/NCT05025345?intr=eyhance&rank=4&tab=results	Johnson & Johnson Surgical Vision, Inc.	Estimated completion 2022-06-17	Pending Published in registration page on 2023-08-28

NCT05875922	Comparison of ISOPURE and EYHANCE (Switzerland) (PHY2301)	IsoPure	https://clinicaltrials.gov/study/NCT05875922?intr=eyhance&rank=9	Beaver-Visitec International, Inc	Estimated completion 2025-02	Pending
NCT04800887	Visual Outcomes and Contrast Sensitivity in Patients Implanted With Tecnis Eyhance Intraocular Lens	Tecnis ZCB00	https://clinicaltrials.gov/study/NCT04800887?intr=eyhance&rank=11	Faculty Hospital Kralovske Vinohrady	Estimated completion 2021-08-24	Pending
NCT05226884	Intermediate Vision in Patients With Clareon IOLs Compared to Eyhance IOLs	Clareon	https://clinicaltrials.gov/study/NCT05226884?intr=eyhance&rank=12	Berkeley Eye Center	Published Completion 2022-08-06	Micheletti 2023
NCT05531110	Comparison of Two Aspheric Intraocular Lenses for Micro-monovision	Alcon IQ SN60WF	https://clinicaltrials.gov/study/NCT05531110?intr=eyhance&rank=14	Guy's and St Thomas' NHS Foundation Trust	Estimated completion 2024-12	Pending
NCT05506553	Comparison of Aspheric Toric Intraocular Lenses for Micro-monovision	Acrysof IQ	https://clinicaltrials.gov/study/NCT05506553?intr=eyhance&rank=15	Guy's and St Thomas' NHS Foundation Trust	Estimated completion 2024-09	Pending
NCT05430295	Visual Acuity After Using A New Monofocal Intraocular Lens Compared To Standard Monofocal Lens	Tecnis ZCB00	https://clinicaltrials.gov/study/NCT05430295?intr=eyhance&rank=19	Kasr El Aini Hospital	Published Completion 2021-11-01	Elbakry 2023
NCT04916041	Visual Function in Subjects Implanted With Advanced Technology Intraocular Lenses	Rayone EMV	https://clinicaltrials.gov/study/NCT04916041?intr=eyhance&rank=20#contacts-and-locations	Clínica Rementería	Estimated completion 2023-09-01	Pending
NCT05396599	A Clinical Study Comparing Postoperative Outcomes Between the TECNIS Intraocular Lens.	Tecnis ZCT	https://clinicaltrials.gov/study/NCT05396599?intr=eyhance&rank=22	Johnson & Johnson Surgical Vision, Inc.	Estimated completion 2023-04-07	Pending
